# The effect of tablet-based multimodal training on cognitive functioning in Alzheimer’s disease: A randomized controlled trial

**DOI:** 10.1371/journal.pone.0329931

**Published:** 2025-08-13

**Authors:** Julia Zuschnegg, Stefan Ropele, Peter Opriessnig, Reinhold Schmidt, Silvia Russegger, Maria Fellner, Manuel Leitner, Stephan Spat, Manuel Leal Garcia, Bernhard Strobl, Karin Ploder, Martin Pszeida, Maria M. Hofmarcher-Holzhacker, Eva Stoegerer-Oberschmid, Alexandra Guttmann-Lattmanig, Lucas Paletta, Sandra Schüssler, Marisa Koini

**Affiliations:** 1 Institute of Social Medicine and Epidemiology, Medical University of Graz, Graz, Austria; 2 Department of Neurology, Division of Neurogeriatrics, Medical University of Graz, Graz, Austria; 3 Institute Digital, Joanneum Research Forschungsgesellschaft mbH, Graz, Austria; 4 digitAAL Life GmbH, Graz, Austria; 5 Department of Medical Psychology, Psychosomatics, and Psychotherapy, Medical University of Graz, Graz, Austria; 6 Probando GmbH, Graz, Austria; 7 Austrian Red Cross Organization, Styrian branch, Graz, Austria; 8 HS&I Health System Intelligence, Vienna, Austria; University at Buffalo, UNITED STATES OF AMERICA

## Abstract

**Background:**

Computerized cognitive training conducted in a home setting has shown beneficial effects on cognitive functions in people with mild cognitive impairment. Similar effects in people suffering from Alzheimer’s disease (AD) have not yet been found. We aimed to examine the effect of a six-month tablet-based multimodal training in community-dwelling people with mild to moderate AD on cognitive functions and on the volume of (sub)cortical structures.

**Methods:**

Within a randomized controlled trial, a six-month (un)supervised tablet-based multimodal training including cognitive and physical exercises in people with mild to moderate AD (n = 11) was compared to a control group (n = 11) that received cognitive paper-and-pencil exercises for voluntary, unsupervised training. Participants in the intervention group were visited by professional trainers on a weekly basis for joint, supervised training sessions and were encouraged to train alone or with a caregiver as often as possible. Neuropsychological examination included assessments of global cognitive functions, memory, attention, executive functions, and verbal fluency. Freesurfer analyses of T1-weighted scans from structural magnetic resonance imaging were used to assess volumes of specific (sub)cortical areas (e.g., hippocampal volume).

**Results:**

Over six months, the intervention group showed a stable global cognitive function score (Mini Mental Status Examination), whereas the control group showed a cognitive decline (ANCOVA-interaction: F1, 14 = 5.083, p = .041; controlled for disease duration and education). No other selective cognitive domain showed a significant time-by-group difference. No difference in cerebral volumes were detected.

**Conclusion:**

The tablet-based multimodal training with cognitive and physical activation has positive effects on global cognitive functions of people with mild to moderate AD over a six-month training period, but lacks measurable transferability to other cognitive domains such as memory, attention or executive functions or brain structure. Further research on such interventions using high-quality longitudinal designs is recommended.

**Trial registration:**

ClinicalTrials.gov (NCT04628702).

## Introduction

Alzheimer’s disease (AD) is a neurocognitive and progressive disease characterized by functional decline and cognitive impairment in global cognition and neuropsychological domains such as memory, language, executive functions or problem solving [1–3]. It affects activities of daily living (ADL), self-determination and quality of life (Qol) [4,5]. The deterioration of cognitive abilities leads to a high degree of care dependency and loss of independence, respectively [6,7]. Incipient, cerebral atrophy of the hippocampal areas is a major hallmark in diagnosis [8], mostly causing problems in episodic memory. Since the worldwide prevalence of AD is increasing due to higher life expectancy and reducing mortality rates [9], socio-economic, personal and emotional burden as well as primary and secondary costs for treatment and care rises [10]. Despite current breakthroughs in pharmaceutical therapy [11,12], alternative and complementary approaches and strategies in the treatment and maintenance of AD are urgently needed to reduce individual and caregiver burden and healthcare costs. Non-pharmacological, accessible and affordable therapies to maintain or even improve cognitive functions, ADL, Qol and general wellbeing are desirable. Home-based individualized and person-centered approaches within the family with no or only minor support by a caregiver constitute a column of possible strategies [13].

Computer-based cognitive learning and interventions, i.e., serious games, lately gained interest and have shown to be valuable in maintaining cognition during healthy aging and Mild Cognitive Impairment (MCI) [13]. Such technological interventions include, for example, personal computers (PCs), tablet-PCs/smartphones, gaming consoles, virtual, augmented or mixed reality. The advantages of computer-based interventions include a relative safety in usage and a lack of side effects [14].

Beneficial effects were observed for people with MCI in the cognitive domains memory, working memory, attention/ concentration/ processing speed, executive functions, language and psychosocial functioning [14]. Incipient beneficial effects in AD were reported for overall cognition and visuospatial skills [14], which however, were found for virtual reality and Nintendo Wii only. Hence, the evidence for efficacy of computerized cognitive training in AD appears to be weak.

The aim of the present randomized controlled trial (RCT) is to examine the effect of a tablet-based, multimodal, partly personally supervised, home-based training compared to voluntary, unsupervised cognitive paper-and-pencil exercises in people with mild to moderate AD over 6 months.

We hypothesize that supervised tablet-based multimodal training will maintain global cognitive skills and cognitive domains (such as memory, attention, executive functions), and is superior when compared to conventional unsupervised training. Also, we hypothesize that subcortical brain atrophy over 6 months is lower in the intervention compared to the control group. A comprehensive neuropsychological battery and structural brain examinations with MRI administered before and after training served to examine the efficacy of the intervention.

## Methods

### Design

The multimodAAL study is a two-arm, parallel group RCT with a longitudinal approach examining people with AD who were randomly assigned to either an intervention or a control group. The reporting was guided by the CONSORT Checklist [15] (S1 Checklist).

#### Impact of COVID-19 on the present study.

The study protocol was retrospectively registered on ClinicalTrials.gov (NCT04628702) on 12 November 2020, after recruitment had already begun on 18 February 2020. Unfortunately, registration prior to the start of recruitment was missed due to (administrative) challenges and the onset of the COVID-19 pandemic. The first COVID-19 cases in Austria were reported on 25 February 2020 [16], causing significant uncertainty and impacting the project.

Additionally, the COVID-19 pandemic led to a deviation in the intervention duration, which was shortened from 18 to 6 months due to the difficulties in predicting the future course of the pandemic, as well as the project timeline becoming tight as a result. In this context, the originally planned “training cafés” for the social component of the intervention (S1 and S2 Files) were not implemented; however, the frequency of the supervised training sessions was increased from bi-weekly to once a week, with appropriate safety measures in place.

Furthermore, the pandemic impacted the recruitment of this study. Potential participants declined due to concerns about weekly home visits or assessments at the hospital, fearing infection. Some refused safety measures, making their participation impossible. To counteract this, the research team sought support from a professional recruitment agency. However, the originally planned sample size of 220 participants could not be achieved, with only 25 participants randomized and 22 included in the final analysis.

Regarding the endpoint measures, no changes were made after the trial started. However, this paper focused only on the primary and secondary outcomes of cognition. Other secondary outcomes stated in the protocol will be published elsewhere.

### Participants

From 18 February 2020 to 30 November 2021 participants who were living at home were recruited (e.g., via the non-profit Austrian Red Cross Organization), their diagnoses verified or (if not yet happened) diagnosed with mild to moderate AD at the Medical University of Graz, Austria. Inclusion criteria were a confirmed diagnosis of AD, age over 40 years, stable dementia medication for at least 3 months, being fluent in German, sufficient physical, auditory and visual skills to perform a tablet-based multimodal training as well as an available informal caregiver that was willing to participate in the study alongside the participant. People that were participating in another clinical trial, with prior history of cancer, myocardial infarction, hepatitis, HIV, syphilis or current depressive episode as well as people with signs in magnetic resonance imaging (MRI) such as lacunar infarction, confluent lesions or other focal lesions were not included in the study.

**Fig 1:**
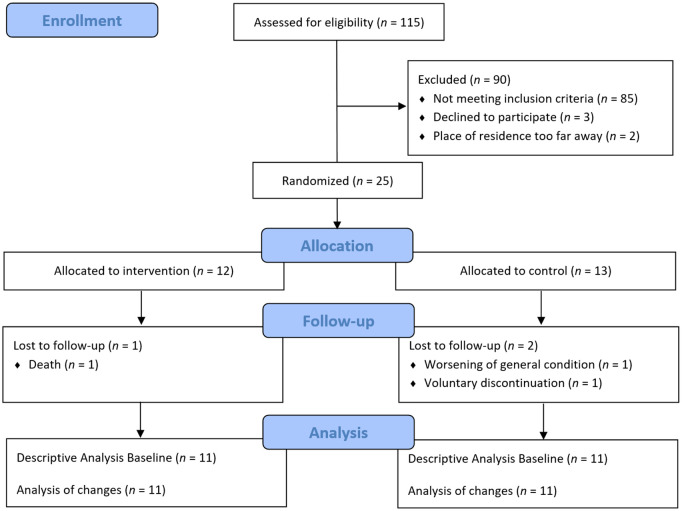
Flow diagram of participants through the study (Figure based on CONSORT-Statement).

A total of 115 individuals were assessed for eligibility, with 25 participants meeting the inclusion and exclusion criteria and subsequently being randomized (Fig 1). During the course of the study, three participants dropped out. As a result, 22 participants successfully completed the study by July 2022.

### Sample size calculation

G*Power was used to calculate the required sample size for the study design [17]. As the focus of this study was on comparing two groups (control group, intervention group) across two time points, a power analysis for a 2x2 analysis of covariance (ANCOVA) was performed. In order to detect a medium-sized effect (f = 0.25) with 80% power and a significance level of α = 0.05, 128 individuals (64 per group) are needed. A total sample size of 220 participants was determined to account for potential drop-outs due to factors such as study duration, health deterioration, hospitalization, or admission to a nursing home.

### Randomization and blinding

Group allocation was performed randomly using an Excel-based randomization generator that had been created and fixed by the study nurse prior to study initiation and could not be modified thereafter. The study nurse was the only person with access to this list.

Participant enrollment (i.e., screening for inclusion and exclusion criteria, study briefing, and obtaining informed consent) was conducted by a blinded neurologist who had no knowledge of the allocation sequence and no access to the randomization document. Following the signing of informed consent, participants were assigned to a group by the study nurse using the randomization list and received a pre-generated pseudonymized code that did not allow any conclusions to be drawn regarding group allocation. The order in which information about study participation was forwarded was based on the time point at which informed consent was provided.

Neurological and neuropsychological examiners were blinded to group assignment.

### Intervention

The participants of the intervention group received free access to a tablet-based multimodal training (i.e., cognitive and physical exercises) for six months. At the beginning of the intervention period, participants and their caregivers were trained in the interventions’ use, and for any concerns, a telephone hotline was available. During this period, the participants were encouraged to train as often as possible alone or with a caregiver. In addition, the participants had weekly visits from a trained employee of the Austrian Red Cross Organization for a joint supervised training session. The trainers also ensured that the participants performed the training appropriately and helped if questions concerning the implementation of the training had arisen.

**Fig 2:**
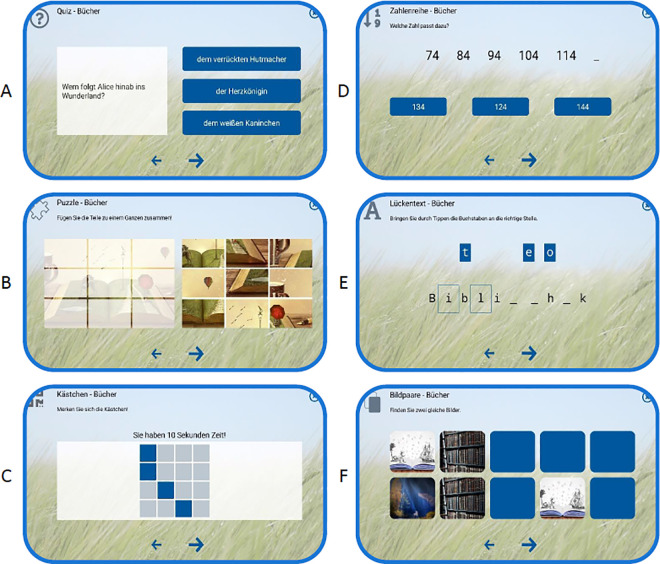
Examples of six cognitive exercise types (out of a total of 13) included in the tablet-based multimodal training (© Joanneum Research DIGITAL). (A) Quiz, (B) Puzzle, (C) Grid task, (D) Number sequence, (E) Fill-in-the-blank, (F) Matching pairs.

The multimodal training was developed by a multidisciplinary team from the fields of dementia, care and information technology. The methodology of the intervention is based on a holistic training according to five pillars: cognition, movement, perception, ADLs, games and creativity. Each training session had a specific theme (e.g., water, books, history, animals) and began with three video-based, light physical exercises. This was followed by cognitive exercises that included, for example, verbal and visual tasks, recognition tasks, calculations, and tasks targeting working memory and visual attention (Fig 2). The training consisted of four levels of difficulty, adjusted either by the trainers’ professional expertise or by the participants themselves. Participants were able to complete each task at their own pace [18–20]. For more information, please refer to the study protocol (S1 and S2 Files) and the qualitative evaluation of the intervention [18].

During the active study period, the control group received cognitive paper-and-pencil exercises (e.g., tasks targeting spatial orientation) for voluntary, unsupervised training. The materials were prepared in a way that allowed for independent use. Employees of the Austrian Red Cross Organization delivered these exercises to participants in the control group once per quarter during home visits or via postal service, if personal contact was not desired due to COVID-19. Instructions for completing the exercises were provided during these visits or via the telephone hotline if needed; however, the completed materials were not collected afterward. For ethical reasons, individuals in the control group were given free access to the same tablet-based multimodal training after the completion of the study.

### Neuropsychological assessments (primary outcome measures)

The comprehensive neuropsychological test battery was administered pre and post training and conducted by an experienced psychological rater included tests for selective cognitive domains such as working memory, memory, attention and executive functions as well as global cognition. The following cognitive assessments were administered: the Mini Mental Status Examination (MMSE [21]), the Montreal Cognitive Assessment (MoCA [22]), immediate and delayed visual pair recognition (Wechsler Memory Scale, Revised, WMS-R [23]), immediate and delayed verbal pair recognition (WMS-R [23]), digit span (WMS-R [23]), letter-digit substitution test within 90 seconds (LDST [24]), the verbal learning and memory test (VLMT [25]), verbal fluency for F-, A- and S-words, the categorical fluency test (animals), the trail making test A and B (TMT-A/B [26]), and the mean reaction time for cognitive flexibility of the computerized test battery to examine attention (Testbatterie zur Aufmerksamkeitsüberprüfung, TAP [27]).

### MRI data acquisition

Magnetic resonance imaging was performed on a 3T whole body scanner (Prisma; Siemens Healthcare, Erlangen, Germany) with a 12-channel head coil. For each participant, a high-resolution T1-weighted three-dimensional anatomical image with magnetization preparation (MPRAGE) and whole brain coverage (TR = 1900 ms, TE = 2.19 ms, inversion time = 900 ms, flip angle = 9°, isotropic resolution of 1 mm) was acquired. For the identification of white matter hyperintensities, an axial T2-weighted FLAIR sequence (TR = 10,000 ms, TE = 69 ms, inversion time = 2500 ms, number of slices = 40, slice thickness = 3 mm, in-plane resolution = 0.86 mm × 0.86 mm) was used.

### Statistical analyses

The statistical analysis was performed using the software SPSS (version 27, SPSS Inc., Chicago, IL). The level of statistical significance was considered to be a two-sided p-value of <0.05. The parameters of the follow-up visit were approximated for six months after the baseline visit to cope with deviations from the follow up period.

Two-way repeated measures analyses of covariance (ANCOVA) were performed to assess group differences in the cognitive parameters and the MRI data between participants of the intervention and control group after six months while controlling for prior disease duration and years of education. The assumption of homogeneous variances between the groups was tested by using Levene’s tests at each assessment (time point). Box’s M test was used to test whether the observed covariance matrices of the dependent variables are equal across groups. Assumptions for ANCOVA (1. homogeneity of regression slopes, 2. no significant group differences in the covariates, i.e., disease duration and years of education) were tested prior to the analyses. Bonferroni correction was applied for pairwise comparisons.

For MRI data, image pre-processing and subcortical segmentation was performed using FreeSurfer versions 4.3 through to 5.3 [28], with the “-recon-all” pipeline and default settings. This pipeline performs automated bias field correction, spatial normalization, skull stripping, and segments brain tissue into cortical gray/white matter, as well as into several non-cortical tissues. This resulted in volume estimates for the following seven bilaterally paired structures (secondary outcome parameters): nucleus accumbens, amygdala, caudate nucleus, globus pallidus, hippocampus, putamen, and thalamus. Analyses were corrected for estimated intracranial volume.

To calculate the training count and training duration, a training session was defined as the collection of exercises grouped under a theme (e.g., water) in the training app. The number of completed trainings represented the quantity of sessions where at least 60% of the exercises were completed, with the result of each exercise being marked as “passed” or “failed”. The duration of trainings was measured from the start of the first exercise to the end of the last exercise. A filter was applied to exclude exercises that took longer than 600 seconds to complete.

### Ethics approval and consent to participate

The trial protocol (S1 and S2 Files) was reviewed and approved by the ethics committee of the Medical University of Graz, Austria, on 5 February 2020 (31–556 ex 18/19), in accordance with the Declaration of Helsinki. All subjects provided written informed consent to participate in the study.

## Results

Overall, twenty-two individuals with a confirmed diagnosis of AD were included in the analyses. Eleven individuals were assigned to the intervention group and eleven individuals to the control group (Fig 1, Table 1). Participants of the intervention group trained 4.12 (2.86 SD) times per week on average with a mean duration of 22.71 minutes (10.12 SD).

**Table 1:** Demographics and characteristics of the intervention and the control group.

	Intervention group	Control group
**Age**	76.57 (9.15)	74.89 (6.99)
**Sex (female/ male)**	8/ 3	8/ 3
**Education (years)**	11.45 (3.96)	10.91 (3.67)
**Disease duration (years)**	1.63 (1.69)	1.57 (1.78)

Shown are mean and standard deviation.

**Fig 3:**
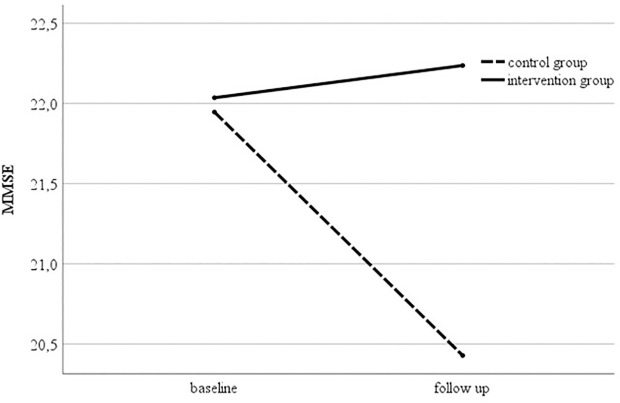
MMSE mean scores for intervention and control group at baseline and six-month follow-up. Presented group means are corrected for disease duration and years of education. Post-hoc test revealed a significant decline in the control group and a maintenance of the MMSE score in the intervention group. MMSE = Mini-Mental State Examination.

Repeated measures ANCOVA for the outcome global cognitive function (MMSE) revealed a significant interaction term (F1, 14 = 5.083, p = .041) for intervention and group with a large effect size (partial eta^2^ = 0.266, Table 2, Fig 3) indicating an effect of the tablet-based multimodal training over time. The Bonferroni-corrected post-test revealed a significant decline of global cognition in the control group (p = .014), while the performance of the intervention group remained stable over six months (p = .712). Other cognitive domains or cerebral volumetric analyses did not reveal significant effects (Table 2 and 3). Due to incomplete neuropsychological examinations at either baseline or follow up, the numbers used for the calculation varies.

**Table 2:** Results for the mean cognitive differences between intervention and control group after six months.

	Intervention group		Control group		F-statistics, *P*^a^	Partial eta^2^
	baseline	follow up	baseline	follow up		
**MMSE**						
	22.11 3.59 *n* = 9	22.24 3.71	21.89 2.76 *n* = 9	20.45 2.94	*F*_1, 14_ = 5.083, *p* = .041^*^	0.266
**MoCA**						
	17.22 3.90 *n* = 9	16.53 4.60	16.44 3.47 *n* = 9	14.18 3.39	*F*_1, 14_ = 3.463, *p* = .084	0.198
**Visual pairs, immediate**						
	4.25 2.55 *n* = 8	4.64 2.64	4.00 2.50 *n* = 9	4.72 1.85	*F*_1, 13_ = .103, *p* = .754	0.008
**Visual pairs, delayed**						
	1.75 1.91 *n* = 8	1.97 1.83	1.67 1.23 *n* = 9	1.62 1.04	*F*_1, 13_ = .166, *p* = .690	0.013
**Verbal pairs, immediate**						
	8.33 4.92 *n* = 9	8.07 4.59	9.29 1.89 *n* = 7	8.88 1.97	*F*_1, 12_ = .541, *p* = .476	0.043
**Verbal pairs, delayed**						
	2.50 3.02 *n* = 6	2.86 2.67	2.71 1.98 *n* = 7	2.96 1.59	*F*_1, 9_ = .041, *p* = .844	0.005
**Digit span total**						
	11.56 4.56 *n* = 9	10.71 3.69	9.56 2.46 *n* = 9	9.38 2.12	*F*_1, 14_ = .591, *p* = .455	0.041
**LDST, 90sec**						
	21.00 10.93 *n* = 8	20.88 11.70	25.86 6.82 *n* = 7	24.54 7.78	*F*_1, 11_ = .465, *p* = .510	0.041
**VLMT**						
	24.50 6.39 *n* = 8	24.90 6.62	25.75 7.09 *n* = 8	24.90 6.62	*F*_1, 12_ = 2.232, *p* = .161	0.157
**Word fluency F**						
	8.89 4.14 *n* = 9	8.97 3.27	8.00 4.95 *n* = 9	7.48 3.23	*F*_1, 14_ = 0.587, *p* = .456	0.040
**Word fluency A**						
	7.33 3.64 *n* = 9	6.89 3.31	5.88 3.23 *n* = 8	5.89 2.98	*F*_1, 13_ = 0.392, *p* = .542	0.029
**Word fluency S**						
	9.00 3.00 *n* = 9	9.14 2.19	8.33 3.08 *n* = 9	6.79 3.18	*F*_1, 14_ = 2.367, *p* = .146	0.145
**Categorial fluency**						
	11.33 4.00 *n* = 9	11.29 3.57	8.44 3.28 *n* = 9	8.77 3.30	*F*_1, 14_ = .001, *p* = .972	<0.001
**TMT-A (sec)**						
	109.67 54.02 *n* = 9	110.92 60.17	73.33 43.46 n = 9	81.73 43.53	*F*_1, 14_ = .867, *p* = .368	0.058
**TMT-B (sec)**						
	240.11 76.31 *n* = 9	246.85 72.05	268.44 40.69 *n* = 9	261.68 48.26	*F*_1, 14_ = 1.892, *p* = .191	0.119
**TAP (msec)**						
	985 413 *n* = 8	1602 1761	1328 1356 *n* = 9	1317 1269	*F*_1, 13_ = 2.262, *p* = .156	0.148

Mean, standard deviation and number of subjects for which ANCOVA was calculated with the respective neurocognitive tests. Results (interaction terms) of the two-way repeated measures ANOCVAs for the mean difference in cognitive parameters between individuals of the intervention and control group after six months. The results are controlled for the covariates disease duration and years of education.

MMSE = Mini-Mental State Examination total score, MoCA = Montreal Cognitive Assessment total score, LDST = letter digit substitution task within 90 seconds, VLMT = sum of score of run 1–5 in the verbal learning and memory test, TMT-A = trail making test A (seconds), TMT-B = trail making test B (seconds), TAP = mean time (milliseconds) for the cognitive flexibility task from the computerized test battery.

* p < .05.

**Table 3:** Results of subcortical grey matter differences between groups at baseline and six months.

	intervention group		control group		*F*_1, 12_ ^a^	*P* ^b^
	baseline	follow up	baseline	follow up		
	mean (SE)	mean (SE)	mean (SE)	mean (SE)		
**nuc.**^c^ **accumbens (l)**^d^	2,17E-04 (1,49E-05)	2,15E-04 (1,17E-05)	2,27E-04 (1,79E-05)	2,26E-04 (1,41E-05)	.815	.466
**nuc. accumbens (r)** ^e^	2,59E-04 (2,10E-05)	2,59E-04 (2,16E-05)	2,64E-04 (2,53E-05)	2,60E-04 (2,61E-05)	.995	.398
**amygdala (l)**	7,08E-04 (4,52E-05)	6,97E-04 (4,71E-05)	7,06E-04 (5,46E-05)	6,91E-04 (5,69E-05)	.012	.988
**amygdala (r)**	7,75E-04 (4,49E-05)	7,59E-04 (4,20E-05)	7,98E-04 (5,42E-05)	7,74E-04 (5,08E-05)	.119	.889
**caudate (l)**	2,02E-03 (6,99E-05)	2,00E-03 (6,90E-05)	2,14E-03 (8,45E-05)	2,07E-03 (8,34E-05)	2.418	.131
**caudate (r)**	2,18E-03 (7,94E-05)	2,07E-03 (6,91E-05)	2,25E-03 (9,60E-05)	2,17E-03 (8,35E-05)	.085	.919
**hippocampus (l)**	1,90E-03 (1,23E-04)	1,87E-03 (1,18E-04)	1,98E-03 (1,49E-04)	1,93E-03 (1,42E-04)	.539	.597
**hippocampus (r)**	1,86E-03 (1,14E-04)	1,80E-03 (1,17E-04)	1,92E-03 (1,38E-04)	1,84E-03 (1,42E-04)	.555	.588
**pallidum (l)**	1,25E-03 (4,96E-05)	1,23E-03 (4,18E-05)	1,28E-03 (5,99E-05)	1,27E-03 (5,05E-05)	.053	.984
**pallidum (r)**	1,20E-03 (5,33E-05)	1,20E-03 (4,39E-05)	1,21E-03 (6,44E-05)	1,21E-03 (5,31E-05)	.028	.973
**putamen (l)**	2,64E-03 (1,19E-04)	2,60E-03 (1,05E-04)	2,69E-03 (1,44E-04)	2,69E-03 (1,27E-04)	1.299	.309
**putamen (r)**	2,56E-03 (1,13E-04)	2,52E-03 (9,12E-05)	2,65E-03 (1,36E-04)	2,59E-03 (1,10E-04)	.177	.840
**thalamus (l)**	3,87E-03 (1,12E-04)	3,89E-03 (9,49E-05)	4,04E-03 (1,35E-04)	3,95E-03 (1,15E-04)	2.841	.098
**thalamus (r)**	3,88E-03 (1,32E-04)	3,82E-03 (1,43E-04)	3,99E-03 (1,59E-04)	3,92E-03 (1,73E-04)	.288	.755

Mean, standard error and F-Test (interaction terms) of a two-way repeated measures ANCOVAs for the difference in subcortical grey matter (corrected for estimated intracranial volume) between individuals of the intervention (n = 8) and control group (n = 7) at baseline and after six months. The results are controlled for the covariates disease duration and years of education.

^a^ F-statistics.

^b^ p-value.

^c^ nucleus.

^d^ left.

^e^ right.

## Discussion

In this single-blinded RCT, we investigated the effect of a six-month, tablet-based, (un)supervised multimodal training in people with mild to moderate AD. The training included cognitive and physical exercises and was compared to voluntary, unsupervised cognitive paper-and-pencil exercises provided to an age- and sex-matched control group with comparable disease duration. A comprehensive neuropsychological assessment and MRI before and after training was used to reveal efficacy.

We found the tablet-based training to be beneficial for global cognitive functioning (i.e., the MMSE) in the sense that within the intervention group, cognitive function remained stable, whilst the cognitive function within the control group deteriorated over the six-month examination period. However, no such effects were found for the cognitive domains attention, executive function, figural memory or verbal fluency. Also, no effects of a reduced atrophy rate on selected cerebral areas were found for the intervention group when compared to the control group. Although, trends or positive effects in subjective cognitive decline [13] and MCI [29] were reported, we did not find such results for people with mild to moderate AD. Hence, to the best of our knowledge, this is the first RCT using computerized cognitive training in community-dwelling people with mild to moderate AD reporting a positive effect on global cognition [13].

The systematic review of Zuschnegg et al. [13] included six RCTs of community-dwelling people with dementia, who individually performed computerized cognitive trainings, involving a total of 273 participants (sample size range 11−115 subjects) with a mean age of 66.3 years. However, meta-analyses revealed no beneficial effects considering global cognition, memory function, working memory, attention/concentration/processing speed, executive function and language when comparing the intervention to the control group. Also, meta-analyses three months post intervention did not show significant results [13]. Technologies used in dementia studies of this systematic review [13] were PCs [30–33], tablets [34] and a non-immersive virtual reality technology [35]. In contrast, another recent systematic review by Chan et al. [36] identified a significant effect regarding verbal episodic memory in their meta-analysis (standardized mean difference (SMD) 0.64, 95% confidence interval (CI) [0.02–1.27]) among people with dementia (n = 371), which encompassed nine RCTs. The meta-analysis revealed high heterogeneity (I^2^ = 87%). A subgroup analysis, excluding two studies, no longer shows a significant effect (SMD 0.23, 95% CI [−0.02–0.49]) (heterogeneity was not reported by the authors [36]). In one [37] of these two studies, the intervention included not only computerized training but also a substantial amount of cognitive-linguistic exercises with pen and paper, which may explain its outlier status.

The comparisons of our results with other studies are difficult since studies differ in study designs considering type, duration and number of sessions, length of intervention, stage of cognitive decline (e.g., subjective cognitive decliner, subjects being at risk of dementia, mild cognitive impairment, mild to moderate or moderate to severe forms of dementia), comorbidities, outcome measures, sample size, settings (hospital, daycare, residential care centers, at home), appropriate control groups, an agreement of what clinical significance means even if there are statistically significant results and also, whether positive effects are persistent over longer periods post intervention and transferability to real life [13,36]. A homogenization between studies is necessary. Also, future studies should examine the effect of the combination of new available pharmacological drugs with computer-based cognitive interventions.

The results of non-computerized training studies in dementia are also contradictory, which might be due to a great variation in the level of dementia observable between the studies. The staging ranges from mild to moderate [38,39] or from moderate to severe forms of AD [40,41]. Two independent studies in mild to moderate AD and moderate to severe AD reported beneficial effects. In the former an improvement of global cognition measured with the MMSE was reported [38]. In the latter, a maintenance of cognitive functions, and a stabilization of cognitive functions ten months beyond the end of the therapy, were found [41]. Amieva et al. [40] further examined potential effects of training in groups (cognitive training and reminiscence therapy) and individualized training and could not find beneficial effects of group sessions of cognitive training and reminiscence therapy over a follow up period of two years in a large sample of 650 people with AD. However, an individualized cognitive rehabilitation program did show positive effects with lower functional disability and a six-month delay in institutionalization [40]. The results of Amieva et al. [40] suggest that individualized cognitive interventions should be considered prior to cognitive-oriented group therapies.

Further studies using cognitive training methods without technical support, such as stimulation therapy, reality orientation and skills training, found similar results for persons being at risk of cognitive decline [42] and also for people with MCI [14,43–51]. In a 2-year multi-domain intervention RCT in persons at risk of cognitive decline, Ngandu et al [42] reported beneficial effects for the combination of cognitive training, diet, exercise and vascular risk monitoring.

In our study, we used a combination of cognitive and light physical training to mentally and physically activate participants. We included the physical training as supplement to cognitive training since it is well described in the past that benefits of exercise and physical activity prolong cognitive functions [52]. Hence, we consider the combination of cognitive skills training and physical activity using a tablet as in our study as a beneficial concomitant side-effect free treatment for global cognitive functions during mild to moderate AD. Inspired by these results, we speculate that already minor clinical effects in cognitive maintenance in people with AD can prolong independency and therefore delay institutionalization with direct effects on personal life, societal and economic costs.

With (emerging) technologies, more standardized, side-effect free and completely individualized multimodal training programs become available. Prior studies using PCs, tablets, virtual and augmented or mixed reality revealed heterogeneous results during the course of cognitive decline from subjective cognitive complainers to severe forms of AD [13]. For example, Bahar-Fuchs et al. [43] trained a small sample of people with MCI using a computerized cognitive training and found an improvement in composite measures of memory, learning, and global cognitive functions at follow up. Also, non-cognitive outcome measures improved. Authors conclude that home-based computerized cognitive training with adaptive difficulty and personal tailoring appears superior to more generic training. Our study also included exercises that could be individually tailored to the person with AD (e.g., training topic, difficulty level, duration). However, upcoming advancements in artificial intelligence (AI), such as Large Language Models (LLMs) (e.g., GPT-4o) hold promise for further individualization of cognitive training. LLM-based chatbots could enhance personalization of such training by processing information from various sources, such as user preferences and performance data, to generate personalized training tasks related to the user’s own life experiences and provide individually appropriate feedback [53]. Furthermore, emerging technologies such as virtual reality hold potential for cognitive training in individual preferred environments (e.g., beach), with eye tracking and digital signals used to measure cognitive performance and the stress level [54].

### Limitations and strengths

One limitation of this study is the small sample size. This was primarily due to the onset of the COVID-19 pandemic, which significantly hindered recruitment, as many people were discouraged from maintaining continuous social contact with study personnel. Nevertheless, a significant positive effect on global cognition was observed for the intervention group. The small sample size, however, may have contributed to the lack of significant results in other study outcomes, suggesting that additional or stronger beneficial effects could have been observed with a larger, adequately powered sample size [55,56].

Second, there was no beneficial effect on the subcortical volumes, i.e., we did not find differences in the atrophy between the intervention and the control group during the examination period. This insignificance is most likely due to the short examination period. Since atrophy rates in AD within one year have been reported to be approximately four percent when compared to healthy controls [8], the effect potentially induced by a cognitive training within six months is too small to be detected.

Third, we acknowledge the impact that the shortened intervention duration (6 vs. 18 months) may have had on the observed effect sizes. Longer intervention periods are generally associated with more robust cognitive benefits, particularly in progressive neurodegenerative conditions such as AD. Reducing the training duration to 6 months may have limited the opportunity for cumulative effects to emerge, potentially leading to smaller effect sizes. Importantly, even within this reduced timeframe, we were able to detect meaningful changes, suggesting that cognitive stabilization approaches may show measurable effects even in the short term. However, it is noteworthy that the actual intervention period still exceeded the median duration of comparable cognitive training studies, which is typically around 12 weeks [57]. Nevertheless, it remains essential to conduct long-term studies to assess the durability of intervention effects in people with AD.

Fourth, the difference in supervision between the intervention and control group may have had a potential confounding effect. While participants in the intervention group received weekly supervised training sessions during home visits, the control group was provided with paper-and-pencil cognitive materials delivered twice over the course of six months. To mitigate potential bias arising from differing contacts, we ensured that both groups had access to a telephone hotline. However, it is possible that the increased contact time and interaction with the trainer during the supervised training sessions positively influenced motivation, self-esteem, and well-being, or introduced other confounding social benefits that were not controlled for in this study. Future studies should take this issue into account.

Fifth, the small sample size and the single-site setting may limit the generalizability of our findings. The study was conducted in only one of Austria’s nine federal states (i.e., Styria), and some of the exercises included in the multimodal app may be culturally specific. Although the training is available in other languages, these versions have not yet been validated [20]. Given the limited sample size and the specific demographic and contextual setting, further research is needed to explore the effects of such a multimodal, tablet-based training in more diverse and global populations.

Finally, the single-site setting – the home environment – should also be considered a valuable strength of our study, as most studies involving people with dementia living at home are still conducted in institutional settings or laboratory environments [13]. In this regard, qualitative findings from the usability evaluation, conducted as an integral part of this study [18], revealed that the motivation of informal caregivers may play an important role in supporting adherence to such training for their loved ones with dementia. Since this aspect of caregiver motivation and involvement was not addressed in the current RCT, future research should explore and give more attention to the role of informal caregivers in such interventions conducted at home.

## Conclusion

Tablet-based multimodal training, including aspects of cognitive and physical activation, had positive effects on global cognition of people with mild to moderate AD over a six-month training period. However, it lacks measurable transferability to other cognitive domains such as memory, attention or executive functions or brain structure. This aspect requires further investigation, as does the transferability of the positive effect observed within the intervention group to real-life activities.

To advance research in this field, high-quality longitudinal studies with adequately powered sample sizes, as well as  standardized intervention protocols, are needed. With regard to the intervention, considerations should be given to the availability of emerging technologies (e.g., virtual reality), increasing personalization (e.g., through AI), combining cognitive exercises with physical activities, and integrating newly available pharmacological treatments.

Furthermore, the role of informal caregivers in supporting adherence should be considered when implementing such training at home, with particular attention to avoiding any additional caregiving burden.

## Supporting information

S1 Checklist CONSORT Checklist for randomized controlled trials. (PDF)

S1 File Trial protocol approved by the ethics committee [Original in German]. (PDF)

S2 File Trial protocol approved by the ethics committee [Translated into English]. (PDF)
