# Supplementary material for: The effect of tablet-based multimodal training on cognitive functioning in Alzheimer’s disease: A randomized controlled trial
Source: PLoS One. 2025 Aug 13;20(8):e0329931. doi: 10.1371/journal.pone.0329931 (PMC12349711; doi:10.1371/journal.pone.0329931)
Supplement: S1 File — (PDF) [file pone.0329931.s002.pdf]

According to the journal's guidelines, copyrighted institutional logos have been removed from the published version.

Study Protocol

Playful Multimodal Intervention, Monitoring and Decision Support for Activation of People with Alzheimer's Dementia

Short Title of the Project: MultimodAAL

According to the journal's guidelines, copyrighted images have been removed from the published version.

Translation

Ass. Prof. PD Mag. Dr. Marisa Koini[1], Sen. Scient. Dr. Sandra Schüssler[2], Univ. Prof. Dr. Reinhold Schmidt[1]

Medizinische Universität Graz

University Clinic for Neurology, Clinical Department of Neurogeriatrics[1] and Institute of Nursing Science[2]

Table of Contents

Additional Information on the Project 4
1. Summary 4
2. Background 5
3. Objectives..7
4. Methode 7
4.1. Design 7
4.2. Setting and Sample 8
4.2.1. Setting 8
4.2.2. Sample 8
4.2.2.1. Inclusion and Exclusion Criteria 9
4.2.2.2. Recruitment 12
4.2.2.3. Randomization and Blinding 12
4.3. Intervention 13
4.3.1. Study Procedure 13
4.3.2. Training 14
4.3.2.1. Data Collection Personnel...14
4.3.2.2. Training of Participants 15
4.3.3. Hotline and Contact Persons 15
4.4. Data Collection Methods 15
4.4.1. Table Overview of Data Collection Methods 16
4.4.1.1. Description of Data Collection Methods...23
4.5. Statistics...38
4.6. Ethical Considerations 38
4.6.1. Informed Consent 38
4.6.2. Data Protection 39
4.6.2.1. Data Protection for Questionnaires and Interviews.........................39
4.6.2.2. Data Proctection and Privacy for “DaheimAktiv” 39
4.6.2.3. Data Protection for Fitness Tracker 40
4.7. Benefits/Risks...40
4.7.1. Benefits 40

Translation

4.7.2. Risks 41
References 42
Appendix 1 Data Collection Instruments 49

Translation

Additional Information on the Project

Follow-up Project of AktivDaheim, funded by FFG; Ethics Application EK No: 1505/2016

1. Summary

Background: The prevalence of dementia is increasing worldwide, leading to a growing demand for healthcare services, which is facing an increasing care gap due to the decreasing number of available caregivers. Consequently, many new technologies, such as tablet-PC training programs, have been developed with the potential to support caregivers and individuals with dementia in promoting care independence and stabilizing the course of the disease. Previous studies that include computer-based training for individuals with dementia typically focus only on the cognitive and psychological effects of primarily cognitive training programs. However, international literature recommends applying multimodal training programs (e.g., including physical and social components in addition to cognitive aspects).

Objectives: The primary objective is to investigate the effectiveness of the multimodal tablet-PC training program "DaheimAktiv" on cognitive abilities in individuals with early-stage Alzheimer's dementia. Secondary objectives include assessing the usability and acceptance of the tablet, structural and functional changes in the brain, quality of life, mobility, motivation, lifestyle factors, hobbies, stress, emotions, care dependence, (instrumental) activities of daily living, medication changes, endogenous/biological factors, behavioral problems, caregiver burden, activity level, and arm strength, as well as evaluating potential depressive symptoms.

Methods: A mixed-method study will be conducted (1.5 years). The quantitative part is a randomized controlled trial (RCT). The qualitative part includes supplementary interviews (individual interviews, focus groups). The intervention group (110 individuals with Alzheimer's dementia) will receive a multimodal (physical, cognitive, and social components) tablet-PC training program, while the control group (110 individuals with Alzheimer's dementia) will receive a standard intervention. Data will be collected using tablet-PCs, eye tracking, fitness tracking, performance tests, MRI, blood samples, questionnaires, and interviews will be conducted. Additionally, supplementary questionnaires will be administered to relatives (n=220), dementia trainers (n=5), and

Translation

professional caregivers (n=5). All n=220 patients will undergo a detailed neurological and neuropsychological examination before the start of the training (=intervention group) or during the waiting period (=control group) and after the 1.5-year training/waiting interval. Furthermore, an MRI will be performed at both time points. Data analysis will be conducted quantitatively (descriptive statistics, inferential statistics) and qualitatively (content analysis).

This study promotes the development of computer-based tablet-PC training programs for the target group of Alzheimer's dementia in clinical practice.

2. Background

The demographic shift is leading to an increase in older individuals with chronic conditions, such as dementia (NIH & WHO, 2011; Robert Koch Institute, 2015), and consequently an increased demand for healthcare services. However, this is countered by a growing care gap due to the decreasing number of available caregivers (Robert Koch Institute, 2015).

People in the early stages of dementia are primarily cared for at home (OECD, 2015), but as dementia progresses and care needs increase—due to care dependence (e.g., mobility, cognitive learning ability) and care issues (e.g., falls, malnutrition)—professional care becomes increasingly necessary and may end in institutional care if home care can no longer be ensured (ADI, 2013; OECD, 2015; Braunseis et al., 2012). One of the main tasks of caregiving (and primary healthcare) is to promote the independence of individuals with dementia to counteract a rapidly progressive disease course with increasing care dependence (Schüssler, 2015). Computer-based technologies, such as tablet PC training, could support caregivers. These technologies not only have the potential to promote the (care) independence of older individuals but also to enhance their well-being (Smarr et al., 2012; WHO, 2007), which could ultimately stabilize the disease course.

So far, new technologies such as tablet PCs have been used to a limited extent in practical caregiving. The study by Nordheim et al. (2015) tested tablet PCs with individuals with

Translation

severe dementia. The use showed positive results regarding communication, motivation, and behavior of residents. The target group was individuals in nursing homes, so conclusions about people in home settings can only be drawn with limitations. The study by Lim et al. (2012) examined the usability of tablet PCs for individuals with mild dementia in a home environment and found that half of the participants were able to use the tablet independently with minimal support, despite 95% having no prior experience with tablets. The review by Joddrell et al. (2016) showed that 45 included studies demonstrated that people with dementia manage well with touchscreen technologies. International research highlights the need to include individuals with dementia in studies (Wu et al., 2014; Span et al., 2013; Mao et al., 2015) to obtain their feedback and recommendations for the further development of new technologies (Boman et al., 2014). Generally, people with dementia desire technologies that provide cognitive support, promote communication, social interaction, and life activities (e.g., mobility) (Lauriks et al., 2007; Wang et al., 2016).

Previous studies that include computer-based training for individuals with dementia using tablet PCs mostly focus solely on the cognitive and psychological effects of primarily cognitive training programs (Djabelkhir et al., 2017; Ehret et al., 2015; Fasilis et al., 2018; Garcia-Casal et al., 2017; Hitch et al., 2017; Klimova & Maresova, 2017; Nordheim et al., 2015). However, the work by Schneider & Yvon (2013) and Chalfont, Milligan, and Simpson (2018, a systematic review) suggests that interventions are more effective when conducted multimodally (i.e., also including physical and social components alongside cognitive aspects). The present study includes a multimodal PC training program for individuals with dementia, which examines effects on cognitive, psychological, physical, and ADL (activities of daily living/care dependence) levels, thereby supporting the development of computer-based training programs for the dementia target group.

Translation

3. Objectives

Primary Objective:

Examine the effectiveness of the multimodal tablet-PC training program "DaheimAktiv" on cognitive abilities in individuals with early-stage Alzheimer's dementia.

Secondary Objectives:

- Quantitative: Assess structural and functional changes in the brain (e.g., global and local brain volume, white matter changes), quality of life, mobility, motivation, lifestyle factors, stress, care dependence, (instrumental) activities of daily living, changes in medication, endogenous/biological factors (i.e., the significance of DNA variants, DNA methylation processes, or changes in gene expression related to dementia), behavioral problems, caregiver burden, activity level, and arm strength, as well as evaluate potential depressive symptoms and utilize the collected data for the development of a decision support system.
- Quantitative: Investigate the usability and acceptance regarding the use of the training program.
- Qualitative: Conduct supplementary interviews (focus groups and individual interviews) on usability.

Translation

4. Methode

4.1. Design

The study is a "Mixed-Methods Study" with an "Embedded Design." The design of the quantitative part is a "Randomized Controlled Trial" (RCT). The qualitative part involves supplementary interviews (individual interviews and focus groups) with content analysis. The RCT will be registered in the database ClinicalTrials.gov (https://www.clinicaltrials.gov/ct2/home). This is an exploratory study aimed at

investigating new relationships between tablet-based training and possible outcomes (e.g., cognition, motivation, quality of life).

4.2. Setting and Sample

4.2.1. Setting

The study will be conducted in Styria. Participants with dementia will come from home settings, assisted living, and nursing home environments.

4.2.2. Sample

The study aims to include 220 patients with Alzheimer's dementia (as per NINCDS-ADRDA criteria). Additionally, their primary relatives/informal caregivers (n=220), 5 professional caregivers and 5 M.A.S. (Alzheimer Syndrome) trainers (individuals conducting stage-specific training for people with dementia in individual and group sessions across various care settings) will be included.

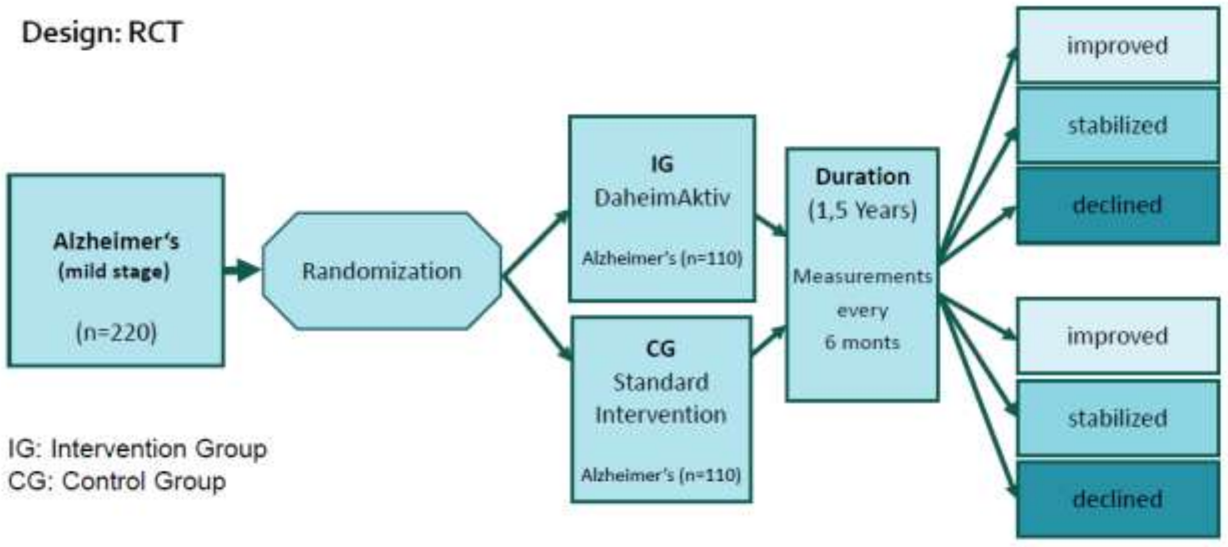

Figure 1: Overview of the Randomized Controlled Trial (RCT)

4.2.2.1. Inclusion and Exclusion Criteria

<u>People/Person with Dementia (PwD)</u>

Inclusion Criteria:

- A diagnosis of possible or probable dementia according to NINCDS-ADRDA criteria (McKhann et al., 2011).
- The person with dementia (PwD) is over 40 years old at the baseline assessment.
- The PwD speaks and understands German and possesses sufficient physical, auditory, and visual abilities to undergo a neuropsychological examination and participate in tablet-based training.
- The PwD has been receiving stable medication therapy for at least 3 months prior to the baseline visit.
- Memantine therapy must have started at least 3 months before the baseline visit.
- A relative/informal caregiver accompanies the PwD to the neurological examination at the University Clinic for Neurology at T0 and T3.
- If the PwD has paid 24-hour care, a relative must be recruited as a participant/informal caregiver.
- The PwD lives with or without a relative/informal caregiver at home (if alone, relatives/informal caregivers should live nearby).
- The PwD has professional (e.g., home nursing care) and/or informal care (e.g., by relatives) or receives no care.
- The PwD is capable of giving consent or a relative with representation authority or legal guardian provides consent to participate in the study.
- The PwD is not taking antipsychotics or antidepressants, or has been stabilized on these medications for at least 14 days before the study start.

Exclusion Criteria

- The PwD has participated in a clinical study in the 3 months before the baseline visit.
- The PwD is expected to be unable to complete the study.
- Any of the following signs on the brain MRI:

Translation

  - ✓ Infarct near major vessels
  - ✓ More than one lacunar infarct, defined as a focal lesion in the CSF signal intensity with a diameter of less than 1.5 cm in all spatial directions.
  - ✓ A lacunar infarct in a strategically important region such as the thalamus, hippocampus in both hemispheres, or the caudate nucleus.
  - ✓ Confluent lesions of the deep white matter (Fazekas Score 3).
  - ✓ Other focal lesions that could be responsible for the patient's cognitive status (e.g., infections, space-occupying lesions, normal pressure hydrocephalus).
  - ✓ The PwD has undergone or has a planned operation with general anesthesia within the last 3 months before the baseline visit or during the study period.
- The PwD has had or is currently undergoing clinically significant immunomodulatory treatment or will receive such treatment in the future.
- The PwD has a cancer diagnosis (last treatment ≤ 5 years before the baseline visit).
- The PwD has had a myocardial infarction within the last 2 years before the baseline visit.
- The PwD has hepatitis B, C, HIV, or syphilis.
- The PwD has an active infectious disease.
- The PwD suffers from a systemic disease that is likely to cause rapid deterioration or compromise safety:
  - ✓ Uncontrolled heart failure (NYHA>3)
  - ✓ BMI>40
  - ✓ Poorly controlled diabetes
  - ✓ Severe renal insufficiency
  - ✓ Chronic liver disease
  - ✓ Other clinically relevant systemic diseases.
- The PwD suffers from hypothyroidism. Persons with treated hypothyroidism may participate if they have had stable therapy for more than 3 months before the baseline visit.

Translation

- The PwD suffers from a psychiatric illness, such as schizophrenia, psychotic disorders, or bipolar disorder.
    - ✓ The PwD currently has a depressive episode (Geriatric Depression Scale GDS ≥6 at baseline visit) or major depression within the last 2 years.
- The PwD has a metabolic or toxic encephalopathy or dementia due to general medical conditions.

Relatives/Informal Caregivers

Inclusion Criteria:

- Adult relatives (women and men) of the participating PwD
- Live with the PwD in the same household or not
- The PwD receives or does not receive professional care
- The relative provides no care or provides care themselves
- Speaks and understands German
- Capable of giving consent

Professional Caregivers

Inclusion Criteria:

- Adult women and men
- Qualified nurses or nursing assistants
- Speaks and understands German
- Capable of giving consent

M.A.S. (Morbus Alzheimer Syndrom)/Dementia Trainers

Inclusion Criteria

- Adult women and men
- Trained M.A.S. trainers
- Conduct training for the participating PwD
- Speaks and understands German
- Capable of giving consent

Translation

4.2.2.2. Recruitment

Participants will be recruited through the staff of the non-profit organization Sozialverein Deutschlandsberg (SVDL) and the Austrian Red Cross (project partners) via telephone, flyers, advertisements, websites, events, social media, and personal contact. The sampling method used is "convenience sampling." Additionally, information flyers will be distributed at the SVDL and in medical practices.

4.2.2.3. Randomization and Blinding

<u>Randomization of Participants with Dementia</u>

A randomization plan will be prepared in advance. Allocation to the Intervention Group (IG) and Control Group (CG) will be determined beforehand using a random list created in Matlab. Among participants 1-110, 70% will be allocated to the IG and 30% to the CG. For participants 111-220, 30% will be allocated to the IG and 70% to the CG. This ensures that, in the case of potential enrollment difficulties, a sufficient number of participants receive the intervention. It should be explicitly noted that the aim is to provide the intervention rather than achieving equal distribution between the IG and CG. At the Department of Neurology, only the Study Nurse will know whether a patient is in the IG or CG. The investigators (neurologist, neuropsychologist) will not know the group allocation.

<u>Blinding</u>

Blinding of the staff of SVDL and the Austrian Red Cross (ÖRK) involved in the study is not possible because the intervention is apparent.

Translation

4.3. Intervention

„DaheimAktiv“:

According to the journal's guidelines, copyrighted images have been removed from the published version.

Figure 2: "DaheimAktiv"

"DaheimAktiv" is an app on a tablet PC that includes a serious game, which was further developed and tested for people with dementia in a prior project (AktivDaheim, funded by FFG; Ethics Application EK No: 1505/2016). "DaheimAktiv" provides multimodal training in a playful manner through cognitive and physical exercises. These exercises can be individually tailored to the person (e.g., content, difficulty level adjusted to the stage of dementia, sequence, duration). "DaheimAktiv" always starts with physical exercises, including music, which are precisely explained via text on the tablet and video. This is followed by cognitive exercises, knowledge questions (quizzes), seek-and-find images, puzzles, memory, fill-in-the-blanks, calculations, listening tasks, and songs. "DaheimAktiv" can be used both at home and in care facilities (e.g., nursing homes).

4.3.1. Study Procedure

In the first step, a prescreening of potential participants is conducted using a shortened list of inclusion and exclusion criteria through the SVDL and the Austrian Red Cross. Potential patients are then subjected to a clinical-neurological examination (including vital signs), a blood draw, and a cognitive assessment (performance tests and questionnaires) at the Medical University of Graz, Department of Neurology, at Time Point T0-a (see **Table 1**). Based on the clinical-neurological examination and cognitive assessment, it will be determined whether the participant needs a structural and functional magnetic resonance imaging (MRI) (T0-b) to medically confirm an early-stage Alzheimer's diagnosis. Simultaneously, the relatives/informal caregivers will be interviewed regarding caregiving and personal data. For participants with Alzheimer's dementia who are included in the study, additional data (see **Table 1**) will be collected at 6 months (T1) and 12 months (T2). After the 1.5-year tablet training, a clinical-

Translation

neurological examination, including blood draw, a cognitive assessment, including questionnaires, and a structural and functional MRI (T3) will be performed again.

<u>Intervention Group (IG)</u>

The IG undergoes a 1.5-year tablet PC-based training program for cognitive and physical skills using DaheimAktiv. This training is conducted biweekly by a M.A.S. trainer, nurse, or pedagogically trained person from the Sozialverein Deutschlandsberg (SVDL) or the Austrian Red Cross (ÖRK) together with the person with dementia (PwD). The tablet remains with the PwD at home, and participants are encouraged to continue training even without the presence of a trainer (with or without a family member). All training sessions of the PwD on the tablet are recorded. DaheimAktiv is a multimodal intervention procedure with cognitive and physical exercises. In addition to the biweekly training sessions with trainers and the independent training, "Training Cafés" will be held in small groups to promote motivation and increase social integration. This means that several PwD will train together.

<u>Control Group (CG)</u>

In the control group, any medical interventions initiated outside or independently of the study will continue (standard intervention). Otherwise, the PwD in the CG are subject to the same study conditions as those in the intervention group. PwD in the CG will receive a free one-year license for DaheimAktiv after the evaluation phase is completed.

4.3.2. Training

4.3.2.1. Data Collection Personnel

<u>Questionnaires, Interviews</u>

To minimize inter-rater variability, the data collection personnel from the Sozialverein Deutschlandsberg and the Red Cross will be trained by staff from the Medical University of Graz (Departments Neurology and Nursing Science) on the questionnaires and interviews (individual interviews, focus groups).

Translation

Intervention Training

The Sozialverein Deutschlandsberg and the Red Cross will train their dementia trainers for the intervention application. The training will last approximately 4 hours.

4.3.2.2. Training of Participants

All participants will receive training for the application of the intervention from the Sozialverein Deutschlandsberg.

4.3.3. Hotline and Contact Persons

During the study, the Sozialverein Deutschlandsberg will set up a hotline for participants to address questions and issues during the study period. Regular hours for this service will be provided.

4.4. Data Collection Methods

Table 1 summarizes the data collection methods used in the study. Following this, a brief description will be provided.

Translation

4.4.1. Table Overview of Data Collection Methods

Table 1: Overview of Data Collection Methods of the Study

Data	Data Collection Methods	Target group (Self- or Proxyassessment) or autonom	Duration	Frequency<br>T0-a, T0-b=Baseline<br>T1=after 6 months<br>T2=after 12 months<br>T3=after 18 months<br>ongoing
**Basic characteristics of the sample, such as:**<br>• Age<br>• Gender<br>• Education<br>• Previous occupations<br>• Initial diagnosis of dementia<br>• Place of residence<br>• Living situation<br>• ICD-10 diagnoses<br>• Legal care level<br>• Medications	Questionnaire	**PwD:**<br>Self and/or Proxy (e.g., relative, (family) doctor, caregiver)	Depending on the availability of the informants	T0-a<br><br>T3
**Lifestyle factors**	Fantastic Lifestyle Checklist	**PwD:**<br>Self with assistance	7 min.	T0, T1, T2, T3
	Tablet PC („DaheimAktiv“)<br>• [SOA] Social Activities: "Pick-a-social-activity" • (Stress: see “Pick-a-mood”)<br>• [STI] Mood: “Pick-a-mood”	**PwD:**<br>Self with assistance	SOA: 20 Sec.<br>STI: 20 Sec.<br>ADI: 2 Min.<br>BLU: 10 Min.<br>ERN: 20 Sec.<br>RAU: 20 Sec.	At least every 2 weeks (with MAS Trainer – only in IG)

	• [ADI] Obesity: GUI BMI data with current weight, height (default values)<br>• [BLU] Blood Pressure: GUI[1] input for systolic/diastolic current values<br>• [ERN] Nutrition: "pick-a-diet" • [RAU] Smoking: "pick-a-smoking-behavior"<br>• [BEW] Exercise: "pick-a-mobility"/measured via fitness tracker<br>• [SCH] Sleep: "pick-a-sleep"		BEW: 20 Sec.<br>SCH: 20 Sec.<br><br>Total: 3 minutes (without BLU) to 13 minutes (with BLU)	
**Sample characteristics such as:**<br>• Age<br>• Gender<br>• Education<br>• Residence<br>• ICD-10 diagnoses<br>• Care situation and level of care<br>• Employment status	Questionnaire	**Informal caregivers:** Self	7 min.	T0-a
**Sample characteristics such as:**<br>• Age<br>• Gender<br>• Education<br>• Place of residence<br>• Professional experience	Questionnaire	**Professional caregivers:** Self	3 min.	T0-a

[1] Graphical User Interface: Interactive Input on Tablet PC (with DaheimAktiv Software)

Translation

• Occupation				
**Basic characteristics of the sample such as:**<br>• Age<br>• Gender<br>• Education<br>• Place of residence<br>• Professional experience<br>• (Previous) occupation	Questionnaire	**M.A.S. Trainers:**<br>Self	3 min.	T0
**Clinical neurological examination including vital signs**		**PwD:**<br>Self	45 min	T0-a, T3
**Cognitive Status**	MMSE (Mini Mental State Examination)	**PwD:**<br>Self	15 min.	T0-a, T3
	Neuropsychological Test Battery<br><br>• Wechsler Memory Scale (WMS-III-R) - Visual and verbal paired recognition<br>• Verbal Learning and Memory Test (VLMT) - Immediate and delayed recall<br>• Wechsler Memory Scale, Revised - Digit span<br>• Regensburg Word Fluency Test (RWT)<br>• Wechsler Memory Scale - Delayed recall<br>• Letter Digit Substitution Test (LDST)	**PwD:**<br>Self	60 min	T0-a, T3

Translation

	• Trail Making Test A/B			
	TAP (Testbatterie zur Aufmerksamkeitsprüfung) [Attention Test Batterie]	**PwD:**<br>Self	5 min	T0-a, T3
**Motivation**	AES (Die Apathy Evaluation Scale)	**PwD:**<br>Self (with assistance) and<br>proxy by an informal caregiver	5 min.	T0, T3<br><br>T0, T1, T2, T3
	FAM (Questionnaire for Assessing Current Motivation in Learning and Performance Situations)	**PwD:**<br>Self (with assistance)	7 min.	every 6 months before tablet training
**Quality of Life**	DEMQOL (Dementia Quality of Life)	**Proxy by an informal caregiver**	10 min.	T0, T3
	WHOQOL 100 (World Health Organization Quality of Life Scale)	**Informal Caregiver:**<br>Self	30 min.	T0-a, T3
**Care dependency (ADL)**	PAS [CDS] (Care Dependency Scale)	**Proxy by an informal caregiver**	5 min.	T0, T3
**Activity Level (ADL)**	Pool Activity Level (PAL) Checklist	**Proxy by an informal caregiver**	5 min	T0, T3
**Mobility**	Activity Analysis via Tablet PC	**PwD:**<br>Autonomous Sensors	During „DaheimAktiv“	Ongoing (during physical excercises)
	Fitness tracker or MOVE	**PwD:**<br>Autonomous mobil Sensors	Throughout the day (from morning until evening, not overnight)	Throughout the entire study duration

Translation

	TUG (Timed UP and GO Test)	**PwD:**<br>Proxy by data collector	5 min.	T0-a,T3
**Global Cognition**	MoCA (Montreal Cognitive Assessment)	**PwD:**<br>Proxy by data collector	15 Min.	T0, T1, T2, T3
**(Instrumental) activities of daily living**	Disability Assessment for Dementia Scale (DAD)	**PwD:**<br>Proxy by an informal caregiver	15 min	T0-a, T3
**Executive functions/cognitive control**	• MIRA (Mobile Instrumental Review of Attention): Analysis of gaze behavior with an eye tracker on/in the tablet PC using specific interactive ("serious game") exercises:<br>• Trail Making Test A, B<br>• Anti-Sakkadentest<br>• Go/Nogo Test<br>• Spot-the-difference<br><br>Test of Attentional Performance (TAP): Sustained attention, executive functions	**PwD:**<br>Autonomous through Eye-Tracker	Ongoing during tablet-PC training (voluntary)	Every 2 weeks on the tablet with the MAS trainer ("mandatory exercise")
**Measurement of arm strength**	Arm strength	**PwD:**<br>Self	2 min.	T0-a, T3
**Psychiatric factors**	Neuropsychiatric Inventory (NPI)	**Proxy by an informal caregiver**	10	T0-a, T3
**Depression or depressive symptoms**	GDS (The Geriatric Depression Scale)	**PwD:**<br>Self	5 min	T0-a, T3

Translation

**Affektive Stimmung**	Affective Slider	**PwD:**<br>Self (on the Tablet)	6 min	Every 14 days before tablet training
**Usability**	TUI (Technology Usage Inventory)	**PwD, Informal and professional caregivers, Trainers:**<br>Self	10 minutes for people with dementia, otherwise 5 minutes	T0, T3
**Usability, Akzeptanz**	Individual interviews	**PwD:**<br>by data collector	Ca. 20-50 min.	1x<br>(after the intervention)
	Focus groups	**Informal and professional caregivers, trainers**	Approximately 1½ - 2 hours per target group	1x<br>(after the intervention)
**Caregiver burden, perceived stress**	ZBI (Zarit Burden Interview)	**Informal caregivers:**<br>Self	10 min.	T0, T3
	PSS-10 (Perceived Stress Scale)	**Angehöriger:**<br>Self	5 min.	T0, T3
**MRT of the brain**		**PwD:**<br>Self	45 min.	T0-b, T3
**Blood sampling**		**PwD:**<br>Self	5 min	T0-a, T3

Translation

According to the table, there are three measurement time points at the University Clinic for Neurology/Neuroradiology. At the first time point, the person with dementia undergoes a clinical neurological examination, a neuropsychological examination, and a blood draw. The total examination duration will be about two and a half hours. If the person with dementia still meets the strict inclusion and exclusion criteria after this examination day, they will be invited to a further appointment. At the second examination appointment, an MRI will be performed. The MRI takes approximately 45 minutes. At the end of the tablet-based training, after 1.5 years, all examinations will be repeated (duration: about three hours).

Between the different medical and psychological examinations, breaks will be provided, and a break will be given upon the patient's request. Additionally, time will be allocated for a meal. To relieve the patient and their informal caregivers on the day of the examinations at the University Clinic for Neurology, questionnaires that do not require neuropsychological training from the examiner will be filled out by the patients together with their informal caregivers or a staff member from the SVDL or ÖRK at home without time pressure. Clinical tests will be conducted by psychologists. The questionnaires will be provided at the beginning, after 6, 12, and 18 months, with each filling time taking less than half an hour for both the patient and the informal caregiver.

Due to the potential high burden on patients and informal caregivers during their visits to the University Clinic for Neurology, as well as the resource-intensive nature of the examinations and data management, an evaluation of the study design's feasibility will be conducted after the first 10 enrolled patients. Both patients and their informal caregivers will be surveyed after each examination day regarding the duration and burden of individual examinations (neurological and neuropsychological assessments, blood draw, and MRI), as well as the overall examination process. If a majority of patients and informal caregivers report undue burden, the study protocol will be adjusted accordingly. Additionally, the burden on the personnel at the University Clinic for Neurology will be continuously evaluated, and the study protocol will be adjusted as needed. The current study plan involves three physicians, one technician, five psychologists, and one study coordinator, each assigned specific tasks.

Translation

4.4.1.1. Description of Data Collection Methods

<u>**Data Collection via Tablet PC**</u>

Collection and Analysis of Activity from Video Features on Tablet PC

To estimate the activation of individuals during physical exercise and to infer the motivational status of persons with dementia based on this, movement analysis will be conducted using a video camera embedded in the Tablet PC. The Tablet PC should be placed upright on a table, with the camera directed towards the person performing the exercise.

At the start of each exercise session, a video with a 20-second recording duration will be captured by the webcam and stored in the data repository. Only the user's personal code will be saved with the video. The camera generates an output stream with 640x480 pixels at 30 frames per second (fps) or 2560x1920 pixels at 1 fps.

Upon return of the Tablet PC, the videos will be transferred by the JOANNEUM RESEARCH (JR) study team to a PC protected by a firewall at JR. An image analysis software, based on the method described by Cao et al. (2017), will extract and encode the skeleton data of the person in the video (see **Figure 2**). The video will then be deleted by automated software, ensuring that no JR staff member can view the appearance of the users. Only the skeleton data will be stored to extract kinetic energy and emotions following the methodology of Piano et al. (2014). Finally, only features such as emotion and energy for specific observation periods will be stored per anonymized user, while all other data will be deleted.

Translation

According to the journal's guidelines, copyrighted images have been removed from the published version.

(a) (b)

Figure 3 Exemplary Video Analysis of a User's Movements. The algorithm by Cao et al. (2017) enables the extraction of skeleton data from video images. Only the skeleton data is stored, associated with an anonymized code for the test person, while the video data is deleted immediately after analysis. The activation of the users is derived from the skeleton data.

Analysis of Gaze Behavior on the Tablet PC

To analyze gaze behavior, the study employs software designed to analyze webcam data on the Tablet PC. The video data is processed in real-time using a static eye-tracker on the Tablet PC. The video data itself is not stored; instead, after an initial calibration of gaze geometry, only metadata, such as fixations and saccades of the participants during tablet use, is automatically and continuously recorded. Training sessions for the antisaccade task and the "Visual Paired Comparison" test are prepared so that participants are unaware of the gaze behavior analysis. This approach ensures that participants focus on the task at hand while allowing the evaluation of executive functions (inhibitory functionality: Diamond et al., 2013).

Gaze Behavior Analysis Metrics:

<u>Antisaccade Task</u>

Participants view alternating blocks of prosaccade and antisaccade tasks on a 10.5" Tablet PC screen in a game-like format (24 trials per block, as per Kaufmann et al., 2010). Each trial presents a central stimulus for 2000 ms, followed by a peripheral star stimulus for 1000 ms on either the left or right side of the screen. Each trial in the antisaccade task is classified as follows:

Translation

- "No Error": When the participant's gaze does not fixate on the stimulus (i.e., the gaze is not within 2° of the line of sight directed at the stimulus at any measurement point).
- "Error": When the gaze line is within the threshold angle at least once during the trial.
- "Corrected Error": When the gaze line returns outside the threshold angle after an "Error" before the trial ends.
- "Uncorrected Error": When the gaze line does not return outside the threshold angle after an "Error" before the trial ends.

<u>Visual Paired Comparison Task</u>

In this task, a reference pattern and a comparison pattern are repeatedly presented to the participant, who must determine how the two patterns differ. The trial is terminated by the participant using a stop button upon reaching a decision. The following metrics are assessed according to Lagun et al., 2011:

- Novelty Preference (NP): The percentage of gaze duration on the comparison pattern relative to the total gaze duration.
- Fixation Duration (FD): Total fixation duration until the task is terminated.
- Re-fixations (RF): Average depth of re-fixation during the task. Re-fixation occurs within a 2° deviation between current and previous fixation. Re-fixation depth is the number of saccades between the current fixation and the previously visited fixation.
- Saccade Orientation (SO): Percentage of "vertical saccade angles." A vertical saccade angle is a saccade angle within the interval (90° ± 7°). A saccade angle is the angle between the current and previous saccade vectors.
- Reaction Time (RT): The time interval between the start of a comparison task and the participant's termination of the task.
- Error Rate (ER): Errors in classification (incorrect details classified as errors).

Collection and Analysis of Cognitive Functions Using Tablet PC

In the multimodal interactive training "DaheimAktiv," all interactions and events on the tablet PC are recorded, anonymized with a personal code, and then stored in a central database for further data analysis. The results enable a quantitative comparison of the

Translation

training games and related performance metrics with the psychological questionnaires to identify suitable correlates.

The MIRA (Mobile Instrumental Review of Attention) software module allows for the automated tracking of the user's eye movements via the webcam. The following modules are designed for data collection and subsequent analysis:

- Component "Trail Making Test": Random patterns are generated in the style of the "Trail Making Test" A and B. The user can engage in a playful manner (as a video game), and the data are recorded.
- Component "Go/Nogo": In the style of a go/nogo test, stimuli are presented sequentially, and the user will interact with corresponding artifacts, which will be available for further analysis.
- Component "TAP-Concentration": Following the "Test of Attentional Performance," concentration is measured during an abstract task. The data can be analyzed via the central database.

Lifestyle Factors Using Tablet PC

The tablet PC will also continuously, yet playfully, collect lifestyle factors to enhance Alzheimer's dementia management (see "Pick-A-Mood," Desmet et al., 2016). These data collection methods are designed to contribute to "serious game" effects that further encourage their use. Users will be rewarded with playful, non-competitively superficial success tableaux, such as a garden where each contribution to data collection grows a flower or tree, creating beautiful botanical displays. This anticipated user engagement can drive further participation. The planned data collection components are:

- "Social Activities": Daily social contacts are recorded by selecting from various buttons, each representing a specific level of social interaction for the day: "no person," "1 person on a chair," "2 people," and so on. → Selection through "Pick-A-Social State."
- Pictorial Representation of Various Mood States: Through "Pick-A-Mood" (see Appendix 2; Desmet et al., 2016).
- Input of Current Weight and Height: With a constant reference from the biographical data section, the current BMI value is graphically and playfully displayed.

Translation

- Input of Systolic and Diastolic Blood Pressure Values: If available, with special "reward values" in the serious game.
- Pictorial Representation of Various Daily “Dietary Forms”.
- Pictorial Representation of Different “Sleep Patterns”.
- Pictorial Representation of Various “Smoking Behaviors”.

Data Collection Methods – Performance Tests

MMSE

The cognitive status will be assessed using the German version of the MMSE (Mini-Mental State Examination) (Folstein et al., 1975). The MMSE is the most widely used cognitive test globally (Folstein et al., 1975; Mahlberg & Gutzmann, 2005; Hensel et al., 2007; Hensel et al., 2009), and its psychometric properties have been extensively tested (Rösler et al., 2003; Mahlberg & Gutzmann, 2005; Hensel et al., 2007; Schramm et al., 2002; Kahle-Wrobleski et al., 2007). The MMSE allows for screening cognitive impairment across large populations (Folstein et al., 2010) and enables the determination of the degree of cognitive impairment, ranging from no impairment to severe cognitive impairment (Rösler et al., 2003).

Neuropsychological Test Battery (Harrison et al., 2007)

- **WMS III-R Figural Pair Recognition Including Delayed Recall:** In this subtest of the Wechsler Memory Scale III-R (Härting et al., 2000), the participant is required to remember 6 line drawings, each paired with a specific color. Subsequently, the line drawings are presented alone, and the participant must indicate the corresponding colors in the test booklet. A maximum of 18 points can be achieved for this task. During the delayed recall, the figures are shown again at a later time, and the participant must indicate the corresponding color, with a maximum of 6 points available.
- **WMS III-R Verbal Pair Recognition Including Delayed Recall:** This test is the verbal counterpart to the previous subtest. The participant is read 8 pairs of words,

Translation

and they must recall the second word when the first word of each pair is later presented. During the delayed recall, the words are read again at a later time, and the participant must name the corresponding word. The maximum score is 24 points for the immediate recall and 8 points for the delayed recall.

- **Verbal Learning and Memory Test (VLMT):** The VLMT is a test of serial list learning with subsequent distraction, recall after distraction and a 30-minute delay, as well as a recognition phase. The test material consists of two word lists, each containing 15 semantically independent words, and a recognition list that includes the 30 words from the two lists plus 20 additional semantically or phonematically similar distractor words. The VLMT assesses various parameters of declarative verbal memory, such as span, learning performance, long-term encoding and recall, and recognition performance. The VLMT corresponds to the English version of the Auditory Verbal Learning Test.
- **WMS III-R Digit Span:** This subtest (Härting et al., 2000) consists of two types of tasks: digit span forward and digit span backward. In the digit span forward task, the participant is presented with sequences of digits of increasing length, which they must immediately repeat. The sequences increase in length by one digit per trial, up to eight digits in a row. Each trial includes two sequences (e.g., two sequences of 3 digits, two sequences of 4 digits, and so on). In the digit span backward task, the participant is again presented with sequences of digits of increasing length, but this time they must repeat the sequences in reverse order. The sequences also increase in length by one digit per trial, up to seven digits in a row, with each trial including two sequences. If the participant fails to correctly repeat either of the two sequences in a trial of the digit span forward task, that trial ends and proceeds with the digit span backward task. If the participant also fails to correctly repeat either of the two sequences in a trial of the digit span backward task, the subtest is terminated. Each correctly repeated sequence scores one point. The maximum score for this task is 24 points.
- **RWT:** The RWT (Regensburg Word Fluency Test; Aschenbrenner et al., 2001) is a diagnostic tool for assessing verbal fluency, where participants must generate verbal responses over a period of one or two minutes. The test includes subtests for both formal-lexical and categorical word fluency. Normative values are provided

Translation

for both one-minute and two-minute intervals. The interrater reliability for all subtests is r = .99. Test-retest reliability over three weeks varies between rtt = .72 and rtt = .89 for different subtests. The test has been validated in various neurological and psychiatric patient samples (e.g., patients with brain tumors, patients after cerebral infarcts, patients with long-term alcohol dependence, patients with major depression). The results from the validation samples described in the test manual demonstrate the broad applicability of the test and the high sensitivity of its subtests in a striking manner.

- **LDST (Letter Digit Substitution Test):** The LDST is a timed substitution test (Speed Test, van der Elst, 2006). Substitution tests are sensitive to cerebral dysfunctions and are nonspecific, capturing a broad range of different processes. These processes include the integration of complex neuropsychological functions such as visual scanning, mental flexibility, attention, psychomotor speed, and information processing speed.

TAP (Testbatterie zur Aufmerksamkeitsprüfung)
The TAP is a computer-based psychological testing system designed for attention diagnostics. With the Test Battery for Attention Assessment (TAP), various aspects of attention in both children and adults can be evaluated (Zimmermann & Fimm; Version 2.3.1).

MoCA (Montreal Cognitive Assessment)
The MoCA is a tested screening tool for cognitive status. It can also measure changes in overall cognitive decline (Freitas et al., 2012; Costa et al., 2014). The MoCA assesses attention and concentration, executive functions, memory, language, visuoconstructive skills, conceptual thinking, calculation, and orientation. The instrument has been translated into 46 languages and is used in over 100 countries (Nasreddine, 2018).

NPI (Neuropsychiatric Inventory) (Cummings, 1997)
The Neuropsychiatric Inventory, developed by Cummings et al. in the USA in 1994, is used to assess neuropsychiatric symptoms. The NPI consists of twelve subscales that capture ten behavioral symptoms and two neurovegetative states. The basis of this

Translation

assessment tool is a structured interview with an informant. Each subscale starts with a question about whether a specific symptom is present or not. If the informant answers "Yes," the symptom is then assessed in more detail using a scale. If the symptom is not present, the interview moves to the next symptom complex. Caregivers rate the frequency using a four-point scale and the severity using a three-point scale.

Imaging Methods

MAGNETIC RESONANCE IMAGING (MRI)

In this study, MRI is used to confirm a diagnosis of Alzheimer’s disease dementia. To minimize sample heterogeneity, it is essential to distinguish from other conditions and ensure that other causes, such as vascular lesions, tumors, normal pressure hydrocephalus, or metabolic encephalopathies, are not responsible for the deficits. MRI is mandatory for diagnosis.

The following sequences, totaling approximately 45 minutes, are performed:

- 3D-EPI with Multiecho: for R2* Mapping and QSM
- 3D-T2
- 3D-T1 Multiecho MPRAGE: for improved segmentations
- 3D FLAIR
- Diffusion 1.5 mm isotropic with Multiband and multiple b-values
- T2-Sequence for Microbleeds*
- Resting State (functional MRI)

BIOBANK

The biobank is established to investigate endogenous biological factors in Alzheimer's dementia in relation to the clinical course of the disease. The biobank consists of the collection of DNA, RNA, serum, and plasma samples, which are collected at baseline and after 1.5 years. This setup allows for both cross-sectional and longitudinal studies to examine the significance of DNA variants, DNA methylation processes, and changes in gene expression concerning dementia. Blood samples are taken at the Neurological University Clinic, anonymized, and assigned a study number before being sent to the

Translation

clinic's laboratory for further processing. DNA and RNA extraction, as well as the archiving and management of the biobank, are carried out at the Neurological University Clinic Graz according to the regulations of §68 of the Genetic Engineering Act. The samples are aliquoted, stored at -80°C, and made available for future projects.
The following quantities are collected:
For routine laboratory tests: 1 x 8ml serum tube, 1 x 8ml lithium-heparin tube, 2 x 3ml EDTA tubes, 1 x 3.5ml coagulation sodium citrate tube. For genetic investigations: 3 x 6ml EDTA tubes, 1 x 8ml serum tube, 1 x RNA sample

GENETIC ANALYSES

For the study, ApoE genotyping is required. The ApoE4 genotype is associated with faster progression of Alzheimer's disease, so it is crucial to ensure that there is no imbalance in the frequency of ApoE4 carriers between the training group and the comparison group.
Given that DNA extraction is necessary, we will take this opportunity to ask patients for consent to use their DNA for potential future scientific projects. It is understood that each of these future projects will require a separate ethical review.
All samples will be anonymized, labeled with a code rather than names, and processed. The samples can only be linked to the name of the donor at the University Clinic for Neurology. Should the results of genetic analyses or phenotype data be used in scientific collaborations, it will be ensured that the donor cannot be identified by the collaborating centers. If international collaborations are pursued, these will be specifically submitted to the ethics committee as described above.
At the Neurology Clinic, the following risk genes for Alzheimer's disease are currently being investigated as part of a Next-Generation Sequencing panel: ASNA1, ATP13A2, ATP1A3, ATP6AP2, C19orf12, CHCHD2, COMT, DCTN1, DNAJC13, DNAJC6, EIF4G1, FBXO7, FMR1, FTL, GBA, GCH1, GRN, HTRA2, LRRK2, MAPT, PANK2, PARK2, PARK7, PDE8B, PDE10A, PINK1, PLA2G6, PODXL, POLG, PRKAR1B, PRKRA, PTEN, RAB29, RAB39B, SLC30A10, SLC6A3, SNCA, SPG11, SPR, SYNJ1, TAF1, TENM4, TH, VPS13C, VPS35, ZFYVE26.
Such investigations are possible but depend on appropriate funding.

AES (Apathy Evaluation Scale)

Apathy can be understood as a loss of motivation, which is reflected on the levels of cognition, (observable) behavior, and emotion/affect. The internal consistency of the German translation is comparable to the original (Cronbach's $\alpha = 0.86$ in the original vs. $\alpha = 0.92$ in the translation). It shows good test-retest reliability and interrater reliability. Results regarding construct validity indicated that the scale is significantly correlated with the apathy scale of the NPI (Lueken et al., 2006).

DEMQOL (Dementia Quality of Life)

The instrument aims to assess the health-related quality of life of individuals with mild to moderate dementia. It consists of two interview-based versions: "DEMQOL-Self" for self-assessment of quality of life by individuals with dementia and "DEMQOL-Proxy" for proxy assessment of quality of life by caregivers (Bowling et al., 2015; Smith et al., 2005). It measures the frequency of experiences with certain emotions and aspects of memory function in daily life over the past week (Berwig et al., 2011). Both versions of the instrument have been tested for psychometric properties (Berwig et al., 2009; Berwig et al., 2011).

FAM

The "Fragebogen zur Erfassung aktueller Motivation in Lern- und Leistungssituationen" (FAM) [Questionnaire for Assessing Current Motivation in Learning and Performance Situations] was developed by Rheinberg et al. (2001) and is a questionnaire that uses 18 items to assess four components of current motivation in (experimental) learning and performance situations: fear of failure, likelihood of success, interest, and challenge. Both the German and American versions of the questionnaire have satisfactory consistencies (6 samples, N=944). Various experiments provide validity evidence that the pre-assessed motivational components are related to subsequent learning behavior and performance. According to Lewin (1946), it is assumed that behavioral tendencies result from the interaction between person and situational factors. In motivational psychology, such person factors are known as motives. These are considered enduring, highly generalized traits of a person that predispose them to prefer certain classes of incentives. Situational factors are the motivationally specific stimuli and the satisfaction opportunities that the

Translation

given situation can offer. If these stimuli align with the person's motivational structure, the current motivation with its guiding and energizing behavioral consequences results. It is this current motivation – not the motives themselves – that has a direct impact on behavior (Rheinberg et al., 2001).

Fantastic Lifestyle Checklist

The Fantastic Lifestyle Checklist was developed by Wilson et al. (1984) and is a validated instrument designed to assess lifestyle factors of individuals. The instrument consists of 25 questions across the following 9 areas: 1) Family and Friends, 2) Physical Activity, 3) Nutrition, 4) Tabacco and Toxic Substances, 5) Alcohol Consumption, 6) Sleep, Seatbelt Use, Stress, Safe Sex, 7) Behavioral Patterns, 8) Insight and 9) Career.

Fitness Tracker for Mobility Data Collection and Analysis

A fitness tracker is used to collect and analyze mobility data. The data collected provides information on activities (e.g., step count) and motivation to increase personal movement range. The log data from the fitness tracker can only be accessed by the participants through the manufacturer's fitness tracker app. No data is transferred to the consortium's systems.

Using a fitness tracker allows for activity monitoring and encourages participation in exercise sessions aimed at expanding the individual's personal movement range.

GDS (The Geriatric Depression Scale)

The Geriatric Depression Scale measures depressive disorders in older adults and is one of the most commonly used tools, also applicable to individuals with dementia (Sheehan et al. 2012). The scale has been tested for psychometric properties (Allgaier et al. 2011, Gauggel et al. 1999, Sheehan et al. 2012).

Interviews (Individual Interviews and Focus Groups)

These interviews focus on in-depth questions related to the quantitative TUI questionnaire. Individual interviews will be conducted with individuals with dementia, and focus groups will be held with other target groups (relatives, caregivers, and M.A.S. trainers) with a minimum of 3-4 participants per group. The interviews will be conducted

Translation

by experienced and trained staff (e.g., psychologists, educators) from the Sozialverein Deutschlandsberg (SVDL) and MUG Nursing Science. They will take place in a quiet setting either at home or at the SVDL, and will be recorded using smartphones or tablets. The recordings will then be transferred from the SVDL to the Institute for Nursing Science via the secure server of Joanneum Research for transcription and analysis.

PAS [CDS] (Care Dependency Scale)

The Care Dependency Scale (CDS) is used to assess care dependency with its German version (Dijkstra et al. 1996, Lohrmann 2003). The PAS consists of 15 items that evaluate physical and psychosocial aspects using a 5-point Likert scale (from completely dependent to independent). Scores can range from 15 (completely dependent) to 75 (completely independent) (Lohrmann et al. 2003). The instrument has been well-tested for psychometric properties (Dijkstra 1998, Lohrmann 2003).

PSS-10 (Perceived Stress Scale)

The Perceived Stress Scale (PSS), developed by Cohen, Kamarck, and Mermelstein (Cohen et al. 1983), is an established questionnaire for self-assessing perceived stress. It uses a 5-point Likert scale to measure the extent to which life has been experienced as unpredictable, uncontrollable, and overwhelming over the past month (0 = "never", 1 = "almost never", 2 = "sometimes", 3 = "fairly often", 4 = "very often") (Cohen et al. 1983, Klein et al. 2016). Higher scores indicate a higher level of perceived stress (Cohen et al. 1983, Klein et al. 2016). While the original scale consists of 14 items (PSS-14), the shorter version (PSS-10) is recommended for clinical research due to improved psychometric properties (Lee 2012). The German version of the PSS-10 also shows good internal consistency (Cronbach's $\alpha = 0.84$) (Klein et al. 2016)

TUG (Timed UP and GO Test)

Procedure for Assessing Body Balance/Mobility and Fall Risk During a Daily Movement Task. The person sits comfortably in a chair with armrests, positioned 3 meters from a defined target. Upon instruction, the person should stand up, pause briefly in front of the chair, walk to the defined spot 3 meters away, turn around without touching anything, walk back to the chair, turn around again, and sit back down in the chair (Podsiadlo et

Translation

al. 1991). The psychometric properties of this procedure are well-established. The interrater reliability is ICC = .99 (3 raters, n = 22), and the validity is supported by a correlation of r = -.81 with the Timed "Up and Go" versus the Berg Balance Scale (IQPR 2012; Bossers et al. 2012). This test is recommended for individuals with dementia (Bossers et al. 2012).

TMT A/B (Trail Making Test A/B)

The Trail Making Test is a neuropsychological test that assesses visual attention and cognitive flexibility. In this task, 25 points must be connected. There are two versions available: Version A, where only numbers need to be connected (1, 2, 3, 4, ...), and Version B, where numbers and letters must be connected alternately (1, A, 2, B, 3, C, ...). The goal is to complete the test as quickly as possible, with the measured time serving as the result.

Affective Slider

The assessment of momentary affective states ("emotions") is crucial for capturing the affective component in relation to performance data using the tablet PC ("Multimodal Activation" app, MMA). For self-assessment, the "Affective Slider" (AFSL; Betella & Verschure, 2016) is used. This tool requires only a single touch per scale on the pressure-sensitive tablet PC. Users are presented with a scale for evaluating current mood ("pleasure"; good-bad) and another scale for assessing arousal ("arousal"; calm-excited). These two dimensions allow for the representation of all fundamental emotions according to Russell's bipolar space model (Russell et al., 1999).

The AFSL is activated each time the user presses the "MIRA" (Mobile Instrumental Review of Attention) button on the home screen of the MMA app to access the associated attention and cognitive game components. The simultaneous date and time query prevents multiple activations of the AFSL, thereby avoiding saturation of user motivation.

In the analysis of study data, emotion data will be correlated with performance data from interactions with other components of the MMA app. This will facilitate deriving insights into the functionality of affective states in the context of cognitive performance in the daily lives of individuals with dementia.

Translation

TUI (Technology Usage Inventory)

The Technology Usage Inventory (TUI) is used to assess technology-specific and psychological factors that contribute to the actual use of a technology. It consists of 30 items divided across 8 scales. These include curiosity, anxiety, interest, user-friendliness, immersion, usefulness, skepticism, and accessibility. Additionally, the tool includes the "Intention to Use" (ITU) scale. The internal consistencies (Cronbach's alpha) of the eight scales are generally rated as good, ranging from $\alpha = .70$ to $\alpha = .89$. A factor analysis revealed an 8-factor structure for the TUI. Furthermore, there is evidence for the psychophysiological validation of individual TUI scales (Kothgassner et al. 2013).

WHOQOL-100 (World Health Organization Quality of Life Scale)

The WHOQOL-100 is an instrument used to assess subjective quality of life. The basis of the instrument is the definition of quality of life as the individual's perception of their own life situation within the context of their culture and value system, and in relation to personal goals, expectations, standards, and interests. The WHOQOL-100 consists of 100 items, which are assigned to the dimensions of physical well-being, psychological well-being, independence, social relationships, environment, and religion/spirituality. The questionnaire discriminates well between individuals with health impairments and healthy individuals, as well as between those with physical and psychological illnesses. The internal consistency (Cronbach's alpha) of the WHOQOL-100 subscales ranges from $\alpha = .59$ to $\alpha = .91$. Age-stratified reference values are available for the age range of 18 to over 85 years ($N = 715$) (The WHOQOL Group, 1998).

DAD (Disability Assessment for Dementia)

The "Disability Assessment for Dementia" (DAD) scale is a tool used to measure functional abilities in activities of daily living (ADL) in individuals with cognitive impairments such as dementia. It evaluates both basic and instrumental activities of daily living in relation to executive functioning. The DAD assesses basic activities of daily living, instrumental activities, leisure activities, initiation of actions, planning and organization, and effective completion of tasks. The test-retest reliability is ICC = .96, and the inter-rater reliability is ICC = .95.

ZBI (Zarit Burden Interview)

The Zarit Burden Interview (ZBI) is the most commonly used instrument for assessing the subjective burden experienced by caregivers of individuals with dementia. The tool demonstrates strong psychometric properties (Cronbach's $\alpha = 0.91$) and shows good correlations as a validity indicator in relation to the caregiver's well-being, the care dependency of the individual, and the neuropsychiatric symptoms of the person with dementia (Braun et al. 2010).

PAL (Pool Activity Level)

The Pool Activity Level (PAL) Instrument assesses a person's activity level in daily living activities. It covers nine areas of activity, including tasks such as washing, dressing, eating, and communication skills. The items record how much assistance a person needs, for example, when dressing: "Needs help planning what to wear, but recognizes clothes and knows how to put them on; requires help with the sequence of dressing." The responses help determine an activity level that provides insight into both the person's abilities and the necessary assistance required. It is a validated tool for individuals with dementia (Tatzer & Pool 2018).

VAMS™ (Visual Analog Mood Scales)

The Visual Analog Mood Scales (VAMS) is a reliable and valid instrument used to measure eight specific mood states: anxiety, confusion, sadness, anger, energy, fatigue, happiness, and tension. VAMS requires minimal cognitive or verbal effort from the respondent, making it particularly suitable for neurologically impaired individuals or those unable to complete verbally or cognitively demanding instruments. Scores for each mood state range from 0 to 100, where 100 represents the maximum level of that mood and 0 indicates a minimal level (or absence) of the mood (PAR 2019).

Translation

4.5. Statistics

Quantitative Part

The data will be analyzed using IBM SPSS version 24. The significance level is set at alpha = 0.05. Descriptive statistics will be presented as mean and standard deviation or as median and quartiles, depending on the type of distribution. Absolute and relative frequencies will be used to describe categorical data. To address the primary research question—whether tablet-based training has a positive effect on cognition—both the primary outcome (performance in the neuropsychological test battery, NTB, global score) and secondary outcome parameters (sub-tests of the NTB and other neuropsychological assessments) will be compared between the intervention group (IG) and control group (CG) using ANCOVA (adjusted for multiple comparisons). Additionally, total brain volume, the volume of brain lobes, hippocampal volume, cerebral microstructure, and functional connectivity will be measured and compared between groups. Other secondary outcome parameters will also be compared between groups using ANCOVA. Pre-post comparisons (ANOVA repeated measures) will provide insights into changes over time. Analyses will be adjusted for age, gender, comorbidities, and education level.

Qualitative Part

The qualitative interviews will be organized and coded using the software program MAXQDA and analyzed through qualitative content analysis (Kimberly & Neuendorf, 2017) by the Institute of Nursing Science.

4.6. Ethical Considerations

4.6.1. Informed Consent

Only individuals who provide prior written informed consent will be included in the study. For participants with dementia who have a representative or legal guardian, written informed consent will be obtained from these representatives. All participants can withdraw from the study at any time without providing reasons. All examinations, except for blood draws, are non-invasive. Additionally, all procedures, including the 3T MRI, are conducted in clinical routine. Genetic analyses are performed in accordance with legal

Translation

requirements. The genetic analyses are conducted solely for scientific purposes and only with the written consent of the donor. All samples are processed anonymously and coded without names. Samples can only be linked to the University Clinic for Neurology with the name of the donor.

If results from genetic analyses are shared, measures will be taken to ensure that the donor cannot be identified by the collaborating centers. Except for blood draws, no harm or burden is anticipated for participants. The benefit-risk ratio is therefore considered to be high.

4.6.2. Data Protection

4.6.2.1. Data Protection for Questionnaires and Interviews

Each study participant is assigned an individual anonymized code upon inclusion or randomization, which is recorded at the University Clinic for Neurology (UK) and transmitted to the Sozialverein Deutschlandsberg (SVDL) and the Research Coordination (RK). Communication between project partners and any data transfer will occur using this code to ensure participant anonymity. Questionnaires and interviews (both individual and focus groups) are transmitted through a secure, password-protected server from the SVDL to the Medical University of Graz (MUG). All personal data of participants are handled confidentially, and interviews are anonymized during transcription. Access to data for input and analysis is limited to the University Clinic for Neurology - Clinical Department of Neurogeriatrics, the Institute for Nursing Science at MUG, and Joanneum Research Digital (research partners).

4.6.2.2. Data Proctection and Privacy for “DaheimAktiv”

„DaheimAktiv“

Login/Backend „DaheimAktiv“

To use "DaheimAktiv," users must register with an email address and a password, which are encrypted and stored in the database. Only one device (tablet) can be actively used per account, and this device is linked to the user account. The unique serial number of

Translation

the device is also recorded and associated with the user. Additional personal data is optional and not required for registration.

Completed Units „DaheimAktiv“

"DaheimAktiv" stores a data stream for each completed unit, reflecting the participant's performance during these sessions. This includes data on speed and accuracy in included exercise programs (e.g., error detection, correct/incorrect answers, quizzes). This data is essential for detecting improvements or deteriorations in participants' performance, identifying strengths, and pinpointing areas for further improvement. For general analyses of this target group, the data is anonymized.

4.6.2.3. Data Protection for Fitness Tracker

The use of the fitness tracker is linked to a user account that must be created by the user through the fitness tracker's application. There is no transmission of log data to the project's central database. Data can only be accessed by the user themselves, using their account credentials.

4.7. Benefits/Risks

4.7.1. Benefits

The use of the multimodal "DaheimAktiv" serious game on the tablet PC as a non-pharmacological intervention promotes psychological, cognitive, physical, and social abilities. The long-term benefit could be a stabilization of the disease progression. Participants' results support the further development of cognitive training programs on tablet PCs for application in caregiving practice. In particular, participants with dementia provide valuable insights into the individual needs of their target group. According to the systematic review by Span et al. (2013), individuals with dementia should be integrated at all stages of a development project to create valuable, user-friendly, supportive technologies that enhance the quality of life for this group and enable a longer stay in their own home.

Translation

4.7.2. Risks

Psychological (e.g., overload) or physical (e.g., fatigue) stress related to the use of the "DaheimAktiv" tablet PC program may occur. To minimize these stresses, the training programs are tailored to individuals with dementia (e.g., difficulty level, duration), and participants are accompanied and supported by dementia trainers throughout the study. Participants can also contact the research team at any time to discuss any issues. During MRI imaging, participants may experience discomfort. Each participant can choose to stop the examination at any time. For blood sampling, there is a slight risk of bruising, bleeding, or pain at the puncture site, and in rare cases, the risk of fainting. Participants with a tendency to bleed or a known propensity for collapse are advised against participation. For lumbar puncture, risks include bleeding and bruising, infections and inflammations, circulatory and consciousness disturbances, as well as temporary nerve damage with numbness or paralysis. It is explicitly stated that the lumbar puncture is optional.
Participation in the study can be terminated at any time without giving reasons and without any disadvantages for the patient or their relatives.

Translation

References

(ADI, 2013a) World Alzheimer Report 2013: Journey of caring: an analysis of long-term care for dementia. ADI, London.

(Allgaier et al., 2011) Validität der Geriatrischen Depressionsskala bei Altenheimbewohnern: Vergleich von GDS-15, GDS-8 und GDS-4. Psychiat Prax, 38:280–286.

(Aschenbrenner et al. 2001) Regensburger Wortflüssigkeitstest. 1. Auflage. Hogrefe, Göttingen.

(Berwig et al., 2009) Critical evaluation of self-rated quality of life in mild cognitive impairment and alzheimers disease – further evidence for the impact of anosognosia and global cognitive impairment. The Journal of Nutrition, Health & Aging, 13(3):226-230.

(Berwig et al., 2011) Self-related quality of life in mild cognitive impairment and alzheimers disease. GeroPsych, 24(1):45-51.

(Betella & Verschure, 2016) The Affective Slider: A Digital Self-Assessment Scale for the Measurement of Human Emotions. PLoS ONE 11(2):e0148037.

(Boman et al., 2014) Exploring the usability of a videophone mock-up for persons with dementia and their significant others. BMC Geriatr 14, 49. https://doi.org/10.1186/1471-2318-14-49

(Bowling et al., 2015) Quality of life in dementia: a systematically conducted narrative review of dementia-specific measurement scales. Aging & Mental Health, 19(1):13-31.

(Braunseis et al., 2012) The risk for nursing home admission did not change in ten years-a prospective chohort study with five-year follow-up. Arch Gerontol Geriatr 54:e63-e67.

Translation

(Cao et al., 2017) Realtime Multi-Person 2D Pose Estimation using Part Affinity Fields, Proc. CVPR 2017.

(Chalfont et al., 2018) A mixed methods systematic review of multimodal non-pharmacological interventions to improve cognition for people with XVII dementia, Dementia (London). 19(4):1086-1130. doi: 10.1177/1471301218795289.

(Cohen et al., 1983) A global measure of perceived stress. Journal of Health and Social Behaviour, 24:385-396.

(Cummings, 1997) The Neuropsychiatric Inventory. Neurology, vol. 48, suppl. 6, pp 10-16.

(Desmet et al., 2016) Mood Measurement with Pick-A-Mood; Review of current methods and design of a pictorial self-report scale. *J. Design Research*, vol. 14, no. 3.

(Dijkstra et al., 1996) Nursing-care dependency. Development of an assessment scale for demented and mentally handicapped patients, Scandinavian Journal of Caring Sciences, vol. 10, no. 3, pp. 137-143.

(Dijkstra et al., 1998) Operationalization of the concept of 'nursing care dependency' for use in long-term care facilities. Aust N Z J Ment Health Nurs 7(4):142-51.

(Djabelkhir et al., 2017) Computerized cognitive stimulation and engagement programs in older adults with mild cognitive impairment: comparing feasibility, acceptability, and cognitive and psychosocial effects, Clinical Interventions in Aging, vol. 12, pp. 1967-1975.

(Ehret et al., 2015) Technikbasiertes Spiel von Tagespflegebesuchern mit und ohne Demenz, Zeitschrift für Gerontologie und Geriatrie, vol. 50, no. 1, pp. 35-44.

(Fasilis et al., 2018) A pilot study and brief overview of rehabilitation via virtual environment in patients suffering from dementia, Psychiatriki, vol. 29, no. 1, pp. 42-51.

(Folstein et al., 1975) Mini-mental state. A practical method for grading the cognitive state of patients for the clinician. J Psychiatr Res 12:189-198.

Translation

(Folstein et al., 2010) MMSE-2 Manual zur Durchführung und Auswertung [MMSE-2 user’s manual]. PAR, Florida Ave.

(Garcia-Casal et al., 2017) Computer-based cognitive interventions for people living with dementia: a systematic literature review and meta-analysis, Aging Ment Health, vol. 21, no. 5, pp. 454-467.

(Gauggel et al., 1999) Validität und Reliabilität einer deutschen Version der Geriatrischen Depressionsskala (GDS), Zeitschrift für Klinische Psychologie und Psychotherapie, 28, pp. 18-27.

(Harrison et al., 2007) A Neuropsychological Test Battery for use in Alzheimer’s disease clinical trials, Archives of Neurology, vol 64, no. 9, pp. 1323-1329.

(Härting, et al., 2000) Wechsler Memory Scale – Revised Edition, German Edition. Manual. Huber, Bern 2000.

(Hensel et al., 2007) Measuring cognitive change in older adults: reliable change indices for the Mini-Mental State Examination, Journal of Neurology, Neurosurgery & Psychiatry, vol. 78, no. 12, pp. 1298-1303.

(Hensel et al., 2009) "Does a reliable decline in Mini Mental State Examination total score predict dementia? Diagnostic accuracy of two reliable change indices", Dementia and Geriatric Cognitive Disorders, vol. 27, no. 1, pp. 50-58.

(Hitch et al., 2017) Use of touchscreen tablet technology by people with dementia in homes: A scoping review, Journal of Rehabilitation and Assistive Technologies Engineering, vol. 4.

(IQPR, 2010) TGUG / TUG, Timed Get-Up and Go Test / Timed "Up and Go"-Test. Verfügbar unter: http://www.assessment-info.de/assessment/seiten/datenbank/vollanzeige/vollanzeige-de.asp?vid=370 (19.05.2018).

Transla

(Joddrell et al. 2016) Studies Involving People With Dementia and Touchscreen Technology: A Literature. JMIR Rehabil Assist Technol 2016;3(2):e10) doi:10.2196/rehab.5788 Review.

(Kahle-Wrobleski et al., 2007) Sensitivity and Specificity of the Mini-Mental State Examination for Identifying Dementia in the Oldest-Old: The 901 Study, Journal of the American Geriatrics Society, vol. 55, no. 2, pp. 284–289.

(Kaufmann et al., 2010) L. D. Kaufman, J. Pratt, B. Levine, S. E. Black, Antisaccades: A probe into the dorsolateral prefrontal cortex in alzheimer's disease. a critical review, Journal of Alzheimer's Disease vol. 19, pp. 781–793.

(Klein et al., 2016) The German version of the perceived stress scale – psychometric characteristics in a representative German community sample. BMC Psychiatry, vol 16, no. 159.

(Klimova & Maresova, 2017) Computer-based training programs for older people with mild cognitive impairment and/or dementia', Frontiers in Human Neuroscience, vol. 11, no. 262.

(Kothgassner et al., 2013) TUI Technology Usage Inventory. ICARUS (Information- and **(PAR, 2019)** VAMS™ - Visual Analog Mood Scales™ by Stern. https://www.parinc.com/Products/Pkey/471 (02.07.2019).

(Lagun et al., 2011). Detecting cognitive impairments by eye movement analysis using automatic classification algorithms, Journal of Neuroscience Methods, pp. 196-203.

(Lauriks et al., 2007) Review of ICT-based services for identified unmet needs in people with dementia. Ageing Research Reviews, 6(3):223-46.

(Lee, 2012) Review of the psychometric evidence of the perceived stress scale. Asian Nursing Research, vol. 6, no. 4, pp. 121-127.

(Lewin, 1946). Action research and minority problems. *Journal of Social Issues*, 2, 34-46.

Translation

(Lim et. al., 2012) Usability of tablet computers by people with early-stage dementia. Gerontology. 2013;59(2):174-82.

(Lohrmann et al., 2003) Care dependency:testing the German version of the care dependency scale in nursing homes and on geriatric wards, Scandinavian Journal of Caring Sciences, vol. 17, pp. 51-56.

(Lohrmann, 2003) Die Pflegeabhängigkeitsskala: ein Einschätzungsinstrument für Heime und Kliniken - Eine methodologische Studie, In Department of Nursing Science Center for Humanities and Health Science, Charité-Universitätsmedizin Berlin, Berlin.

(Lueken et al., 2006) Die Apathy Evaluation Scale: Erste Ergebnisse zu den psychometrischen Eigenschaften einer deutschsprachigen Übersetzung der Skala. Fortschr Neurol Psychiat, 74; 714 – 722.

(Mahlberg & Gutzmann, 2005) "Diagnostik von Demenzerkrankungen", Deutsches Ärzteblatt, vol. 102, no. 28-29.

(McKhann et al., 2011) The diagnosis of dementia due to Alzheimer's disease: recommendations from the National Institute on Aging-Alzheimer's Association workgroups on diagnostic guidelines for Alzheimer's disease. Alzheimers Dement, 7(3); 263-269.

(Mao et al., 2015) Indicators of perceived useful dementia care assistive technology: Caregivers' perspectives. Geriatr Gerontol Int 15(8):1049-57.

(Nasreddine et al. 2005) The Montreal cognitive assessment, MoCA: a brief screening tool for mild cognitive impairment. J Am Geriatr Soc, 53:695-699.

(NIH, WHO, 2011) Global Health and Aging http://www.who.int/ageing/publications/global_health.pdf. (05.04.2017).

(Nordheim et al., 2015) Tablet-PC und ihr Nutzen für demenzerkrankte Heimbewohner Z Gerontol Geriat 48:543-549.

(OECD, 2015) Adressing Dementia – the OECD response. OECD publishing, Paris.

Translation

(Piano et al., 2014) Real-time Automatic Emotion Recognition from Body Gestures, Proc. IDGEI 2014 Workshop, Eds., L. Paletta, B. Schuller et al., Haifa, Israel. https://doi.org/10.48550/arXiv.1402.5047

(Podsiadlo et al., 1991) The Timed "Up & Go": A Test of Basic Functional Mobilitv for Frail Elderlv Persons. Journal of the American Geriatric Society, vol. 39, no. 2, pp. 142-148.

(Rheinberg et al., 2001) FAM: Ein Fragebogen zur Erfassung aktueller Motivation in Lern- und Leistungssituationen, *Diagnostica*, 47, pp. 57-66. https://doi.org/10.1026//0012-1924.47.2.57. Hogrefe Verlag.

(Robert Koch-Institut (Hrsg), 2015) Gesundheit in Deutschland. Gesundheitsberichterstattung des Bundes. Gemeinsam getragen von RKI und Destatis. RKI, Berlin.

(Rösler et al., 2003) Overview of Standardised Diagnostic Instruments of Dementia, Fortschritte der Neurologie • Psychiatrie, vol. 71, pp. 187-198.

(Russel et al., 1999) Core affect, prototypical emotional episodes, and other things called emotion: dissecting the elephant. Journal of personality and social psychology.1999; 76(5):805.

(Schneider & Yvon, 2013) A review of multidomain interventions to support healthy cognitive ageing, The Journal of Nutrition, Health & Aging, vol. 17, no. 3, pp. 252-257.

(Schramm et al., 2002) Psychometric properties of Clock Drawing Test and MMSE or Short Performance Test (SKT) in dementia screening in a memory clinic population, International Journal of Geriatric Psychiatry, vol. 17, no. 3, pp. 254-260.

(Schüssler, 2015) Care Dependency and Nursing Care Problems in Nursing Home Residents with and without dementia, doctoral thesis, Medizinische Universität Graz.

(Sheehan et al., 2012) Assessment scales in dementia. Ther Adv Neurol Disord, 5(6):349-58.

Translation

(Smarr et al., 2012) Older adults´ preferences for an acceptance of robot assistance for everyday living tasks: 56th Annual Meeting of Human Factors and Ergonomics Society, 22-26 October, Human Factors and Ergonomics Society, Boston, pp. 153 – 157.

(Smith et al. 2005) Measurement of health-related quality of life for people with demetia: development of a new instrument (DEMQOL) and an evaluation of current methodology. Health Technology Assessment. 9(10).

(Span et al., 2013) Involving people with dementia in the development of supportive IT applications: a systematic review. Ageing Res Rev. 12(2):535-51.

(The WHOQOL Group, 1998) Development of the World Health Organization WHOQOL-BREF Quality of Life Assessment: Psychological Medicine, 28(3):551-558.

(University of Illinois, 2019) Modified Interest Checklist (Interessen Checklist). https://www.moho.uic.edu/productDetails.aspx?aid=38 (05.04.2017).

(van der Elst et al., 2006) The Letter Digit Substitution Test: Normative Data for 1,858 Healthy Participants Aged 24–81 from the Maastricht Aging Study (MAAS): Influence of Age, Education, and Sex; Journal of Clinical and Experimental Neuropsychology; 28: 998-1009.

(Wang et al., 2016) Robots to assist daily activities: views of older adults with Alzheimer's disease and their caregivers. Int Psychogeriatr 29(1):67-79.

(Wechsler, 2009) Wechsler Memory Scale – Fourth Edition. Manual. Pearson Assessment, San Antonio, TX.

(Wilson DMC and Ciliska D, 1984) Lifestyle assessment: Development and use of the FANTASTIC checklist. Canadian Family Physician 30: 1527–1532.

(Wu et al., 2014) Acceptance of an assistive robot in older adults: a mixed-method study of human-robot interaction over a 1-month period in the Living Lab setting. Clinical Interventions in Aging, 8(9):801-811.

Translation

Appendix 1 Data Collection Instruments

According to the journal's guidelines, copyrighted images have been removed from the published version.

Translation
