# Supplementary material for: The effect of tablet-based multimodal training on cognitive functioning in Alzheimer’s disease: A randomized controlled trial
Source: PLoS One. 2025 Aug 13;20(8):e0329931. doi: 10.1371/journal.pone.0329931 (PMC12349711; doi:10.1371/journal.pone.0329931)
Supplement: S2 File — (PDF) [file pone.0329931.s003.pdf]

Gemäß den Richtlinien des Journals wurden urheberrechtlich geschützte institutionelle Logos aus der veröffentlichten Version entfernt.

Studienprotokoll

Spielgestützte multimodale Intervention, Monitoring und Decision Support zur Aktivierung bei Alzheimer-Demenz

Kurzbezeichnung Projekt: MultimodAAL

Gemäß den Richtlinien des Journals wurden urheberrechtlich geschützte Abbildungen aus der veröffentlichten Version entfernt.

Ass. Prof. PD Mag. Dr. Marisa Koini[1], Sen. Scient. Dr. Sandra Schüssler[2], Univ. Prof. Dr. Reinhold Schmidt[1]

Medizinische Universität Graz

Univ. Klinik für Neurologie, Klinische Abteilung für Neurogeriatrie[1] und Institut für Pflegewissenschaft[2]

Inhalt

Zusatzinformation zum Projekt 4
1. Zusammenfassung 4
2. Hintergrund 5
3. Forschungsziele 7
4. Methode 8
4.1. Design 8
4.2. Setting und Stichprobe 8
4.2.1. Setting 8
4.2.2. Stichprobe 8
4.2.2.1. Ein- und Ausschlusskriterien 9
4.2.2.2. Rekrutierung 12
4.2.2.3. Randomisierung und Verblindung 12
4.3. Intervention 13
4.3.1. Ablauf der Studie 14
4.3.2. Schulungen 15
4.3.2.1. Datenerhebende Personen 15
4.3.2.2. Schulung der TeilnehmerInnen 15
4.3.3. Hotline, Kontaktpersonen 15
4.4. Datenerhebungsmethoden 16
4.4.1. Tabellenübersicht Erhebungsmethoden 17
4.4.1.1. Beschreibung der Erhebungsmethoden 24
4.5. Statistik 40
4.6. Ethische Aspekte 41
4.6.1. Informierte Zustimmung 41
4.6.2. Datenschutz 41
4.6.2.1. Datenschutz Fragebögen, Interviews 41
4.6.2.2. Datenschutz, Privatsphäre „DaheimAktiv“ 42
4.6.2.3. Datenschutz Fitness Tracker 43
4.7. Nutzen/Risiken 43
4.7.1. Nutzen 43

4.7.2. Risiken 43
Referenzliste.. 45
Anhang 1 Datenerhebungsinstrumente 52

Zusatzinformation zum Projekt

Folgeprojekt von AktivDaheim, gefördert durch FFG; Ethikantrag EK Nr: 1505/2016

1. Zusammenfassung

Hintergrund: Die Prävalenz der Demenz steigt weltweit an und bewirkt eine Zunahme des Bedarfs an Gesundheitsleistungen, die einer zunehmenden Versorgungslücke durch die sinkende Anzahl an verfügbaren Pflegekräften gegenübersteht. Aus diesem Grund sind viele neue Technologien, wie z. B. Tablet-PC Trainings entwickelt worden, die das Potential haben, Pflegepersonen und Personen mit Demenz bei der Förderung der Pflegeunabhängigkeit und Stabilisierung des Krankheitsverlaufes zu unterstützen. Bisherige Studien, die computer-basierte Trainings für Personen mit Demenz inkludieren, fokussieren meist lediglich auf die kognitiven und psychischen Effekte von hauptsächlich kognitiven Trainingsprogrammen. Die internationale Literatur empfiehlt aber multimodale Trainingsprogramme (z. B. auch physische und soziale Komponenten neben den kognitiven Aspekten) anzuwenden.

Ziele: Das Hauptziel ist die Untersuchung der Effektivität des multimodalen Tablet-PC Trainingsprogrammes „DaheimAktiv“ auf kognitive Fähigkeiten von Personen mit Alzheimer-Demenz im frühen Stadium. Die Nebenziele sind die Erhebung der Usability und Akzeptanz des Tablets, strukturelle und funktionelle Veränderungen im Gehirn, Lebensqualität, Mobilität, Motivation, Lebensstilfaktoren, Hobbies, Stress, Emotionen, Pflegeabhängigkeit, (instrumentelle) Aktivitäten des täglichen Lebens, Medikamentenveränderung, endogene/biologische Faktoren, Verhaltensprobleme, Pflegebelastung, Aktivitätslevel und Armkraft sowie die Erhebung einer möglichen depressiven Symptomatik.

Methoden: Es wird eine Mixed-Method Studie durchgeführt (1,5 Jahre). Der quantitative Teil ist eine randomisierte kontrollierte Studie (RCT). Der qualitative Teil inkludiert ergänzende Interviews (Einzelinterviews, Fokusgruppen). Die Interventionsgruppe (110 Personen mit Alzheimer-Demenz) erhält ein multimodales (körperliche-, kognitive- und soziale Komponenten) Tablet PC-Trainingsprogramm und die Kontrollgruppe (110 Personen mit Alzheimer-Demenz) erhält eine Standartintervention. Die Daten werden mittels Tablet-PC, Eye tracking, Fitness tracking, Leistungstests, MRT, Blutabnahme, Fragebögen und

Interviews erhoben. Ebenfalls gibt es ergänzende Fragebogenerhebungen bei den Angehörigen (n=220), den DemenztrainerInnen (n=5) und Pflegepersonen (n=5). Alle n=220 PatientInnen werden vor Trainingsbeginn (=Interventionsgruppe) bzw. Warteperiode (=Kontrollgruppe) und nach dem 1,5-jährigen Trainings-/Warteintervall einer ausführlichen neurologischen und neuropsychologischen Untersuchung unterzogen. Weiters wird zu beiden Zeitpunkten eine MRT-Untersuchung durchgeführt. Die Datenanalyse erfolgt quantitativ (deskriptive Statistik, inferenzstatistisch und qualitativ (Content Analyse)).

Die vorliegende Studie fördert die Weiterentwicklung von computerbasierten Tablet-PC Trainingsprogrammen für die Zielgruppe der Alzheimerdemenz in der Pflegepraxis.

2. Hintergrund

Der demografische Wandel bewirkt einen Anstieg an älteren Menschen mit chronischen Erkrankungen, wie Demenz (NIH & WHO, 2011; Robert Koch-Institut, 2015) und bewirkt folglich eine Zunahme des Bedarfs an Gesundheitsleistungen. Dem steht jedoch eine zunehmende Versorgungslücke durch die sinkende Anzahl an verfügbaren Pflegekräften gegenüber (Robert Koch-Institut, 2015).

Personen im früheren Stadium der Demenz werden hauptsächlich zu Hause betreut bzw. gepflegt (OECD, 2015), aber durch das Voranschreiten der Demenz und der zunehmend entstehenden Pflegebedürfnisse, aufgrund von Pflegeabhängigkeit (z. B. bei Mobilität, kognitive Lernfähigkeit) und Pflegeproblemen (z. B. Sturz, Mangelernährung), wird professionelle Pflege immer notwendiger und kann in einer institutionellen Pflege enden, wenn die Pflege zu Hause nicht mehr sichergestellt werden kann (ADI, 2013; OECD, 2015; Braunseis et al., 2012). Eine der wichtigsten Aufgaben der Pflege (und der primären Gesundheitsversorgung) ist die Unabhängigkeit von Personen mit Demenz zu fördern um einem rasch progredienten Krankheitsverlauf mit zunehmender Pflegeabhängigkeit entgegenzuwirken (Schüssler, 2015). Hier könnten computergestützte Technologien, wie z. B. Tablet PC Trainings eine Unterstützung für Pflegepersonen darstellen. Diese haben nicht nur das Potential die (Pflege-) Unabhängigkeit von älteren Personen zu fördern,

sondern auch das Wohlbefinden zu steigern (Smarr et al., 2012; WHO, 2007), was letztlich auch eine Stabilisierung im Krankheitsverlauf bewirken kann.

Bisher werden neue Technologien, wie z. B. Tablet PCs, erst im geringeren Ausmaß in der praktischen Pflege eingesetzt. In der Studie von Nordheim et al. (2015) wurden Tablet PCs bei Personen mit schwerer Demenz getestet. Der Einsatz zeigte positive Ergebnisse in Hinblick auf z. B. Kommunikation, Motivation und Verhalten der BewohnerInnen. Die Zielgruppe waren Personen im Pflegeheim, weswegen Rückschlüsse auf Personen im Setting zu Hause nur eingeschränkt gezogen werden können. Die Studie von Lim et. al. (2012) untersuchte die Usability des Einsatzes eines Tablet PCs bei Personen mit leichter Demenz im häuslichen Umfeld und kam zum Ergebnis, dass die Hälfte der TeilnehmerInnen das Tablet alleine, mit minimaler Unterstützung, anwenden konnte, obwohl 95% der Personen vorab keine Erfahrung im Umgang mit Tablet PCs hatten. Im Review von Joddrell et al. (2016) zeigten 45 inkludierte Studien, dass Personen mit Demenz im Umgang mit Touchscreen Technologien gut zurechtkommen. Internationale Forschungsergebnisse zeigen die Notwendigkeit, Personen mit Demenz in Studien zu inkludieren (Wu et al., 2014; Span et al., 2013; Mao et al., 2015), um auch deren Feedback und Empfehlungen für die Weiterentwicklung neuer Technologien zu erhalten (Boman et al. 2014). Allgemein wünschen sich Menschen mit Demenz Technologien, die kognitive Unterstützung bieten, Kommunikation, soziale Interaktion und Lebensaktivitäten (z. B. Mobilität) fördern (Lauriks et al., 2007; Wang et al., 2016).

Bisherige Studien, die computer-basierte Trainings für Personen mit Demenz mittels Tablet-PC inkludieren, fokussieren meist lediglich auf die kognitiven und psychischen Effekte von hauptsächlich kognitiven Trainingsprogrammen (Djabelkhir et al. 2017; Ehret et al. 2015; Fasilis et al. 2018; Garcia-Casal et al. 2017; Hitch et al. 2017; Klimova & Maresova 2017; Nordheim et al. 2015). Die Arbeiten von Schneider & Yvon (2013) sowie Chalfont, Milligan and Simpson (2018, ein systematischer Review) legen aber nahe, dass Interventionen effektiver sind, wenn diese multimodal durchgeführt werden (also z. B. auch physische und soziale Komponenten neben den kognitiven Aspekten inkludieren). Die vorliegende Studie beinhaltet ein multimodales PC-Trainingsprogramm für Personen mit Demenz, welches Effekte auf kognitiver-, psychischer-, physischer- und ADL- (Aktivitäten

des täglichen Lebens/Pflegeabhängigkeit) Ebene untersucht, und somit die Weiterentwicklung von computer-basierten Trainings für die Zielgruppe Demenz unterstützten soll.

3. Forschungsziele

Hauptziel

Untersuchung der Effektivität des multimodalen Tablet-PC Trainingsprogrammes „DaheimAktiv" auf kognitive Fähigkeiten der Personen mit Alzheimer-Demenz im frühen Stadium.

Nebenziele

- Quantitativ: Erhebung struktureller und funktioneller Veränderungen im Gehirn (z. B. globales und lokales Hirnvolumen, Marklagerveränderungen), Lebensqualität, Mobilität, Motivation, Lebensstilfaktoren, Stress, Pflegeabhängigkeit, (instrumentelle) Aktivitäten des täglichen Lebens, Medikamentenveränderung, endogene/biologische Faktoren (d.h. die Bedeutung von DNA Varianten, DNA Methylierungsprozessen bzw. Veränderungen in der Genexpression in Bezug auf Demenzerkrankung), Verhaltensprobleme, Pflegebelastung, Aktivitätslevel und Armkraft sowie die Erhebung einer möglichen depressiven Symptomatik und die Nutzung der erhobenen Daten für die Entwicklung eines decision support systems.
- Quantitativ: Untersuchung der Usability und Akzeptanz bezüglich der Anwendung des Trainings
- Qualitativ: Ergänzende Interviews (Fokusgruppen und Einzelinterviews) zur Usability

4. Methode

4.1. Design

Bei der Studie handelt es sich um eine „Mixed-Methods Studie“ mit „Embedded Design“. Das Design des quantitativen Anteils ist eine „Randomisierte kontrollierte Studie“ (RCT). Beim qualitativen Anteil handelt es sich um ergänzende Interviews (Einzelinterviews und Fokusgruppen) mit Content Analyse. Die RCT wird in der Datenbank ClinicalTrials.gov registriert (https://www.clinicaltrials.gov/ct2/home). Es handelt sich um eine explorative Studie mit dem Ziel neue Zusammenhänge zwischen einem Tablet-basierten Training und möglichen Outcomes (z. B.: Kognition, Motivation, Lebensqualität) zu untersuchen.

4.2. Setting und Stichprobe

4.2.1. Setting

Die Studie wird in der Steiermark durchgeführt. Die TeilnehmerInnen mit Demenz kommen aus dem häuslichen Setting, dem betreuten Wohnen und aus dem Pflegeheimbereich.

4.2.2. Stichprobe

Es sollen 220 PatientInnen (Abb. 1) mit Alzheimer-Demenz diagnostiziert nach den NINCDS-ADRDA Kriterien eingeschlossen werden. Ergänzend dazu deren Hauptangehörige/Bezugsperson/Begleitperson (n=220) sowie 5 Pflegepersonen und 5 M.A.S. (Morbus Alzheimer Syndrom) TrainerInnen (Personen, die stadienspezifisches Training für Personen mit Demenz in Einzel- und Gruppentrainings in unterschiedlichen Pflegesettings durchführen).

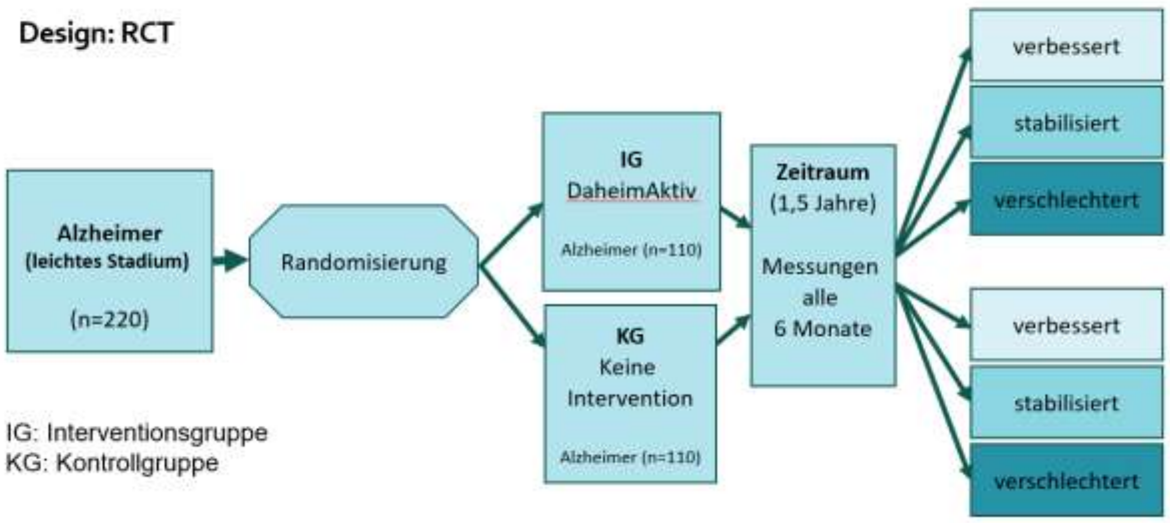

Abbildung 1: Überblick über die randomisierte kontrollierte (RCT) Studie

4.2.2.1. Ein- und Ausschlusskriterien

Person(en) mit Demenz (PmD)

Einschlusskriterien:

- Eine Diagnose möglicher oder wahrscheinlicher Demenz gemäß den NINCDS-ADRDA Kriterien (McKhann et al. 2011).
- Die PmD ist älter als 40 Jahre bei der Baseline-Untersuchung.
- Die PmD spricht und versteht die deutsche Sprache und verfügt über ausreichend physische, auditorische und visuelle Fähigkeiten zur Durchführung einer neuropsychologischen Untersuchung bzw. einem Tablet-basiertem Training.
- Die PmD erhält seit min. 3 Monaten vor der Baseline Visite eine stabile medikamentöse Therapie.
- Eine Therapie mit Memantine muss 3 Monate vor der Baseline Visite begonnen worden sein.
- Angehörige(r)/Bezugsperson/Begleitperson begleitet PmD zur neurologischen Untersuchung an die UK für Neurologie bei T0 und T3

- Bei der PmD mit bezahlter 24-Stunden-Betreuung muss ein Angehöriger als TeilnehmerIn/Begleitperson rekrutiert werden.
- Die PmD lebt mit oder ohne Bezugsperson zu Hause (falls alleine sollten Angehörige in der Nähe wohnen).
- Die PmD hat professionelle (z. B. Hauskrankenpflege) und/oder nicht professionelle Pflege (z. B. durch Angehörige) oder er/sie erhält noch keine Pflege.
- Die PmD ist einwilligungsfähig bzw. ein Angehöriger mit Vertretungsbefugnis oder Sachwalter gibt die Zustimmung zur Teilnahme an der Studie
- Die PmD nimmt keine Antipsychotika und Antidepressiva bzw. ist seit min. 14 Tagen vor dem Studienstart darauf eingestellt.

Ausschlusskriterien:

- Die PmD hat in den letzten 3 Monaten vor der Baseline Visite an einer klinischen Studie teilgenommen.
- Die PmD kann die Studie voraussichtlich nicht beenden.
- Irgendeines der folgenden Zeichen im Schädel-MRT:
    - ✓ Infarkt in der Nähe großer Gefäße
    - ✓ Mehr als einen lakunären Infarkt, definiert über eine fokale Läsion in der CSF Signalintensität mit einem Durchmesser von weniger als 1.5 cm in alle Raumrichtungen.
    - ✓ Einen lakunären Infarkt in einer strategisch wichtigen Region wie dem Thalamus, Hippokampus in beiden Hemisphären oder im Nucleus Caudatus.
    - ✓ Konfluierende Läsionen der tiefen weißen Substanz (Fazekas Score 3).
    - ✓ Andere fokale Läsionen die für den kognitiven Status des Patienten/der Patientin verantwortlich sein könnten (z. B.: Infektionen, raumfordernde Läsionen, Normaldruckhydrocephalus)
    - ✓ Die PmD hatte in den letzten 3 Monaten vor der Baseline Visite eine Operation mit Vollnarkose bzw. im Studienzeitraum eine Operation geplant.
- Die PmD hat oder hatte eine klinisch signifikante immunmodulatorische Behandlung bzw. wird zukünftig eine erhalten.

- Die PmD weist eine Krebserkrankung auf (letzte Behandlung ≤ 5 Jahre vor der Baseline Visite).
- Die PmD hatte einen Myokardinfarkt innerhalb der letzten 2 Jahre vor der Baseline Visite.
- Die PmD hat Hepatitis B, C, HIV oder Syphilis.
- Die PmD hat eine aktive ansteckende Erkrankung.
- Die PmD leidet unter einer systemischen Erkrankung, die einen raschen Abbau der Person wahrscheinlich macht oder deren Sicherheit einschränken:
    - ✓ nicht ausreichend eingestellte Herzinsuffizienz (NYHA>3)
    - ✓ BMI>40
    - ✓ schlecht eingestellte Diabetes
    - ✓ schwere Niereninsuffizienz
    - ✓ chronische Lebererkrankung
    - ✓ andere klinisch relevante systemische Erkrankungen.
- Die PmD leidet unter Hypothyreose. Personen mit behandelter Hypothyreose dürfen an der Studie teilnehmen gegeben, wenn eine stabile Therapie seit >3 Monaten vor der Baseline Visite besteht.
- Die PmD leidet unter einer psychiatrischen Erkrankung, wie Schizophrenie, psychotischen Erkrankungen oder einer bipolaren Störung.
    - ✓ Die PmD hat gegenwärtig eine depressive Episode (Geriatrische Depressionsskala GDS >=6 bei der Baseline Visite) oder eine Major Depression innerhalb der letzten 2 Jahre.
- Die PmD hat eine metabolische oder toxische Enzephalopathie oder eine Demenz aufgrund der allgemeinen medizinischen Kondition.

Angehörige/Bezugspersonen/Begleitperson

Einschlusskriterien:

- Erwachsene Angehörige (Frauen und Männer) der teilnehmenden PmD
- Leben mit der PmD im gemeinsamen Haushalt oder auch nicht
- PmD erhält oder erhält keine professionelle Pflege
- Angehöriger leistet keine oder leistet selbst Pflege

- Sprechen und verstehen Deutsch
- Sind einwilligungsfähig

Professionelle Pflegepersonen

Einschlusskriterien:

- Erwachsene Frauen und Männer
- Diplomierte Pflegepersonen oder Pflegeassistenz
- Sprechen und verstehen Deutsch
- Sind einwilligungsfähig

M.A.S. (Morbus Alzheimer Syndrom) TrainerInnen

Einschlusskriterien:

- Erwachsene Frauen und Männer
- Ausgebildete M.A.S. TrainerIn
- Trainieren die teilnehmenden PmD
- Sprechen und verstehen Deutsch
- Sind einwilligungsfähig

4.2.2.2. Rekrutierung

Die TeilnehmerInnen werden über die MitarbeiterInnen des Sozialvereines Deutschlandsberg (SVDL) und des Österreichischen Roten Kreuzes (ProjektpartnerInnen) per Telefon, Flyer, Anzeigen, Website, Veranstaltungen, sozialen Medien und persönlich für die Studie rekrutiert. Beim Sampling handelt es sich um ein „Convenience Sampling". Zusätzlich werden auch Informationsflyer im SVDL und in Arztpraxen aufgelegt.

4.2.2.3. Randomisierung und Verblindung

Randomisierung der Personen mit Demenz

Ein Randomisierungsplan wird vorab erstellt. Die Zuteilung zur Interventions (IG)- und Kontrollgruppe (KG) erfolgt vorab mittels einer in Matlab erstellten Zufallsliste. Von den

StudienteilnehmerInnen 1-110 werden 70 Prozent der IG und 30 Prozent der KG zugeteilt. Von 111-220 werden 30 Prozent der TeilnehmerInnen der IG und 70 Prozent der KG zugeteilt. Dies soll gewährleisten, dass bei potentiellen Einschlussschwierigkeiten ausreichend viele TeilnehmerInnen die Intervention erhalten. Es soll hier ausdrücklich darauf hingewiesen werden, dass die Intervention und nicht die paritätische Verteilung zwischen Interventions- und Kontrollgruppe angestrebt wird. An der UK für Neurologie wird ausschließlich die Study Nurse wissen, ob ein Patient in der IG oder der KG ist. Den Untersuchern (Neurologe, Neuropsychologe) ist die Zuteilung zur Gruppe nicht bekannt.

<u>Verblindung</u>

Eine Verblindung der Mitarbeiter und Mitarbeiterinnen des SVDL und des ÖRK im Rahmen der Studie ist nicht möglich da die Intervention ersichtlich ist.

4.3. Intervention

<u>„DaheimAktiv“:</u>

Gemäß den Richtlinien des Journals wurden urheberrechtlich geschützte Abbildungen aus der veröffentlichten Version entfernt.

Abbildung 2: “DaheimAktiv”

„DaheimAktiv“ ist eine App auf einem Tablet-PC, die ein Serious Game beinhaltet welches in einem Vorprojekt (AktivDaheim, gefördert durch FFG; Ethikantrag EK Nr: 1505/2016) für PmD weiterentwickelt und getestet wurde. „DaheimAktiv“ stimuliert als multimodales Training auf spielerische Weise zu kognitiven und körperlichen Übungen. Diese können individuell auf die jeweilige Person abgestimmt werden (z. B. Inhalt, Schwierigkeitsgrad angepasst an die Demenzstufe, Ablauf, Zeit). „DaheimAktiv“ startet immer mit Bewegungsübungen inklusive Musik, die mittels Text am Tablet und Video genau erklärt werden. Danach folgen kognitive Übungen, Wissensfragen (Quiz), Fehlersuchbilder, Puzzles, Bildpaare suchen, Lückentexte, Rechenaufgaben, Höraufgaben und Lieder. „DaheimAktiv“ kann sowohl zu Hause als auch in Betreuungseinrichtungen (z. B. Pflegeheimen) angewendet werden.

4.3.1. Ablauf der Studie

Im ersten Schritt erfolgt ein Präscreening potentieller TeilnehmerInnen mittels einer verkürzten Liste der Ein- und Ausschlusskriterien über den SVDL und dem Österreichischen Roten Kreuz. Potentielle PatientInnen werden dann (Zeitpunkt T0-a, siehe **Tabelle 1**) an der Medizinischen Universität Graz, UK für Neurologie, einer klinisch-neurologische Untersuchung (inkl. Vitalwerte), einer Blutabnahme, und einer kognitiven Testung (Leistungstests und Fragebögen) unterzogen. Basierend auf der klinisch-neurologischen Untersuchung sowie der kognitiven Testung wird entschieden ob der/die TeilnehmerIn eine strukturelle und funktionelle Magnetresonanztomographie (MRT) (T0-b) um eine Alzheimerdiagnose im frühen Stadium medizinisch zu bestätigen. Parallel dazu werden die Angehörigen/Bezugspersonen/Begleitpersonen hinsichtlich pflegerischer und persönlicher Daten befragt. Bei den danach in die Studie eingeschlossenen Personen mit Alzheimer-Demenz werden nach 6 (T1) und 12 (T2) Monaten weitere Daten (siehe **Tabelle 1**) erhoben. Nach dem 1,5-jährigen Tablet Training findet erneut eine klinisch-neurologische Untersuchung inkl. Blutabnahme, eine kognitive Testung inkl. Fragebögen sowie eine strukturelle und funktionelle MRT (T3) statt.

<u>Interventionsgruppe (IG)</u>

Die IG unternimmt ein 1,5-jähriges Tablet PC-basiertes Training kognitiver und körperlicher Fähigkeiten mittels DaheimAktiv, welches 14-tägig durch eine MAS TrainerIn, DGKP oder pädagogisch ausgebildete Person des Sozialvereins Deutschlandsbergs (SVDL) oder dem österreichischen Roten Kreuz (ÖRK) gemeinsam mit der PmD durchgeführt wird. Das Tablet verbleibt bei den PmD zu Hause und die TeilnehmerInnen werden angehalten auch ohne Anwesenheit einer TrainerIn weiter zu trainieren (mit und auch ohne eines Angehörigen). Alle Trainingssitzungen der PmD am Tablet werden aufgezeichnet. DaheimAktiv ist ein multimodales Interventionsverfahren mit kognitiven und physischen Übungen. Neben der vierzehntägigen Trainingseinheiten mit den TrainerInnen und dem eigenständigen Training werden zur Förderung der Motivation sowie zur Steigerung der sozialen Integration „Trainings-Cafés“ in Kleingruppen stattfinden, d.h. mehrere PmD trainieren gemeinsam.

Kontrollgruppe (KG)

In der Kontrollgruppe werden die abseits bzw. unabhängig der Studie eingeleiteten medizinischen Interventionen fortgesetzt (Standardintervention). Ansonsten unterliegen die PmD der KG denselben Studienbedingungen wie die PmD der Interventionsgruppe. PmD der KG erhalten nach Abschluss der Evaluationsphase eine kostenfreie, einjährige Lizenz für DaheimAktiv.

4.3.2. Schulungen

4.3.2.1. Datenerhebende Personen

Fragebögen, Interviews

Um die Inter-Rater Variabilität zu minimieren werden die datenerhebenden Personen des Sozialvereins Deutschlandsberg und des Roten Kreuzes zuvor von MitarbeiterInnen der MUG Neurologie und Pflege auf die Fragebögen und Interviews (Einzelinterviews, Fokusgruppen) geschult.

Interventionstraining

Der Sozialverein Deutschlandsberg und das Rote Kreuz werden ihre DemenztrainerInnen für die Interventionsanwendung schulen. Die Dauer wird ca. 4 Stunden betragen.

4.3.2.2. Schulung der TeilnehmerInnen

Alle TeilnehmerInnen erhalten eine Schulung für die Anwendung der Intervention durch den Sozialverein Deutschlandsberg.

4.3.3. Hotline, Kontaktpersonen

Während der Studie wird vom Sozialverein Deutschlandsberg eine Hotline für Fragen und Probleme der TeilnehmerInnen während der Studienzeit eingerichtet. Reguläre Zeiten werden dafür angeben.

4.4. Datenerhebungsmethoden

In der folgenden **Tabelle 1** sind die Datenerhebungsmethoden der Studie zusammengefasst dargestellt. Danach erfolgt eine kurze Beschreibung.

4.4.1. Tabellenübersicht Erhebungsmethoden

Tabelle 1: Überblick Erhebungsmethoden der Studie

Daten	Datenerhebungsmethode	Zielgruppe (Self- oder Proxyassessment) oder autonom	Dauer	Häufigkeit<br>T0-a, T0-b=Baseline<br>T1=nach 6 Monaten<br>T2=nach12 Monaten<br>T3=nach18 Monaten<br>Laufend
**Grundcharakteristiken der Stichprobe, wie z. B.**<br>• Alter<br>• Geschlecht<br>• Bildung<br>• Frühere Berufe<br>• Erstdiagnose Demenz<br>• Wohnort<br>• Lebenssituation<br>• ICD-10 Diagnosen<br>• Gesetzliche Pflegestufe<br>• Medikamente	Fragebogen	**PmD:**<br>Self und/oder Proxy (z. B. Angehöriger, (Haus)arzt, Pflegeperson)	Je nach Erreichbarkeit der Auskunftspersonen	T0-a<br><br>T3
**Lebensstilfaktoren**	Fantastic Lifestyle Checklist	**PmD:**<br>Self mit Hilfestellung	7 min.	T0, T1, T2, T3
	<u>Tablet PC („DaheimAktiv“)</u><br><br>• [SOA] Soziale Aktivitäten: „Pick-a-social-activity“<br>• (Stress: siehe “Pick-a-mood”)	**PmD:**<br>Self mit Hilfestellung	SOA: 20 Sek.<br>STI: 20 Sek.<br>ADI: 2 Min.<br>BLU: 10 Min.<br>ERN: 20 Sek.	Mind. alle 2 Wochen (mit MAS TrainerIn – nur in IG)

	• [STI]Stimmung: “Pick-a-mood”<br>• [ADI] Adipositas: GUI[1] BMI Daten mit aktuellem Gewicht, Größe (Default-Werte).<br>• [BLU] Blutdruck: GUI Eingabe systolisch/diastolisch aktuelle Werte<br>• [ERN] Ernährung: „pick-a-diet“<br>• [RAU] Rauchen: “pick-a-smoking-behavior”<br>• [BEW] Bewegung: „pick-a-mobility“/Messung aus Fitness Tracker<br>• [SCH] Schlaf: “pick-a-sleep”		RAU: 20 Sek.<br>BEW: 20 Sek.<br>SCH: 20 Sek.<br><br>Summe: 3 (ohne BLU) bis 13 Min. (mit BLU)	
**Grundcharakteristiken der Stichprobe wie**<br>• Alter<br>• Geschlecht<br>• Bildung<br>• Wohnort<br>• ICD-10 Diagnosen<br>• Betreuungssituation und Pflegeleistung<br>• Erwerbstätigkeit	Fragebogen	**Angehörige:**<br>Self	7 min.	T0-a
**Grundcharakteristiken der Stichprobe wie**<br>• Alter<br>• Geschlecht	Fragebogen	**Pflegepersonen:**<br>Self	3 min.	T0-a

[1] Graphical User Interface: Interaktive Eingabe am Tablet PC (mit Software DaheimAktiv)

• Bildung<br>• Wohnort<br>• Berufserfahrung<br>• Beruf				
**Grundcharakteristiken der Stichprobe wie**<br>• Alter<br>• Geschlecht<br>• Bildung<br>• Wohnort<br>• Berufserfahrung<br>• (Früherer) Beruf	Fragebogen	**M.A.S. TrainerInnen:**<br>Self	3 min.	T0
**Klinisch-neurologische Untersuchung inkl. Vitalwerte**		**PmD:**<br>Self	45 min	T0-a, T3
**Kognitiver Status**	MMSE (Mini Mental State Examination)	**PmD:**<br>Self	15 min.	T0-a, T3
	Neuropsychologische Testbatterie<br>• Wechsler Memory Scale (WMS-III-R) - Visuelle und verbale Paarerkennung<br>• Verbaler Lern- und Merkfähigkeitstest (VLMT) unmittelbarer und verspäteter Abruf<br>• Wechsler Memory Scale, Revised, Zahlenspanne<br>• Regensburger Wortflüssigkeitstest (RWT)	**PmD:**<br>Self	60 min	T0-a, T3

	• Wechsler Memory Scale delayed recall<br>• Letter Digit Substitution Test (LDST)<br>• Trail Making Test A/B			
	TAP (Testbatterie zur Aufmerk-samkeitsprüfung)	**PmD:**<br>Self	5 min	T0-a, T3
**Motivation**	AES (Die Apathy Evaluation Scale)	**PmD:**<br>Self (mit Hilfestellung) und<br>proxy durch Angehö-rigen	5 min.	T0, T3<br><br>T0, T1, T2, T3
	FAM (Fragebogen zur Erfas-sung aktueller Motivation in Lern- und Leistungssituatio-nen)	**PmD:**<br>Self (mit Hilfestellung)<br>18 Fragen, 1-5	7 min.	alle 6 Monate vor dem Tab-let Training
**Lebensqualität**	DEMQOL (Dementia Quality of Life)	**Proxy durch Ange-hörigen**	10 min.	T0, T3
	WHOQOL 100 (World Health Organization Quality of Life Scale)	**Angehörige:**<br>Self	30 min.	T0-a, T3
**Pflegeabhängigkeit (ADL)**	PAS (Pflegeabhängigkeits-skala)	**Proxy durch Ange-hörigen**	5 min.	T0, T3
**Aktivitätslevel (ADL)**	Pool Activity Level (PAL) Checkliste	**Proxy durch Ange-hörige**	5 min	T0, T3
**Mobilität**	Aktivitätsanalyse über Tablet PC	**PmD:**<br>Autonom Sensoren	Während „Da-heimAktiv“	Laufend (bei Bewegungsü-bungen)
	Fitnesstracker oder MOVE	**PmD:**<br>Autonomer mobiler Sensoren	Ganztägig (mor-gens bis abends, nicht über Nacht)	Während ge-samter Stu-diendauer

	TUG (Timed UP and GO Test)	**PmD:**<br>Proxy durch DatenerheberIn	5 min.	T0-a,T3
**Globale Kognition**	MoCA (Montreal Cognitive Assessment), deutsche Version	**PmD:**<br>Proxy durch DatenerheberIn	15 Min.	T0, T1, T2, T3
**(Instrumentelle) Aktivitäten des täglichen Lebens**	Disability Assessment for Dementia Scale (DAD)	**PmD:**<br>Proxy durch Angehörige	15 min	T0-a, T3
**Exekutive Funktionen/Kognitive Kontrolle**	MIRA (Mobile Instrumental Review of Attention): Analyse des Blickverhaltens mit Eye-Tracker am/im Tablet PC mittels spezieller spielerischer („Serious Game“) Übungen:<br>• Trail Making Test A, B<br>• Anti-Sakkadentest<br>• Go/Nogo Test<br>• Spot-the-difference<br><br>Test of Attentional Performance (TAP): Sustained attention, executive functions	**PmD:**<br>Autonom durch Eye-Tracker	Laufend während Training am Tablet-PC (freiwillig)	Alle 2 Wochen am Tablet mit der MAS TrainerIn („Pflichtübung“)
**Messung der Armkraft**	Armkraft	**PmD:**<br>Self	2 min.	T0-a, T3
**Psychiatrische Faktoren**	Neuropsychiatric Inventory (NPI)	**Proxy durch Angehörige**	10	T0-a, T3
**Depression bzw. depressive Symptomatik**	GDS (The Geriatric Depression Scale)	**PmD:**<br>Self	5 min	T0-a, T3
**Affektive Stimmung**	Affective Slider	**PmD:**<br>Self (am Tablet)	6 min	Alle 14 Tage vor dem Tablet Training

**Usability**	TUI (Das Technology Usage Inventory)	**PmD, Angehörige, Pflegepersonen, TrainerInnen**<br>Self	10 min für Personen mit Demenz, ansonsten 5 min	T0, T3
**Usability, Akzeptanz**	Einzelinterviews	**PmD:**<br>durch DatenerheberIn	Ca. 20-50 min.	1x<br>(nach der Inter-vention)
	Fokusgruppen	**Angehörige, Pflegepersonen, DemenztrainerInnen**	Jeweils ca. 1½ - 2 Stunden pro Zielgruppe	1x<br>(nach der Intervention)
**Pflegebelastung, Wahrgenommenes Stressempfinden**	ZBI (Zarit Burden Interview)	**Angehörige:**<br>Self	10 min.	T0, T3
	PSS-10 (Perceived Stress Scale)	**Angehöriger:**<br>Self	5 min.	T0, T3
**MRT des Gehirns**		**PmD:**<br>Self	45 min.	T0-b, T3
**Blutabnahme**		**PmD:**<br>Self	5 min	T0-a, T3

Laut Tabelle ergeben sich drei Messzeitpunkte an der Univ. Klinik für Neurologie/Neuroradiologie. Zum ersten Zeitpunkt erfolgen bei der Person mit Demenz eine klinisch-neurologische Untersuchung, eine neuropsychologische Untersuchung sowie eine Blutabnahme. Die Gesamtuntersuchungsdauer wird etwa zweieinhalb Stunden betragen. Sollte die Person mit Demenz nach diesem Untersuchungstag noch den strengen Ein- bzw. Ausschlusskriterien entsprechen, wird sie zu einem weiteren Termin eingeladen. Am zweiten Untersuchungstermin wird eine MRT durchgeführt. Die MRT dauert etwa 45 Minuten. Am Ende des Tablet-basierten Trainings, nach 1,5 Jahren, werden alle Untersuchungen nochmals wiederholt (Dauer: etwa drei Stunden). Zwischen den einzelnen ärztlichen und psychologischen Untersuchungen finden Pausen statt bzw. wird auf Wunsch des Patienten immer eine Pause gemacht. Weiters wird den Patienten Zeit für eine Mahlzeit eingeräumt. Zur Entlastung des Patienten und der Patientin bzw. der Angehörigen am Tag der Untersuchungen an der Univ. Klinik für Neurologie werden Fragebögen, die vom Untersuchungsleiter keine neuropsychologische Ausbildung beim Ausfüllen erfordern, von den Patientinnen und Patienten gemeinsam mit den Angehörigen bzw. einem Mitarbeiter des SVDL oder des ÖRK zu Hause ohne Zeitdruck ausgefüllt bzw. bearbeitet. Klinische Tests werden von Psychologinnen und Psychologen durchgeführt. Die Fragebögen werden am Beginn, nach 6, 12 und 18 Monaten vorgegeben und die jeweilige Gesamtausfülldauer beträgt weniger als eine halbe Stunde sowohl für den Patienten als auch den Angehörigen.

Wegen der möglichen hohen Belastung für den Patienten und den Angehörigen an den Tagen der Aufenthalte an der Univ. Klinik für Neurologie, sowie aufgrund der ressourcenintensiven Untersuchungen und des Datenmanagements erfolgt nach den ersten 10 eingeschlossenen Patienten eine Evaluierung der Zumutbarkeit des Studiendesigns. Es sollen einerseits die Patienten und die Angehörigen nach jedem Untersuchungstag bezüglich der Dauer und der Belastung der einzelnen Untersuchungen (neurologische und neuropsychologische Untersuchung, Blutabnahme und MRT) und hinsichtlich der Gesamtuntersuchung befragt werden. Sollte von den Patienten und den Angehörigen mehrheitlich auf eine Unzumutbarkeit hingewiesen werden, wird das Studienprotokoll adaptiert. Hinsichtlich der Personalressourcen an der Univ. Klinik für Neurologie wird die Belastung laufend evaluiert und das Studienprotokoll gegebenenfalls angepasst. In der derzeitigen

Studienplanung sind drei Ärzte, ein Techniker, fünf Psychologen und eine Studienkoordinatorin involviert denen die entsprechenden Aufgaben zugeteilt werden.

4.4.1.1. Beschreibung der Erhebungsmethoden

Erhebungen durch den Tablet PC

Erhebung und Analyse der Aktivität der Personen aus Videomerkmalen Tablet PC

Um die Aktivierung der Personen während des Bewegungstrainings zu schätzen und um auf dieser Basis auf den motivationalen Status der Personen mit Demenz schließen zu können, wird eine Analyse der Bewegung durch eine Videokamera durchgeführt, die im Tablet PC implementiert ist. Für die korrekte Aufnahme muss der Tablet PC lediglich aufrecht auf einen Tisch aufgestellt und die Kamera in Richtung Ausübender des Bewegungstrainings ausgerichtet werden.

Zu Beginn jeder Bewegungsübung wird ein Video mit 20 Sekunden Aufnahmedauer von der Webkamera aufgenommen und im Datenspeicher abgelegt, es wird ausschließlich der Personalcode der Nutzerin mitgespeichert. Diese Kamera generiert einen Output Stream mit 640*480 Pixeln mit 30 fps (Frames pro Sekunde) oder 2560*1920 Pixel mit 1fps. Diese Videos werden bei Rückgabe des Tablet PC vom Studien-Team der JOANNEUM RESEARCH (JR) auf einen durch eine Firewall geschützten PC der JR abgelegt und durch eine Bildanalyse-Software, die nach der Methode von (Cao et al., 2017), die Skeleton-Daten einer Person im Video extrahiert und codiert ablegt, analysiert **(Abbildung 2**). Das Video wird sodann von einer automatisierten Software gelöscht, dadurch kann kein Mitarbeiter der JR Einsicht in das Erscheinungsbild der Nutzer/innen erhalten. Es werden lediglich die Skeleton-Daten der Nutzerin gespeichert, um kinetische Energie und Emotionen der Nutzerinnen, der Methodologie von (Piano et al., 2014) folgend, zu extrahieren. Schließlich werden nur die Merkmale wie Emotion und Energie für bestimmte Beobachtungszeiträume je anonymisierter Nutzerin gespeichert, alle anderen Daten werden gelöscht.

Gemäß den Richtlinien des Journals wurden urheberrechtlich geschützte Abbildungen aus der veröffentlichten Version entfernt.

(a) (b)

Abbildung 3: Exemplarische Video-Analyse der Bewegungen einer NutzerIn. Der Algorithmus von (Cao et al., 2017) ermöglicht die Extraktion von Skelton-Daten aus Videobildern. Ausschließlich die Skeleton-Daten werden gespeichert, mit anonymisierten Code der Testperson, die Videodaten werden unmittelbar nach der Analyse gelöscht. Aus den Skeleton-Daten wird die Aktivierung der NutzerInnen

Erhebung und Analyse des Blickverhaltens Tablet PC

Zur Analyse des Blickverhaltens wird in der Studie eine Software zur Analyse der Daten der Webkamera auf dem Tablet PC installiert. Die Videodaten werden in Echtzeit in der Form eines statischen Eye-Trackers am Tablet PC analysiert, die Videodaten werden schließlich nicht gespeichert, sondern, nach einer initialen Kalibrierung der Blickgeometrie, es werden lediglich die Metadaten, i.e., Fixationen und Sakkaden der Probandinnen bei der Bedienung des Tablet PCs automatisiert und kontinuierlich aufgezeichnet. Es werden Trainingseinheiten für die Antisakkaden-Aufgabe und den „Visual Paired Comparson“ Test derart präpariert, dass die Probandin nicht bewusst die Analyse des Blickverhaltens erfährt, sondern sich der gestellten Aufgabe widmet und dabei die Evaluierung der Exekutiven Funktionen (inhibitorische Funktionalität: Diamond et al., 2013) ermöglicht.

Folgende Merkmale werden dabei analysiert:

<u>Antisakkaden-Aufgabe</u>

Der Probandin werden auf einem 10,5’’ Tablet PC Bildschirm abwechselnd ein Prosakkaden- Aufgaben- und zwei Antisakkaden-Aufgaben-Blöcke in einer spielerischen Form präsentiert (24 Versuche pro Block; (gemäß Kaufmann et al., 2010). Es werden jeweils ein zentraler Stimulus 2000 ms präsentiert, gefolgt von einem Sternstimulus mit 1000 ms Präsentationsdauer in der Peripherie links oder rechts des Zentrums.

Jeder Versuch in der Antisakkaden-Aufgabe wird klassifiziert als:

- „kein Fehler“: wenn die Probandin nicht auf den Stimulus fixiert (in keinem Messzeitpunkt näher als 2° zu der Blickgerade ausgerichtet ist, die durch den Stimulus gerichtet ist).
- „Fehler“: wenn die Blickgerade in mindestens einem Messzeitpunkt innerhalb des Schwellwertwinkels verläuft.
- „korrigierter Fehler“: wenn die Blickgerade nach dem „Fehler“ wieder außerhalb des Schwellwertwinkels gerät, bevor der Versuch terminiert.
- „unkorrigierter Fehler“: wenn die Blickgerade nach dem „Fehler“ nicht mehr außerhalb des Schwellwertwinkels gerät, bevor der Versuch terminiert.

Visual Paired Comparison

Ein Referenzmuster sowie ein zu vergleichendes Vergleichsmuster werden wiederholt der Probandin präsentiert, mit der Aufgabe, festzustellen, durch welche Bildinhalte sich die beiden Muster unterscheiden. Der Versuch wird durch die Probandin durch eine Stopptaste bei Erreichen einer Antwort terminiert. Folgende Merkmale werden erhoben, gemäß (Lagun et al., 2011):

- Neuigkeitspräferenz (NP). Prozentsatz der Blickdauer auf dem Vergleichsmuster relativ zur Gesamtblickdauer.
- Fixationsdauer (FD). Fixationsdauer insgesamt bis zum Terminieren der Aufgabe.
- Re-fixationen (RF). Mittlere Re-fixationstiefe bei der Durchführung der Aufgabe. Re-fixation (Fixationsbesuch) findet statt innerhalb einer Abweichung von 2° zwischen aktueller und vergangener Fixation. Die Re-fixationstiefe ist die Anzahl der Sakkaden zwischen aktueller und besuchter Fixation.
- Sakkadenorientierung (SO). Prozentsatz der „vertikalen Sakkadenwinkel“. Ein vertikaler Sakkadenwinkel ist ein Sakkadenwinkel innerhalb des Intervalls (90°± 7°). Ein Sakkadenwinkel ist der Winkel zwischen aktuellem und vorherigem Sakkadenvektor.
- Reaktionszeit (RT). Zeitintervall zwischen dem Beginn einer Vergleichsaufgabe und der Terminierung durch die Probandin.
- Fehlerrate (ER). Fehler bei der Klassifikation (falsches Detail als Fehler definiert).

Erhebung und Analyse der kognitiven Funktionen Tablet PC

Beim multimodalen spielerischen Training „DaheimAktiv“ werden am Tablet PC alle Interaktionen und Events gespeichert, mit einem anonymisierten Code für den Personenbezug versehen und schließlich in einer zentralen Datenbank für weitere Datenanalytik abgelegt. Die Resultate ermöglichen es, die Trainingsspiele und zugehörige Leistungsmerkmale den psychologischen Fragebögen quantitativ gegenüberzustellen und geeignete Korrelate zu identifizieren.

Im Software-Modul MIRA (Mobile Instrumental Review of Attention) kann die Augenbewegung der Nutzerin automatisiert über die Webkamera verfolgt werden. Die folgenden Module sind für die Datenerhebung und damit für weitere Analytik vorgesehen:

- Komponente „Trail Making Test”: Hier werden Zufallsmuster im Sinne des Tests „Trail Making Test“ A, B erzeugt und die Nutzerin kann in analoger jedoch spielerischer Weise (in der Form eines Videogames) aktiv sein, die Daten werden aufgezeichnet.
- Komponente „Go/Nogo“: Im Sinne eines go/nogo Tests werden Stimuli sequentiell angeboten und die Nutzerin wird entsprechende interaktive Artefakte setzen und für die weitere Analyse zur Verfügung stellen.
- Komponente “TAP-Konzentration”: im Sinne des “Test of Attentional Performance” wird die Konzentration bei einer abstrakten Aufgabe gemessen. Die Daten können über die zentrale Datenbank ausgewertet werden.

Lebensstilfaktoren Tablet PC

Mit dem Tablet PC werden zudem Lebensstilfaktoren für die Intensivierung der Alzheimer Demenz möglichst kontinuierlich, jedoch spielerisch (siehe „Pick-A-Mood“, Desmet et al., 2016) erhoben. Dabei sollen diese „Erhebungsmethoden“ bei steter Benützung zu „Serious Game“ Effekten beitragen, die die Benützung dieser Erhebungsmethoden weiterhin verstärkt. Den Nutzerinnen werden Erfolgstableaus in spielerischer, nicht oberflächlich kompetitiver Weise zugeordnet, beispielsweise ein Garten, wobei bei jedem Beitrag zu der Erhebung eine Blume, ein Baum zum Gedeihen angeregt werden, es entstehen so hübsche pflanzliche Gebilde. Die Erwartung der Nutzerin kann nun zu weiterem Spiel angeregt werden. Folgende Erhebungs-Komponenten sind geplant:

- „Soziale Aktivitäten“: Erhebung der täglichen sozialen Kontakte. Die Auswahl geschieht durch das Drücken eines von vielen Knöpfen, wobei jeder eine bestimmte soziale Intensität des Tages repräsentiert: „kein Mensch“, „1 Mensch auf einem Stuhl“; „2 Menschen“, usw. → Auswahl durch „Pick-A-Social State“.
- Piktorale Darstellung verschiedener Gemütszustände durch „Pick-A-Mood“ (siehe Anhang 2; Desmet et al., 2016).
- Eingabe von aktuellem Gewicht, Größe (konstant aus dem biographischen Datenteil) und Ausgabe des aktuellen BMI Wertes (graphisch, spielerisch).
- Eingabe von systolischem und diastolischem Blutdruckwert, wenn erhältlich (besondere „Belohnungswerte“ im Serious Game.
- Piktorale Darstellung verschiedener täglicher „Ernährungsform“.
- Piktorale Darstellung verschiedener „Schlafformen“.
- Piktorale Darstellung verschiedenen „Rauchverhaltens“.

Erhebungsmethoden - Leistungstests

MMSE

Der kognitive Status wird mit der deutschen Version des MMSE erhoben (Folstein et al., 1975). Der MMSE ist der weltweit am meistverwendete kognitive Test (Folstein et al., 1975, Mahlberg & Gutzmann, 2005, Hensel et al. 2007, Hensel et al., 2009) und die psychometrischen Gütekriterien sind ausgiebig getestet (Rösler et al., 2003, Mahlberg & Gutzmann, 2005, Hensel et al., 2007, Schramm et al., 2002, Kahle-Wrobleski et al., 2007). Der MMSE ermöglicht ein Screening der kognitiven Beeinträchtigung von großen Populationen (Folstein et al., 2010) und die Bestimmung des Grades der kognitiven Einschränkung (keine - schwere kognitiven Beeinträchtigung) ist möglich (Rösler et al., 2003).

Neuropsychologische Testbatterie (Harrison et al., 2007)

- **WMS III-R Figurale Paarerkennung inkl. verspäteter Abruf:** Bei diesem Subtest der Wechsler Memory Scjae III-R (Härting et al., 2000) soll der Proband 6 Strichfiguren, jeweils gepaart mit einer bestimmten Farbe, merken. Anschließend werden

die Strichfiguren alleine dargeboten und der Proband soll in dem Testheft auf die dazugehörigen Farben zeigen. Maximal können bei dieser Aufgabe 18 Punkte erreicht werden. Beim verspäteten Abruf werden dem Teilnehmer zu einem späteren Zeitpunkt die Figuren nochmals gezeigt und der Teilnehmer soll auf die entsprechende Farbe zeigen (maximale Punkteanzahl: 6 Punkte)

- **WMS III-R Verbale Paarerkennung inkl. Verspäteter Abruf:** Dieser Test ist das verbale Pendant zum vorigen Untertest. Dem Probanden werden 8 Wortpaare vorgelesen, von denen er jeweils das zweite Wort bei der anschließenden Vorgabe des jeweiligen ersten Wortes erinnern soll. Beim verspäteten Abruf werden dem Teilnehmer zu einem späteren Zeitpunkt die Worte erneut vorgelesen und der Teilnehmer muss das dazugehörige Wort nennen. Die maximale Punkteanzahl beträgt 24 bzw. 8 Punkte.
- **Verbaler Lern- und Merkfähigkeitstest (VLMT):** Der VLMT ist ein Test zum seriellen Listenlernen mit nachfolgender Distraktion, Abruf nach Distraktion und halbstündiger Verzögerung sowie einem Wiedererkennungsdurchgang. Das Testmaterial des VLMT besteht aus zwei Wortlisten, die sich aus je 15 semantisch unabhängigen Wörtern zusammensetzen, und einer Wiedererkennensliste, die die 30 Wörter der beiden Wortlisten sowie 20 weitere semantisch bzw. phonematisch ähnliche Distraktorwörter enthält. Mit dem VLMT können unterschiedliche Parameter des deklarativen Verbalgedächtnisses wie die Supraspanne, die Lernleistung, die langfristige Enkodierungs- bzw. Abrufleistung und die Wiedererkennungsleistung erfasst werden. Der VLMT stellt entspricht der englischen Version des Auditory Verbal Learning Test.
- **WMS III-R Zahlenspanne:** Dieser Subtest (Härting et al., 2000) besteht aus zwei Aufgabentypen, der Zahlenspanne vorwärts und der Zahlenspanne rückwärts. Bei der Zahlenspanne vorwärts werden dem Probanden Zahlenfolgen wachsender Länge vorgelesen, die er unmittelbar danach wiederholen soll. Die vorgesagten Zahlenspannen werden bei jedem Durchgang um eine Zahl länger bis zu acht Zahlen hintereinander. Jeder Durchgang beinhaltet zwei Folgen (zweimal eine 3er Zahlenspanne, zweimal eine 4er Zahlenspanne, usw.). Bei der Zahlenspanne rückwärts werden dem Probanden ebenfalls Zahlenfolgen wachsender Länge vorgele-

sen, die er diesmal in genau umgekehrter Reihenfolge repetieren soll. Die Zahlenspannen werden bei jedem Durchgang um eine Zahl länger bis zu sieben Zahlen hintereinander. Auch hier beinhaltet jeder Durchgang zwei Folgen. Wiederholt der Proband keine der zwei Folgen eines Durchgangs der Zahlenspanne vorwärts richtig, wird diese Zahlenfolge beendet und mit der Zahlenspanne rückwärts fortgefahren. Wiederholt der Proband auch hier keine der zwei Folgen eines Durchgangs richtig, wird der Subtest beendet. Für jede richtig repetierte Folge gibt es einen Punkt. Maximal können bei dieser Aufgabe 24 Punkte erreicht werden.

- **RWT**: Der RWT (Regensburger Wortflüssigkeitstest; Aschenbrenner et al. 2001) ist ein diagnostisches Verfahren zur Erfassung der Wortflüssigkeit, bei dem über einen Zeitraum von ein oder zwei Minuten Lösungen verbal generiert werden müssen. Es stehen Subtests zur formallexikalischen und zur kategoriellen Wortflüssigkeit zur Verfügung. Es werden Normwerte sowohl für eine Minute als auch für zwei Minuten Bearbeitungszeit mitgeteilt. Die Interraterreliabilität für alle Untertests beträgt r = .99. Die Retestreliabilität über drei Wochen variiert für die einzelnen Untertests zwischen r_{tt} = .72 und r_{tt} = .89. Der Test wurde an verschiedenen neurologischen und psychiatrischen Patientenstichproben validiert (Patienten mit Hirntumoren, Patienten nach zerebralen Infarkten, Patienten mit langjähriger Alkoholabhängigkeit, Patienten mit Major Depression). Die Ergebnisse der im Testhandbuch beschriebenen Validierungsstichproben bestätigen die große Bandbreite des Verfahrens und die hohe Sensitivität der einzelnen Untertests des RWT auf eindrucksvolle Weise.
- **LDST (Letter Digit Substitution Test):** Der LDST ist ein Substitutionsverfahren mit Zeitnehmung (Speed-Test, van der Elst, 2006). Substitutionstests sind sensitive für zerebrale Dysfunktionen und sind nichtspezifisch, wodurch sie ein breites Spektrum unterschiedlicher Prozesse erfassen. Diese Prozesse inkludieren die Integration komplexer neuropsychologischer Prozesse inklusive visuelles Scanning, mentale Flexibilität, Aufmerksamkeit, psychomotorische Geschwindigkeit und Informationsverarbeitungsgeschwindigkeit.

TAP (Testbatterie zur Aufmerksamkeitsprüfung)
Die TAP ist ein computergestütztes psychologisches Testsystem für den Bereich der Aufmerksamkeitsdiagnostik. Mit der Testbatterie zur Aufmerksamkeitsprüfung (TAP) können unterschiedliche Teilaspekte der Aufmerksamkeit bei Kindern und Erwachsenen überprüft werden (Zimmermann & Fimm; Version 2.3.1).

MoCA (Montreal Cognitive Assessment)
Der MoCA ist ein getestetes Screening- Instrument für den kognitiven Status. Es kann auch Veränderungen des globalen kognitiven Abbaus messen (Freitas et al. 2012, Costa et al. 2014). Mit dem MoCa werden Aufmerksamkeit und Konzentration, Exekutivfunktionen, Gedächtnis, Sprache, visuokonstruktive Fähigkeiten, konzeptuelles Denken, Rechnen und Orientierung erhoben. Das Instrument wurde bereits in 46 Sprachen übersetzt und wird in über 100 Ländern angewendet (Nasreddine 2018).

NPI (Neuropsychiatric Inventory) (Cummings, 1997)
Das Neuropsychiatric Inventory wurde 1994 von Cummings et al. in den USA entwickelt und wird angewendet, um neuropsychiatrische Symptome zu erfassen. Das NPI besteht aus zwölf Unterskalen, mit denen zehn Verhaltensweisen sowie zwei neurovegetative Zustände erfasst werden. Die Basis dieses Assessmentinstruments bildet ein strukturiertes Interview mit einer Informantin/einem Informanten. Jede Unterskala beinhaltet zuerst die Frage, ob ein bestimmtes Symptom vorhanden ist oder nicht. Wird diese Frage von der Informantin/dem Informanten mit Ja beantwortet, wird das jeweilige Symptom mithilfe einer Skala genauer eingeschätzt. Ist das Symptom nicht vorhanden, wird zum nächsten Symptomkomplex übergegangen. Die Pflegenden schätzen die Frequenz mithilfe einer Vier-Punkte-Skala und die Schwere mithilfe einer Drei-Punkte-Skala ein.

Erhebungsmethoden Imaging

MAGNETRESONANZTOMOGRAPHIE (MRT)
Die MRT dient im Rahmen dieser Studie zur Diagnosesicherung einer Demenz vom Alzheimertyp. Zur Minimierung der Heterogenität in der Stichprobe ist eine genaue Abgren-

zung zu anderen Erkrankungen notwendig bzw. muss sichergestellt sein, dass keine andere Ursache wie beispielsweise vaskuläre Läsionen, Tumore, Normaldruckhydrocephalus oder metabolische Enzephalopathien ursächlich für die Defizite sind. Die MRT ist obligatorisch in der Diagnosestellung.
Folgende Sequenzen mit einer Gesamtdauer von etwa 45 Minuten werden durchgeführt:

- 3D-EPI mit Multiecho: für R2* Mapping und QSM
- 3D-T2
- 3D-T1 Multiecho MPRAGE: für verbesserte Segmentierungen
- 3D FLAIR
- Diffusion 1.5 mm isotrop mit Multiband und mehreren b-Werten
- T2*-Sequenz für Microbleeds
- Resting state (funktionelles MRT)

BIOBANK

Die Biobank wird mit dem Ziel eingerichtet endogene, biologische Faktoren bei Alzheimerdemenz in Hinblick auf den klinischen Verlauf der Erkrankung zu untersuchen. Die Biobank besteht aus der Sammlung von DNA, RNA, Serum und Plasma Proben. Diese werden bei der Basisuntersuchung und nach 1.5 Jahren entnommen. Dies ermöglicht die Durchführung sowohl cross-sektionaler als auch longitudinaler Studien, um die Bedeutung von DNA Varianten, DNA Methylierungsprozessen bzw. Veränderungen in der Genexpression in Bezug auf Demenzerkrankung zu untersuchen. Die Blutproben werden an der Neurologischen Univ. Klinik entnommen und anonymisiert, mit Studiennummer versehen an das Labor der Klinik zur weiteren Bearbeitung weitergeleitet. Die DNA und RNA Extraktion bzw. die Archivierung und Verwaltung der Biobank erfolgt an der Neurologischen Univ. Klinik Graz entsprechend den Vorgaben des §68 des Gentechnikgesetzes. Die Proben werden in Aliquote portioniert, bei -80°C gelagert und für etwaige spätere Projekte bereitgestellt.
Insgesamt werden folgende Mengen entnommen:
Für das Routinelabor: 1x 8ml Serum Röhrchen, 1x 8ml Lithium-Heparin, 2x 3ml EDTA, 1x 3,5ml Coagulation Sodium Citrat. Für die genetischen Untersuchungen : 3x 6ml EDTA, 1x 8ml Serum, 1x RNA

GENANALYSEN

Für die Studie ist eine ApoE Genotypisierung erforderlich. Der ApoE4 Genotyp ist mit rascherer Progredienz vergesellschaftet, sodass wir nachweisen müssen, dass zwischen der Trainingsgruppe und der Vergleichsgruppe kein Ungleichgewicht in der Frequenz der ApoE4 Träger besteht.

Da DNA Extraktion erforderlich ist wollen wir die Gelegenheit benutzen und die Patienten befragen ob sie für etwaige zukünftige wissenschaftliche Projekte die Einwilligung für weitere Verwendung der DNA geben. Es ist klar dass jedes dieser etwaigen Zukunftsprojekte eines separaten Ethikantrages bedarf.

Sämtliche Proben werden in anonymisierter Form ohne Namen mit einem Code versehen, versendet und bearbeitet. Die Proben können nur an der Universitätsklinik für Neurologie mit dem Namen des Probenspenders in Verbindung gebracht werden.

Sollten Ergebnisse von Genanalysen aber auch die Phänotypdaten im Rahmen wissenschaftlicher Kooperationen verwendet werden wird sicher gestellt dass der Probenspender für die kooperierenden Zentren nicht bestimmbar ist. Falls solche internationale Kooperationen angestrebt werden sind diese spezifisch bei der Ethikkommission, wie oben beschrieben, einzureichen.

An der Klinik für Neurologie werden derzeit im Rahmen eines Next-Generation Sequencing Panels beispielhaft folgende Risikogene für Alzheimerdemenz untersucht

ASNA1, ATP13A2, ATP1A3, ATP6AP2, C19orf12, CHCHD2, COMT, DCTN1, DNAJC13, DNAJC6, EIF4G1, FBXO7, FMR1, FTL, GBA, GCH1, GRN, HTRA2, LRRK2, MAPT, PANK2, PARK2, PARK7, PDE8B, PDE10A, PINK1, PLA2G6, PODXL, POLG, PRKAR1B, PRKRA, PTEN, RAB29, RAB39B, SLC30A10, SLC6A3, SNCA, SPG11, SPR, SYNJ1, TAF1, TENM4, TH, VPS13C, VPS35, ZFYVE26.

Eine entsprechende Untersuchung ist möglich aber von entsprechender Finanzierbarkeit abhängig.

AES (Apathy Evaluation Scale)

Die Apathie kann als Motivationsverlust begriffen werden, die sich auf den Ebenen Kognition, (beobachtbares) Verhalten und Emotion/Affekt abbildet. Die interne Konsistenz der deutschsprachigen Übersetzung ist vergleichbar mit dem Original (Cronbach’s a = 0,86

im Original vs. a = 0,92 in der Übersetzung). Es zeigt sich eine gute Retest−Reliabilität und Interrrater−Reliabilitäten. Ergebnisse zur Konstruktvalidität zeigten, dass die Skala signifikant mit der Apathieskala des NPI korrelierte (Lueken et al. 2006).

DEMQOL (Dementia Quality of Life)
Das Instrument zielt darauf ab, die gesundheitsbezogene Lebensqualität von Personen mit einer leichten bis mittelgradigen Demenz zu erfassen. Das Instrument besteht aus zwei interview-gestützten Versionen: „DEMQOL-Self“ für die Selbsterfassung der Lebensqualität der Personen mit Demenz und „DEMQOL-Proxy“ für die Fremdeinschätzung der Lebensqualität von Personen mit Demenz durch die Angehörigen (Bowling et al. 2015, Smith et al. 2005). Erhoben werden die Häufigkeit der Erfahrung mit bestimmten Emotionen und Aspekte der Gedächtnisfunktion im Alltag im Laufe der vergangenen Woche (Berwig et al. 2011). Beide Versionen des Instrumentes sind auf psychometrische Eigenschaften getestet (Berwig et al. 2009, Berwig et al. 2011).

FAM
Der „Fragebogen zur Erfassung aktueller Motivation in Lern- und Leistungssituationen (FAM, siehe Anhang 3) wurde von Rheinberg et al. (2001) entwickelt und ist ein Fragebogen, der mit 18 Items vier Komponenten der aktuellen Motivation in (experimentellen) Lern- und Leistungssituationen erfasst: Misserfolgsbefürchtung, Erfolgswahrscheinlichkeit, Interesse und Herausforderung. Die deutsche sowie eine amerikanische Version weisen zufriedenstellende Konsistenzen auf (6 Stichproben, N=944). Aus verschiedenen Experimenten liegen bereits Validitätshinweise dazu vor, dass die vorweg erfassten Motivationskomponenten mit dem nachfolgenden Lernverhalten und der Lernleistung im Zusammenhang stehen. „Lewin (1946) folgend wurde angenommen, dass sich Verhaltenstendenzen stets nur aus der Wechselwirkung zwischen Person- und Situationsfaktoren ergeben. In der Motivationspsychologie bezeichnet man solche Personfaktoren bekanntlich als Motive. Sie werden als überdauernde, hochgeneralisierte Merkmale der Person aufgefasst, bestimmte Klassen von Anreizen zu bevorzugen. Situationsfaktoren sind die situativen Anregungsgehalte, also die motivspezifischen Befriedigungschancen, die die gegebene Situation in Aussicht stellen kann. Passen diese Anregungsgehalte zur Motiv-

struktur der Person, resultiert die aktuelle Motivation mit ihren ausrichtenden und energetisierenden Verhaltenskonsequenzen. Erst diese aktuelle Motivation – und nicht etwa die Motive – haben direkten Einfluss auf das Verhalten.“ (Rheinberg et al., 2001).

Fantastic Lifestyle Checklist

Das Fantastic Lifestyle Checklist wurde von Wilson et al. (1984) entwickelt und ist ein überprüftes Instrument, welches Lebensstilfaktoren von Personen erfasst. Das Instrument hat 25 Fragen in den 9 folgenden Bereichen: 1) Familie und Freunde, 2) körperliche Aktivität, 3) Ernährung, 4) Tabak und Giftstoffe, 5) Alkoholkonsum, 6) Schlaf, Sicherheitsgurt, Stress und Safer Sex, 7) Verhaltensmuster, 8) Einsicht und 9) Karriere.

Fitness Tracker zur Erhebung und Analyse der Mobilität

Zur Erhebung der Mobilitätsdaten wird ein Fitness Tracker eingesetzt. Die so gemessenen Daten liefern Informationen zu Aktivitäten (z. B. Schrittzähler) bzw. Motivation zur Erhöhung des eigenen Bewegungsradius. Logdaten des Fitness Trackers können über die Fitness Tracker App des Herstellers nur von den Teilnehmern eingesehen werden. Es erfolgt keine Übertragung auf die Systeme des Konsortiums.

Der Einsatz eines Fitness Trackers ermöglicht Aktivitätsmonitoring und Motivation zur Teilnahme an Bewegungseinheiten, die eine Vergrößerung des persönlichen Bewegungsradius bewirken sollen.

GDS (The Geriatric Depression Scale)

Diese Skala misst depressive Störungen bei älteren Menschen und ist eines der meist eingesetzten Instrumente, die auch bei Personen mit Demenz verwendet wird (Sheehan et a. 2012). Die Skala ist auf psychometrische Eigenschaften getestet (Allgaier et al., 2011, Gauggel et al. 1999, Sheehan et al. 2012).

Interviews (Einzelinterviews und Fokusgruppen)

Diese beziehen sich auf vertiefende Fragen zu dem quantitativen Fragebogen TUI. Es werden Einzelinterviews mit den Personen mit Demenz durchgeführt sowie Fokusgruppen mit den anderen Zielgruppen (Angehörigen, Pflegepersonen und M.A.S. TrainerIn-

nen) mit min. 3-4 TeilnehmerInnen pro Gruppe. Die Interviews werden durch interviewerfahrene, geschulte MitarbeiterInnen (z. B. PsychologInnen, PädagogInnen) des Sozialverein Deutschlandsberg (SVDL) und der MUG-Pflege durchgeführt. Die Interviews werden in ruhiger Umgebung im häuslichen Umfeld oder im SVDL durchgeführt und per Smartphone oder Tablet aufgenommen. Die Interviews werden danach vom SVDL an das Institut für Pflegewissenschaft über den gesicherten Server des Joanneum Research zur Transkription und Analyse übergeben.

PAS (Pflegeabhängigkeitsskala)

Die Pflegeabhängigkeit wird mit der deutschen Version der Pflegeabhängigkeitsskala (PAS) erhoben (Dijkstra et al. 1996, Lohrmann 2003). Die PAS enthält 15 Elemente mit denen physische und psychosoziale Aspekte durch eine 5-Punkte-Likert-Skala (von völlig abhängig bis unabhängig) bewertet werden. Es können Werte zwischen 15 (völlig abhängig) und 75 (völlig unabhängig) Punkten erzielt werden (Lohrmann et al. 2003). Das Instrument wurde sehr gut auf psychometrische Gütekriterien getestet (Dijkstra 1998, Lohrmann 2003).

PSS-10 (Perceived Stress Scale)

Die von Cohen, Kamarck und Mermelstein entwickelte Perceived Stress Scale (PSS) (Cohen et al. 1983) ist ein etablierter Fragebogen zur Selbsterfassung von wahrgenommenen Stressempfinden. Auf einer 5-Punkte-Likert-Skala wird gemessen, inwieweit das Leben im letzten Monat als unvorhersehbar, unkontrollierbar und überlastet erlebt wurde (0 = „nie“, 1 = „fast nie“, 2 = „manchmal“, 3 = “ ziemlich oft ”, 4 =” sehr oft ”) (Cohen et al. 1983, Klein et al. 2016). Höhere Werte deuten auf einen höheren Grad an wahrgenommenem Stress hin (Cohen et al. 1983, Klein et al. 2016). Die ursprüngliche Scala besteht aus 14 Items (PSS-14), jedoch wird die Kurzversion der PSS (PSS-10) aufgrund von verbesserten psychometrischen Eigenschaften für die klinische Forschung empfohlen (Lee 2012). Die deutsche Version der PSS-10 zeigt ebenfalls eine gute interne Konsistenz auf (Cronbach α=0.84) (Klein et al. 2016).

TUG (Timed UP and GO Test)

Verfahren zur Beurteilung des Körpergleichgewichts/Mobilität und des Sturzrisikos bei einer alltäglichen Bewegungsaufgabe. Die Person sitzt bequem in einem Stuhl mit Armlehnen, der 3 m von einem definierten Ziel steht. Nach Aufforderung soll die Person aufstehen, einen Moment vor dem Stuhl verweilen, zu der 3 m entfernten definierten Stelle gehen, sich umdrehen ohne etwas zu berühren, zum Stuhl zurückgehen, sich erneut umdrehen und wieder auf dem Stuhl platznehmen (Podsiadlo et al. 1991). Die psychometrischen Eigenschaften sind überprüft (z. B. Interrater Reliabilität: ICC = .99 (3 Untersucher, n = 22); Validität: Timed "Up and Go" vs. Berg Balance Scale r = -.81) (IQPR 2012; Bossers et al. 2012). Der Test ist für Personen mit Demenz empfohlen (Bossers et al. 2012).

TMT A/B (Trail Making Test A/B)

Der „Trail Making Test“ ist ein neuropsychologischer Test, der die visuelle Aufmerksamkeit und die Umstellfähigkeit misst. In der Aufgabe müssen 25 Punkte verbunden werden. Zwei Versionen sind erhältlich: A, in welcher nur Nummern verbunden werden müssen (1,2,3,4,) und B, in welcher abwechslungsweise Nummern und Buchstaben verbunden werden müssen (1, A, 2, B, 3, C,…). Das Ziel ist es, den Test möglichst schnell zu beenden. Die gemessene Zeit dient als Resultat.

Affective Slider

Die Erhebung momentaner affektiver Zustände („Emotionen“) ist von zentraler Bedeutung für die Erhebung der affektiven Komponente im Zusammenhang mit den Leistungsdaten durch die Benützung des Tablet PCs („Multimodale Aktivierung“ App, MMA). Zur Erhebung im Sinne der Selbsteinschätzung dient nach wissenschaftlichen Gesichtspunkten der „Affective Slider“ („AFSL“; Betella & Verschure, 2016). Dieses Erhebungstool benötigt lediglich einen Fingerdruck pro Skala auf dem drucksensitiven Tablet PC. Präsentiert wird zur Nutzer-Eingabe einerseits die Skala für Bewertung der aktuellen Stimmung („pleasure“; gut-schlecht), andererseits die Skala für die Bewertung der Erregung („arousal“; ruhig-aufgeregt). Aus diesen zwei Messdimensionen lassen sich gemäß Russel’s bipolarer Raumdarstellung (Russel et al., 1999) alle grundlegenden Emotionen darstellen.

Der AFSL wird jedes Mal aktiviert, wenn die/er Nutzer/in am Home Screen der MMA App den Button für „MIRA" (Mobile Instrumental Review of Attention) betätigt, um die zugehörigen Komponenten der Aufmerksamkeits- und kognitiven Spiele durchzuführen. Durch die gleichzeitige Datum- und Zeitabfrage wird eine zweimalige Auslösung des AFSL verhindert, um eine Sättigung der Motivation der Nutzer/innen zu verhindern. In der Analyse der Studiendaten werden die Emotionsdaten sodann mit den Leistungsdaten aus der Interaktion mit anderen Komponenten der MMA App korreliert, um Ableitungen über die Funktionalität der affektiven Zustände im Kontext kognitiver Leistungen im Alltagsbereich der Demenzbetroffenen durchführen zu können.

TUI (Technology Usage Inventory)
Das TUI dient zur Erfassung von technologiespezifischen und psychologischen Faktoren, die zur tatsächlichen Verwendung einer Technologie beitragen. Es enthält insgesamt 30 Items aufgeteilt auf 8 Skalen. Diese inkludieren Neugierde, Ängstlichkeit, Interesse, Benutzerfreundlichkeit, Immersion, Nützlichkeit, Skepsis und Zugänglichkeit. Zusätzlich enthält das Verfahren die Skala Intention to Use (ITU). Die internen Konsistenzen (Cronbachs Alpha) der acht Skalen können insgesamt als gut bewertet werden. Sie bewegen sich im Rahmen von $\alpha = .70$ bis $\alpha = .89$. Eine Faktorenanalyse ergab eine 8-faktorielle Struktur des TUI. Zusätzlich liegen Hinweise zur psychophysiologischen Validierung einzelner TUI-Skalen vor (Kothgassner et al. 2013).

WHOQOL-100 (World Health Organization Quality of Life Scale)
Der WHOQOL-100 ist ein Instrument zur Erfassung der subjektiven Lebensqualität. Grundlage des Instruments ist die Definition von Lebensqualität als die individuelle Wahrnehmung der eigenen Lebenssituation im Kontext der jeweiligen Kultur und des jeweiligen Wertesystems sowie in Bezug auf persönliche Ziele, Erwartungen, Beurteilungsmaßstäbe und Interessen. Der WHOQOL-100 umfasst insgesamt 100 Items, die den Dimensionen physisches Wohlbefinden, psychisches Wohlbefinden, Unabhängigkeit, soziale Beziehungen, Umwelt und Religion/Spiritualität zugeordnet sind. Der Fragebogen diskriminiert sehr gut zwischen Personen mit gesundheitlichen Beeinträchtigungen und gesunden Personen sowie zwischen Personen mit physischen und Personen

mit psychischen Erkrankungen. Die interne Konsistenz (Cronbachs Alpha) der Subskalen des WHOQOL-100 liegt zwischen α = .59 und α = .91. Für den WHOQOL-100 (N = 715) liegen altersgestaffelte Referenzwerte für den Altersbereich 18 bis über 85 Jahre vor (The WHOQOL Group 1998).

DAD (Disability Assessment for Dementia)

Die "Disability Assessment for Dementia Skala" ist eine Skala zur Messung der funktionellen Fähigkeiten in Aktivitäten des Täglichen Lebens (activities of daily living, ADL) bei Personen mit kognitiven Beeinträchtigungen wie Demenz. Grundlegende und instrumentelle Aktivitäten des täglichen Lebens werden in Relation zu exekutiven Fähigkeiten untersucht. Der DAD misst grundlegende Aktivitäten des täglichen Lebens, instrumentelle Aktivitäten des täglichen Lebens, Freizeitaktivitäten, Initiierung von Aktionen, Planung und Organisation, und effektive Beendigung von Aktivitäten. Die Test-Re-test Reliabilität liegt bei ICC=.96, die inter-Rater Reliabilität bei ICC=.95.

ZBI (Zarit Burden Interview)

Das ZBI ist das am häufigsten eingesetzte Instrument zur Erfassung der subjektiven Belastung von Angehörigen von Personen mit Demenz. Das Instrument weist hohe psychometrische Eigenschaften auf (Cronbachs α=0,91) sowie gute Korrelationen als Validitätshinweis in Bezug auf Wohlbefinden des Angehörigen, Pflegeabhängigkeit, und neuropsychiatrische Symptome der Person mit Demenz (Braun et al. 2010).

PAL (Pool Activity Level)

Das PAL-Instrument erfasst den Aktivitätslevel einer Person bei den Aktivitäten des täglichen Lebens. Es umfasst neun Aktivitätsbereiche. Dazu gehören unter anderem Waschen, Anziehen, Essen oder Kommunikationsfähigkeiten. Mithilfe der Items lässt sich festhalten, inwiefern sich eine Person zum Beispiel beim Anziehen beteiligen kann: „Braucht Hilfe beim Planen, was angezogen werden soll, aber erkennt Kleidungsstücke und weiß, wie man sie anzieht; braucht Hilfe bei der Reihenfolge des Anziehens". Mit den Antworten lässt sich ein Aktivitätslevel bestimmen, das sowohl über Fähigkeiten als auch nötige Hilfestellungen informiert. Es ist ein überprüftes Instrument für Personen mit Demenz (Tatzer & Pool 2018).

VAMS™ (Visual Analog Mood Scales)
Das VAMS ist ein reliables und valides Messinstrument für acht spezifische Stimmungszustände (Angst, Verwirrt, Traurig, Wütend, Energetisch, Müde, Glücklich und Angespannt). VAMS stellt minimale kognitive oder sprachliche Anforderungen an den Befragten und eignet sich besonders für neurologisch beeinträchtigte Personen oder für Personen, die verbal oder kognitiv anspruchsvolle Instrumente nicht ausführen können. Die Punktzahl für jede Stimmung reicht von 0 bis 100, wobei 100 ein maximales Niveau dieser Stimmung darstellt und Null ein minimales Niveau (oder Fehlen) dieser Stimmung (PAR 2019).

4.5. Statistik

Quantitativer Teil
Die Daten werden mit IMB SPSS Version 24 ausgewertet. Das Signifikanzniveau wird auf alpha=0.05 festgelegt. Deskriptive Statistiken der Daten werden je nach Art der Verteilung als Mittelwert und Standardabweichung oder als Median und Quartile dargestellt. Zur Beschreibung von kategorialen Daten werden absolute und relative Häufigkeiten herangezogen. Zur Beantwortung der primären Fragestellung, ob das Tablet basierte Training einen positiven Effekt auf die Kognition hat, werden sowohl der primäre, die Leistung in der neuropsychologischen Testbatterie (NTB, globaler Score), als auch die sekundären Outcome Parameter (Sub-Tests der NTB, alle anderen neuropsychologischen Assessments) zwischen der IG und der KG mittels ANCOVA verglichen (adjustiert für multiple Vergleiche). Des Weiteren wird das Gesamtgehirnvolumen, das Volumen der Hirnlappen sowie das Hippocampus-Volumen, die zerebrale Mikrostruktur und die funktionale Konnektivität gemessen und zwischen den Gruppen verglichen. Weitere sekundäre Outcome Parameter werden ebenfalls mittels ANCOVA zwischen den Gruppen verglichen. Prä-post-Vergleiche (ANOVA-repeated measures) sollen Aufschluss über die Veränderungen über die Zeit liefern. Die Analysen werden für Alter, Geschlecht, Komorbiditäten und Bildung adjustiert.

Qualitativer Teil

Die qualitativen Interviews werden im Softwareprogramm MAXQDA organisiert, codiert und mittels qualitativer Inhaltanalyse (Kimberly & Neuendorf 2017) vom Institut für Pflegewissenschaft ausgewertet.

4.6. Ethische Aspekte

4.6.1. Informierte Zustimmung

Es werden nur Personen inkludiert, die eine zuvor informierte schriftliche Einverständnis geben. Beziehungsweise bei Personen mit Demenz, welche einen Angehörigen mit Vertretungsbefugnis oder Sachwalter haben, wird die informierte schriftliche Einverständniserklärung bei diesen Personen eingeholt. Alle TeilnehmerInnen können jederzeit ohne Angabe von Gründen die Teilnahme an der Studie verweigern oder beenden. Sämtliche Untersuchungen sind mit Ausnahme der Blutabnahme nicht invasiv. Weiters werden sämtliche Untersuchungen, einschließlich des 3T MR in der klinischen Routine eingesetzt. Die genetischen Untersuchungen werden entsprechend der gesetzlichen Vorgaben durchgeführt. Die Genanalysen dienen rein wissenschaftlichen Zwecken, werden nur nach schriftlicher Zustimmung des Probenspenders durchgeführt, sämtliche Proben werden in anonymisierter Form ohne Namen mit einem Code versehen bearbeitet. Die Proben können nur an der Univ. Klinik für Neurologie mit dem Namen des Probenspenders in Verbindung gebracht werden.

Sollten Ergebnisse aus der Genanalyse vernetzt werden, so stellen wir sicher, dass der Probenspender für die kooperierender Zentren nicht bestimmbar ist. Mit Ausnahme der Blutabnahme sind keine Schädigungen oder Belastungen für den Teilnehmer zu erwarten. Das Nutzen-Risiko-Verhältnis ist damit hoch einzustufen.

4.6.2. Datenschutz

4.6.2.1. Datenschutz Fragebögen, Interviews

Jedem Studienteilnehmer wird bei Studieneinschluss bzw. Randomisierung ein individueller anonymisierter Code an der UK für Neurologie zugewiesen und an den SVDL und

an das RK übermittelt. Die Kommunikation zwischen den Projektpartnern bzw. jeglicher Datentransfer wird mittels dieses Codes passieren um die Anonymität der TeilnehmerInnen zu gewährleisten. Die Fragebögen und Interviews (Einzelinterviews und Fokusgruppen) werden über einen gesicherten Server (Passwortgeschützt) der MUG vom Sozialverein Deutschlandsberg an die MUG übergeben. Alle personenbezogenen Daten der TeilnehmerInnen werden vertraulich behandelt und Interviews während der Transkription anonymisiert. Die Univ. Klinik für Neurologie - Klinische Abteilung für Neurogeriatrie, das Institut für Pflegewissenschaft MUG und Joanneum Research Digital (Forschungspartner) haben Zugriff auf die Daten für Eingabe und Analyse.

4.6.2.2. Datenschutz, Privatsphäre „DaheimAktiv“

„DaheimAktiv“

Login/Backend „DaheimAktiv“

Damit „DaheimAktiv“ verwendet werden kann, ist es notwendig sich mit einer E-Mail-Adresse und einem Passwort zu registrieren, welches verschlüsselt in der Datenbank gespeichert wird. Es kann pro Account immer nur ein Gerät (Tablet) aktiv verwendet werden, welches dann auch an diesen Zugang gebunden ist. Dafür ist es notwendig auch die eindeutige Seriennummer des Geräts zu speichern und somit mit dem Benutzer zu koppeln. Weitere personenbezogene Daten sind optional und daher nicht verpflichtend einzugeben.

Absolvierte Einheiten „DaheimAktiv“

„DaheimAktiv“ speichert zu jeder absolvierten Einheit einen Datenstream ab, der die Leistung der TeilnehmerInnen während der absolvierten Einheiten widerspiegeln soll. Hier geht es um Geschwindigkeit und Präzision bei den inkludierten Übungsprogrammen (z. B. Fehlersuchbilder, falsche/richtige Antworten, Quizze). Diese Daten sind notwendig um eine Verbesserung/Verschlechterung der TeilnehmerInnen zu erkennen, um Bereiche herauszufinden, in denen TeilnehmerInnen sehr gut sind und um festzustellen wo es noch Verbesserungspotential gibt. Für allgemeine Analysen dieser Zielgruppe werden die Daten anonymisiert.

4.6.2.3. Datenschutz Fitness Tracker

Die Verwendung des Fitness Trackers ist gebunden an einen Benutzeraccount, der über die Anwendung des Fitness Trackers vom Benutzer angelegt werden muss. Es erfolgt keine Datenübertragung der Logdaten in die zentrale Datenbank des Projektes. Die Daten können nur vom Benutzer selbst unter Angabe seiner Benutzungsdaten ausgelesen werden.

4.7. Nutzen/Risiken

4.7.1. Nutzen

Die Anwendung des multimodalen „DaheimAktiv“ Serious Games am Tablet PC als nicht-pharmakologische Intervention fördert psychische, kognitive, physische und soziale Fähigkeiten. Der Nutzen könnte auf lange Sicht in einer Stabilisierung des Krankheitsverlaufes liegen. Die Ergebnisse der TeilnehmerInnen unterstützen die Weiterentwicklung von kognitiven Trainingsprogrammen am Tablet PC für die Anwendung in der Pflegepraxis. Insbesondere die TeilnehmerInnen mit Demenz liefern wertvolle Erkenntnisse für die individuellen Bedürfnisse ihrer eigenen Zielgruppe. Laut des Systematik Reviews von Span et al. (2013) sollen Personen mit Demenz in allen Phasen eines Entwicklungsprojektes mitintegriert werden, damit wertvolle, benutzerfreundliche, unterstützende Technologien entwickelt werden können, welche die Lebensqualität dieser Zielgruppe erhöhen und ein längeres Verbleiben im eigenen Zuhause ermöglichen.

4.7.2. Risiken

Psychische (z. B. Überlastung) oder körperliche (z. B. Ermüdung) Belastungen durch die Anwendung von Tablet-PC Programm „DaheimAktiv“ können eventuell auftreten. Um Belastungen zu minimieren, werden die Trainingsprogramme auf die Personen mit Demenz abgestimmt (z. B. Schwierigkeitsgrad, Zeitdauer) und die Personen werden von DemenztrainerInnen während der Studie begleitet und unterstützt. Es kann auch jederzeit Kontakt mit dem Forschungsteam aufgenommen werden um Probleme zu besprechen. Während der bildgebenden Untersuchung mittels MRT kann Unbehagen bei den

TeilnehmerInnen auftreten. Jede/r TeilnehmerIn kann die Untersuchung auf Wunsch sofort abbrechen. Bezüglich der Blutabnahme besteht ein geringes Risiko für Blutergüsse, Blutungen oder Schmerzen an der Einstichstelle und in seltenen Fällen das Risiko einer Ohnmacht bei der Blutabnahme. Teilnehmern mit Blutungsneigung oder bekannter Neigung für Kollaps wird von einer Teilnahme abgeraten. Bezüglich der Lumbalpunktion besteht das Risiko von Blutungen und Blutergüssen, Infektionen und Entzündungen, Kreislauf- und Bewusstseinsstörungen sowie vorübergehende Nervenausfälle mit Taubheitsgefühl oder Lähmungen. Es wird bei der Lumbalpunktion ausdrücklich darauf hingewiesen, dass diese optional durchgeführt wird.
Die Teilnahme an der Studie kann jederzeit ohne Angabe von Gründen und ohne Nachteile für den Patienten/die Patientin oder den Angehörigen beendet werden.

Referenzliste

(ADI, 2013a) World Alzheimer Report 2013: Journey of caring: an analysis of long-term care for dementia. ADI, London.

(Allgaier et al., 2011) Validität der Geriatrischen Depressionsskala bei Altenheimbewohnern: Vergleich von GDS-15, GDS-8 und GDS-4. Psychiat Prax, 38:280–286.

(Aschenbrenner et al. 2001) Regensburger Wortflüssigkeitstest. 1. Auflage. Hogrefe, Göttingen.

(Berwig et al., 2009) Critical evaluation of self-rated quality of life in mild cognitive impairment and alzheimers disease – further evidence for the impact of anosognosia and global cognitive impairment. The Journal of Nutrition, Health & Aging, 13(3):226-230.

(Berwig et al., 2011) Self-related quality of life in mild cognitive impairment and alzheimers disease. GeroPsych, 24(1):45-51.

(Betella & Verschure, 2016) The Affective Slider: A Digital Self-Assessment Scale for the Measurement of Human Emotions. PLoS ONE 11(2):e0148037.

(Boman et al., 2014) Exploring the usability of a videophone mock-up for persons with dementia and their significant others. BMC Geriatr 14, 49. https://doi.org/10.1186/1471-2318-14-49.

(Bowling et al., 2015) Quality of life in dementia: a systematically conducted narrative review of dementia-specific measurement scales. Aging & Mental Health, 19(1):13-31.

(Braunseis et al., 2012) The risk for nursing home admission did not change in ten years-a prospective chohort study with five-year follow-up. Arch Gerontol Geriatr 54:e63-e67.

(Cao et al., 2017) Realtime Multi-Person 2D Pose Estimation using Part Affinity Fields, Proc. CVPR 2017.

(Chalfont et al., 2018) A mixed methods systematic review of multimodal non-pharmacological interventions to improve cognition for people with XVII dementia, Dementia (London). 19(4):1086-1130. doi: 10.1177/1471301218795289.

(Cohen et al., 1983) A global measure of perceived stress. Journal of Health and Social Behaviour, 24:385-396.

(Cummings, 1997) The Neuropsychiatric Inventory. Neurology, vol. 48, suppl. 6, pp 10-16.

(Desmet et al., 2016) Mood Measurement with Pick-A-Mood; Review of current methods and design of a pictorial self-report scale. *J. Design Research*, vol. 14, no. 3.

(Dijkstra et al., 1996) Nursing-care dependency. Development of an assessment scale for demented and mentally handicapped patients", Scandinavian Journal of Caring Sciences, vol. 10, no. 3, pp. 137-143.

(Dijkstra et al., 1998) Operationalization of the concept of 'nursing care dependency' for use in long-term care facilities. Aust N Z J Ment Health Nurs 7(4):142-51.

(Djabelkhir et al., 2017) Computerized cognitive stimulation and engagement programs in older adults with mild cognitive impairment: comparing feasibility, acceptability, and cognitive and psychosocial effects, Clinical Interventions in Aging, vol. 12, pp. 1967-1975.

(Ehret et al., 2015) Technikbasiertes Spiel von Tagespflegebesuchern mit und ohne Demenz, Zeitschrift für Gerontologie und Geriatrie, vol. 50, no. 1, pp. 35-44.

(Fasilis et al., 2018) A pilot study and brief overview of rehabilitation via virtual environment in patients suffering from dementia, Psychiatriki, vol. 29, no. 1, pp. 42-51.

(Folstein et al., 1975) Mini-mental state. A practical method for grading the cognitive state of patients for the clinician. J Psychiatr Res 12:189-198.

(Folstein et al., 2010) MMSE-2 Manual zur Durchführung und Auswertung [MMSE-2 user's manual]. PAR, Florida Ave.

(Garcia-Casal et al., 2017) Computer-based cognitive interventions for people living with dementia: a systematic literature review and meta-analysis, Aging Ment Health, vol. 21, no. 5, pp. 454-467.

(Gauggel et al., 1999) Validität und Reliabilität einer deutschen Version der Geriatrischen Depressionsskala (GDS), Zeitschrift für Klinische Psychologie und Psychotherapie, 28, pp. 18-27.

(Harrison et al., 2007) A Neuropsychological Test Battery for use in Alzheimer's disease clinical trials, Archives of Neurology, vol 64, no. 9, pp. 1323-1329.

(Härting, et al., 2000) Wechsler Memory Scale – Revised Edition, German Edition. Manual. Huber, Bern 2000.

(Hensel et al., 2007) Measuring cognitive change in older adults: reliable change indices for the Mini-Mental State Examination, Journal of Neurology, Neurosurgery & Psychiatry, vol. 78, no. 12, pp. 1298-1303.

(Hensel et al., 2009) Does a reliable decline in Mini Mental State Examination total score predict dementia? Diagnostic accuracy of two reliable change indices, Dementia and Geriatric Cognitive Disorders, vol. 27, no. 1, pp. 50-58.

(Hitch et al., 2017) Use of touchscreen tablet technology by people with dementia in homes: A scoping review, Journal of Rehabilitation and Assistive Technologies Engineering, vol. 4.

(IQPR, 2010) TGUG / TUG, Timed Get-Up and Go Test / Timed "Up and Go"-Test. Verfügbar unter: http://www.assessment-info.de/assessment/seiten/datenbank/vollanzeige/vollanzeige-de.asp?vid=370 (19.05.2018).

(Joddrell et al. 2016) Studies Involving People With Dementia and Touchscreen Technology: A Literature. JMIR Rehabil Assist Technol 2016;3(2):e10) doi:10.2196/rehab.5788 Review.

(Kahle-Wrobleski et al., 2007) Sensitivity and Specificity of the Mini-Mental State Examination for Identifying Dementia in the Oldest-Old: The 901 Study, Journal of the American Geriatrics Society, vol.55, no. 2, pp. 284–289.

(Kaufmann et al., 2010) Antisaccades: A probe into the dorsolateral prefrontal cortex in alzheimer's disease. a critical review, Journal of Alzheimer's Disease 19 (2010) 781–793.

(Klein et al., 2016) The German version of the perceived stress scale – psychometric characteristics in a representative German community sample. BMC Psychiatry, vol 16, no. 159.

(Klimova & Maresova, 2017) Computer-based training programs for older people with mild cognitive impairment and/or dementia', Frontiers in Human Neuroscience, vol. 11 (no pagination), no. 262.

(Kothgassner et al., 2013) TUI Technology Usage Inventory. ICARUS (Information- and **(PAR, 2019)** VAMS™ - Visual Analog Mood Scales™ by Stern. https://www.parinc.com/Products/Pkey/471, Accessed 02.07. 2019

(Lagun et al., 2011) Lagun, D., Manzanares, C., Zola, S.M., Buffalo, E.A., Agichtein, E. (2011). Detecting cognitive impairments by eye movement analysis using automatic classification algorithms, Journal of Neuroscience Methods, pp. 196-203.

(Lauriks et al., 2007) Review of ICT-based services for identified unmet needs in people with dementia. Ageing Research Reviews, 6(3):223-46.

(Lee, 2012) Review of the psychometric evidence of the perceived stress scale. Asian Nursing Research, vol. 6, no. 4, pp. 121-127.

(Lewin, 1946) Lewin, K. (1946). Action research and minority problems. *Journal of Social Issues*, 2, 34-46.

(Lim et. al., 2012) Usability of tablet computers by people with early-stage dementia. Gerontology. 2013;59(2):174-82.

(Lohrmann et al., 2003) “Care dependency:testing the German version of the care dependency scale in nursing homes and on geriatric wards”, Scandinavian Journal of Caring Sciences, vol. 17, pp. 51-56.

(Lohrmann, 2003) Die Pflegeabhängigkeitsskala: ein Einschätzungsinstrument für Heime und Kliniken - Eine methodologische Studie, In Department of Nursing Science Center for Humanities and Health Science, Charité-Universitätsmedizin Berlin, Berlin.

(Lueken et al., 2006) Die Apathy Evaluation Scale: Erste Ergebnisse zu den psychometrischen Eigenschaften einer deutschsprachigen Übersetzung der Skala. Fortschr Neurol Psychiat, 74; 714 – 722.

(Mahlberg & Gutzmann, 2005) "Diagnostik von Demenzerkrankungen", Deutsches Ärzteblatt, vol. 102, no. 28-29.

(McKhann et al., 2011) The diagnosis of dementia due to Alzheimer's disease: recommendations from the National Institute on Aging-Alzheimer's Association workgroups on diagnostic guidelines for Alzheimer's disease. Alzheimers Dement, 7(3); 263-269.

(Mao et al., 2015) Indicators of perceived useful dementia care assistive technology: Caregivers' perspectives. Geriatr Gerontol Int 15(8):1049-57.

(Nasreddine et al. 2005) The Montreal cognitive assessment, MoCA: a brief screening tool for mild cognitive impairment. J Am Geriatr Soc, 53:695-699.

(NIH, WHO, 2011) Global Health and Aging http://www.who.int/ageing/publications/global_health.pdf. (05.04.2017).

(Nordheim et al., 2015) Tablet-PC und ihr Nutzen für demenzerkrankte Heimbewohner Z Gerontol Geriat 48:543-549.

(OECD, 2015) Adressing Dementia – the OECD response. OECD publishing, Paris.

(Piano et al., 2014) Real-time Automatic Emotion Recognition from Body Gestures, Proc. IDGEI 2014 Workshop, Eds., L. Paletta, B. Schuller et al., Haifa, Israel. https://doi.org/10.48550/arXiv.1402.5047.

(Podsiadlo et al., 1991) The Timed "Up & Go": A Test of Basic Functional Mobilitv for Frail Elderlv Persons. Journal of the American Geriatric Society, vol. 39, no. 2, pp. 142-148.

(Rheinberg et al., 2001) FAM: Ein Fragebogen zur Erfassung aktueller Motivation in Lern- und Leistungssituationen, *Diagnostica*, 47, pp. 57-66. https://doi.org/10.1026//0012-1924.47.2.57. Hogrefe Verlag.

(Robert Koch-Institut (Hrsg), 2015) Gesundheit in Deutschland. Gesundheitsberichterstattung des Bundes. Gemeinsam getragen von RKI und Destatis. RKI, Berlin.

(Rösler et al., 2003) Overview of Standardised Diagnostic Instruments of Dementia, Fortschritte der Neurologie, Psychiatrie, vol. 71, pp. 187-198.

(Russel et al., 1999) Core affect, prototypical emotional episodes, and other things called emotion: dissecting the elephant. Journal of personality and social psychology.1999; 76(5):805.

(Schneider & Yvon, 2013) A review of multidomain interventions to support healthy cognitive ageing, The Journal of Nutrition, Health & Aging, vol. 17, no. 3, pp. 252-257.

(Schramm et al., 2002) Psychometric properties of Clock Drawing Test and MMSE or Short Performance Test (SKT) in dementia screening in a memory clinic population, International Journal of Geriatric Psychiatry, vol. 17, no. 3, pp. 254-260.

(Schüssler, 2015) Care Dependency and Nursing Care Problems in Nursing Home Residents with and without dementia, doctoral thesis, Medizinische Universität Graz.

(Sheehan et al., 2012) Assessment scales in dementia. Ther Adv Neurol Disord, 5(6):349-58.

(Smarr et al., 2012) Older adults´ preferences for an acceptance of robot assistance for everyday living tasks: 56th Annual Meeting of Human Factors and Ergonomics Society, 22-26 October, Human Factors and Ergonomics Society, Boston, pp. 153 – 157.

(Smith et al. 2005) Measurement of health-related quality of life for people with demetia: development of a new instrument (DEMQOL) and an evaluation of current methodology. Health Technology Assessment. 9(10).

(Span et al., 2013) Involving people with dementia in the development of supportive IT applications: a systematic review. Ageing Res Rev. 12(2):535-51.

(The WHOQOL Group, 1998) Development of the World Health Organization WHOQOL-BREF Quality of Life Assessment: Psychological Medicine, 28(3):551-558.

(University of Illinois, 2019) Modified Interest Checklist (Interessen Checklist). https://www.moho.uic.edu/productDetails.aspx?aid=38 (05.04.2017).

(van der Elst et al., 2006) The Letter Digit Substitution Test: Normative Data for 1,858 Healthy Participants Aged 24–81 from the Maastricht Aging Study (MAAS): Influence of Age, Education, and Sex; Journal of Clinical and Experimental Neuropsychology; 28: 998-1009.

(Wang et al., 2016) Robots to assist daily activities: views of older adults with Alzheimer's disease and their caregivers. Int Psychogeriatr 29(1):67-79.

(Wechsler, 2009) Wechsler Memory Scale – Fourth Edition. Manual. Pearson Assessment, San Antonio, TX.

(Wilson DMC and Ciliska D, 1984) Lifestyle assessment: Development and use of the FANTASTIC checklist. Canadian Family Physician 30: 1527–1532.

(Wu et al., 2014) Acceptance of an assistive robot in older adults: a mixed-method study of human-robot interaction over a 1-month period in the Living Lab setting. Clinical Interventions in Aging, 8(9):801-811.

Anhang 1 Datenerhebungsinstrumente

Gemäß den Richtlinien des Journals wurden urheberrechtlich geschützte Abbildungen aus der veröffentlichten Version entfernt.
